# Electrosynthetic access to unsymmetrical oxaza[8]helicenes with high chiral stability and strong circularly polarized luminescence (CPL)

**DOI:** 10.3762/bjoc.22.25

**Published:** 2026-02-25

**Authors:** Tin Zar Aye, Rubal Sharma, Muthu Karuppasamy, Daiya Suzuki, Haruka Nakajima, Yoshitane Imai, Mitsuhiro Arisawa, Mohamed S H Salem, Shinobu Takizawa

**Affiliations:** 1 SANKEN, The University of Osaka, 8-1 Mihogaoka, Ibaraki-shi, Osaka 567-0047, Japan; 2 Graduate School of Pharmaceutical Sciences, The University of Osaka, 1-6 Yamada-oka, Suita, Osaka 565-0871, Japan; 3 Graduate School of Science and Engineering, Kindai University, 3-4-1 Kowakae, Higashi-Osaka, Osaka 577-8502, Japan; 4 Pharmaceutical Organic Chemistry Department, Faculty of Pharmacy, Suez Canal University, Ismailia 8366004, Egypt

**Keywords:** chemoselectivity, chiroptical, circular dichroism, electrochemistry, emission, helical elongation, helicene, photophysical

## Abstract

Heterohelicenes are compelling chiral π-conjugated scaffolds for optoelectronic and chiral-photonic technologies because their helical frameworks and doped heteroatoms endow them with various photophysical, chiroptical, and electronic merits. However, unsymmetrical heterohelicenes remain rare, as their synthesis is often hindered by chemoselectivity and regioselective control. Here, we exploit the differential redox potentials of two coupling partners as a key player to achieve a chemo- and regioselective electrosynthetic access to a new family of unsymmetrical oxaza[8]helicenes. A controlled anodic sequence enables selective oxidative hetero-coupling followed by dehydrative cyclization, furnishing the extended [8]helical scaffold efficiently under mild, oxidant-free conditions. Structural analyses show retained aromaticity, increased helical distortion, and higher configurational stability (≈38 kcal/mol) relative to their oxaza[7]helicene analogues (<25 kcal/mol). After chiral HPLC separation, the enantiomers display mirror-image CD and strong solution CPL, with |*g_lum_*| up to 2.6 × 10^−3^ and fluorescence brightness up to 30.75 M^−1^ cm^−1^.

## Introduction

Chirality is a pervasive feature of natural and artificial systems, and chiral small molecules continue to underpin advances in chemistry and materials science [1–2]. Among them, helicenes – *ortho*-condensed polycyclic aromatic hydrocarbons (PAHs) built from angularly annulated rings – occupy a distinctive niche because their non-planar, screw-shaped architectures generate inherent, configurational chirality [3–5]. This helicity originates from intramolecular steric congestion and stabilizing π–π interactions between terminal rings, yielding stable enantiomeric conformers with pronounced optical activity. The combination of rigid helical topology and tunable electronic structure has propelled helicenes into diverse applications, spanning chiral photonics [6–9], organic electronics [10–11], molecular machines [12], molecular recognition [13], and bioimaging [14]. Yet, most unsubstituted carbo[*n*]helicenes (*n* ≥ 5) often display modest fluorescence quantum yields, constraining their utility in emissive technologies [15]. Incorporation of heteroatoms to form hetero[*n*]helicenes provides an effective means to modulate frontier orbitals, intermolecular interactions, and excited-state dynamics, frequently enhancing fluorescence efficiency and circularly polarized luminescence (CPL) [16–22]. Consequently, heterohelicenes have emerged as attractive platforms for optoelectronic devices, 3D displays, security inks, and information-storage materials, where both helicity and emission characteristics must be precisely controlled [23–26].

**Scheme 1:**
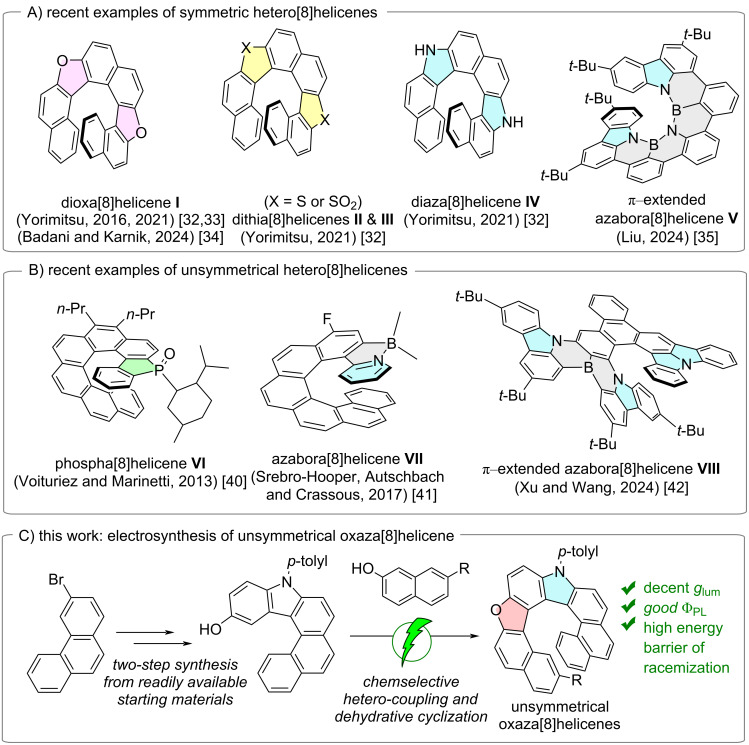
Recent examples of hetero[8]helicenes: (A) symmetric hetero[8]helicenes; (B) unsymmetrical hetero[8]helicenes; (C) short-step electrosynthetic access to new unsymmetrical oxaza[8]helicenes.

A central design element in helicene chemistry is helical extension. Increasing the number of *ortho*-fused rings amplifies π-conjugation, structural rigidity, and chiral stability, typically strengthening chiroptical responses [27–29]; however, it also escalates synthetic difficulty due to heightened strain and more demanding regio- and chemoselective construction. While [7]helicenes have been extensively explored, their [8]helicene counterparts remain comparatively underdeveloped [30], despite the appealing prospect of higher barriers to enantiomerization and richer optoelectronic behavior [31]. In 2021, Yorimitsu and co-workers disclosed a series of symmetric dihetero[8]helicenes **I**–**IV** that exhibited intriguing chiroptical properties utilizing the characteristic transformations of the organosulfur functionality [32–33]. In 2024, Badani, Karnik, and co-workers accessed one member of this class, 7,12-dioxa[8]helicene **I**, through a sequence featuring photochemical *E*–*Z* isomerization, electrocyclization, and oxidative aromatization [34]. In parallel, Liu and co-workers introduced the π-extended azabora[8]helicene **V** with exceptional chiroptical signals and high brightness, emphasizing the promise of this class of chiral molecules (Scheme 1A) [35].

Despite these advances, unsymmetrical hetero[8]helicenes – where heteroatoms occupy non-equivalent positions along the helical rim – are far rarer. Their scarcity primarily reflects the formidable challenge of controlling chemo- and regioselectivity during ring annulation and heteroatom introduction, which often necessitates multistep synthetic strategies [36–38]. To the best of our knowledge, only four examples have been reported so far [39–42]. Recent reports suggest that breaking symmetry can further amplify CPL responses and enable finer electronic tuning [39]. Voituriez, Marinetti and co-workers developed a phosphorus-embedded [8]helicene **VI** [40], Crassous and co-workers prepared azabora[8]helicene **VII** via cycloborylation of a pyridine-substituted carbo[6]helicene [41], while Xu, Wang and colleagues reported a multistep route to azabora[8]helicene **VIII** (Scheme 1B) [42]. Collectively, these studies highlight both the synthetic bottleneck and the untapped potential of unsymmetrical hetero[8]helicenes.

Inspired by the superior selectivity and sustainability of organic electrosynthesis as an eco-friendly alternative to conventional oxidative methods [43–47], we leveraged our electrochemical approaches [48–52], and redesigned the synthons to access a new unsymmetrical hetero[8]helicene (Scheme 1C). This strategy delivers the target scaffold in only three steps from commercially available substrates and exploits the differential oxidation potentials of the two partners to enforce chemoselective cross-annulation. To the best of our knowledge, this represents the shortest route reported to any unsymmetrical hetero[8]helicene. We then investigated the structural, photophysical and chiroptical properties of these new oxaza[8]helicenes and benchmarked their behavior against their corresponding oxaza[7]helicene analogues.

## Results and Discussion

### Electrosynthesis of unsymmetrical oxaza[8]helicenes

**Scheme 2:**
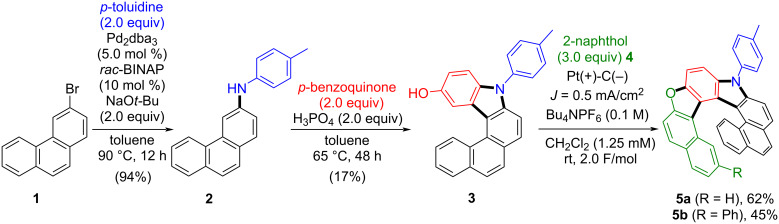
Short-step synthesis of unsymmetrical oxaza[8]helicenes **5**.

Building on Zhang’s facile acid-mediated carbazole synthesis [53], in which aniline derivatives react with *p*-benzoquinone to afford 3-hydroxycarbazoles [54], we employed a closely related substrate. Specifically, *N*-(*p*-tolyl)phenanthren-3-amine (**2**) – prepared from 3-bromophenanthrene (**1**) via Buchwald–Hartwig amination with *p*-toluidine under Pd catalysis [55] – was subjected to phosphoric acid-mediated annulation with *p*-benzoquinone to give the hydroxycarbazole derivative **3** through a tandem Michael addition/ring-closure sequence. After rapid optimization of key parameters (see Supporting Information File 1), we developed a one-pot electrochemical annulation between **3** and β-naphthol derivative **4**. Using *n*-Bu_4_NPF_6_ as the electrolyte in CH_2_Cl_2_ at room temperature, this protocol furnished oxaza[8]helicenes **5** in good-to-moderate yields with >75% Faradaic efficiency, and no homo-coupling products were detected under the optimized conditions (Scheme 2).

**Figure 1:**
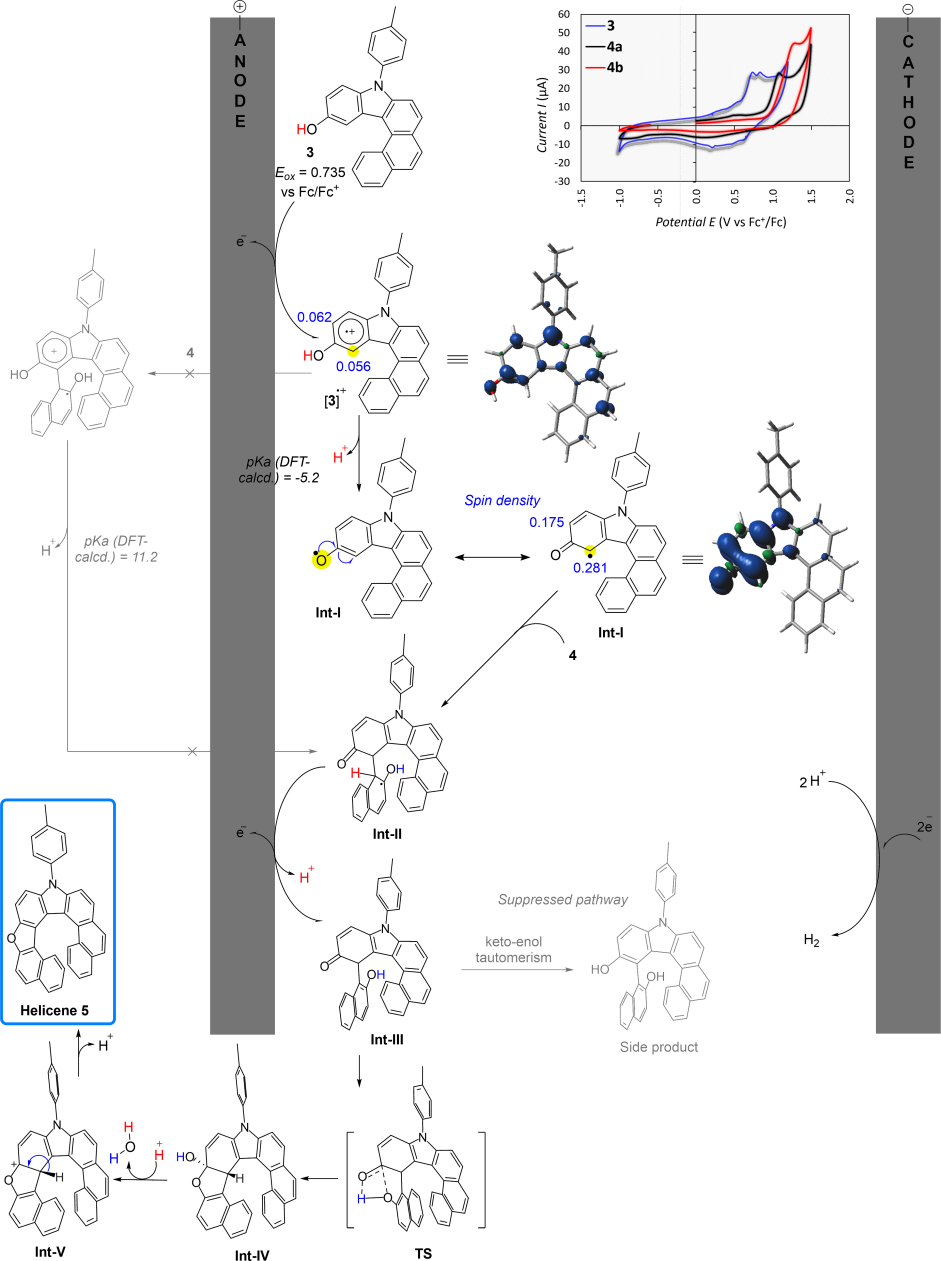
A plausible reaction mechanism: cyclic voltammetry (CV) analyses of hydroxycarbazole derivative **3** and 2-naphthol derivatives **4**; DFT-based calculations of the p*K*_a_ of [**3**]**^·^**^+^ radical cation and spin density of neutral radical intermediate **Int-I** (optimized at the UB3LYP/6-31G+(d,p) level of theory with IEPCM model as solvation of DCM. Grimme’s dispersion with the original D3 damping function was applied as empirical dispersion correction to the optimized structures).

Based on our previous reports [48,56], DFT calculations, and cyclic voltammetry (CV) analyses (Figure 1), anodic single-electron transfer (SET) is expected to occur first from **3**, generating the electrophilic radical cation [**3**]**^·^**^+^ as **3** (*E_ox_* = 0.735 V vs Fc/Fc^+^ in CH_2_Cl_2_) is oxidized more readily than the 2-naphthol partners (*E_ox_* of **4a** = 1.081 V and *E_ox_* of **4b** = 1.286 V vs Fc/Fc^+^ in CH_2_Cl_2_). The radical cation [**3**]**^·^**^+^ then undergoes rapid deprotonation to form a neutral radical intermediate (**Int-I**) with high spin density at the reactive site, enabling regioselective intermolecular coupling with **4**. While a Scholl-type coupling-first scenario cannot be ruled out, the computed acidity of [**3**]**^·^**^+^ (p*K*_a_ ≈ −5.2) together with the more spin-density localization in **Int-I** supports a deprotonation-first, neutral-radical pathway, consistent with related electrochemical arenol activations reported by Waldvogel and co-workers [46]. Subsequent intramolecular dehydrative cyclization furnishes the desired oxaza[8]helicenes **5**. The oxidation-potential gap between **3** and **4** and the reactivity of **Int-I** thus provides a handle to control chemo- and regioselectivity.

### Structural properties of oxaza[8]helicenes

#### Aromaticity

**Figure 2:**
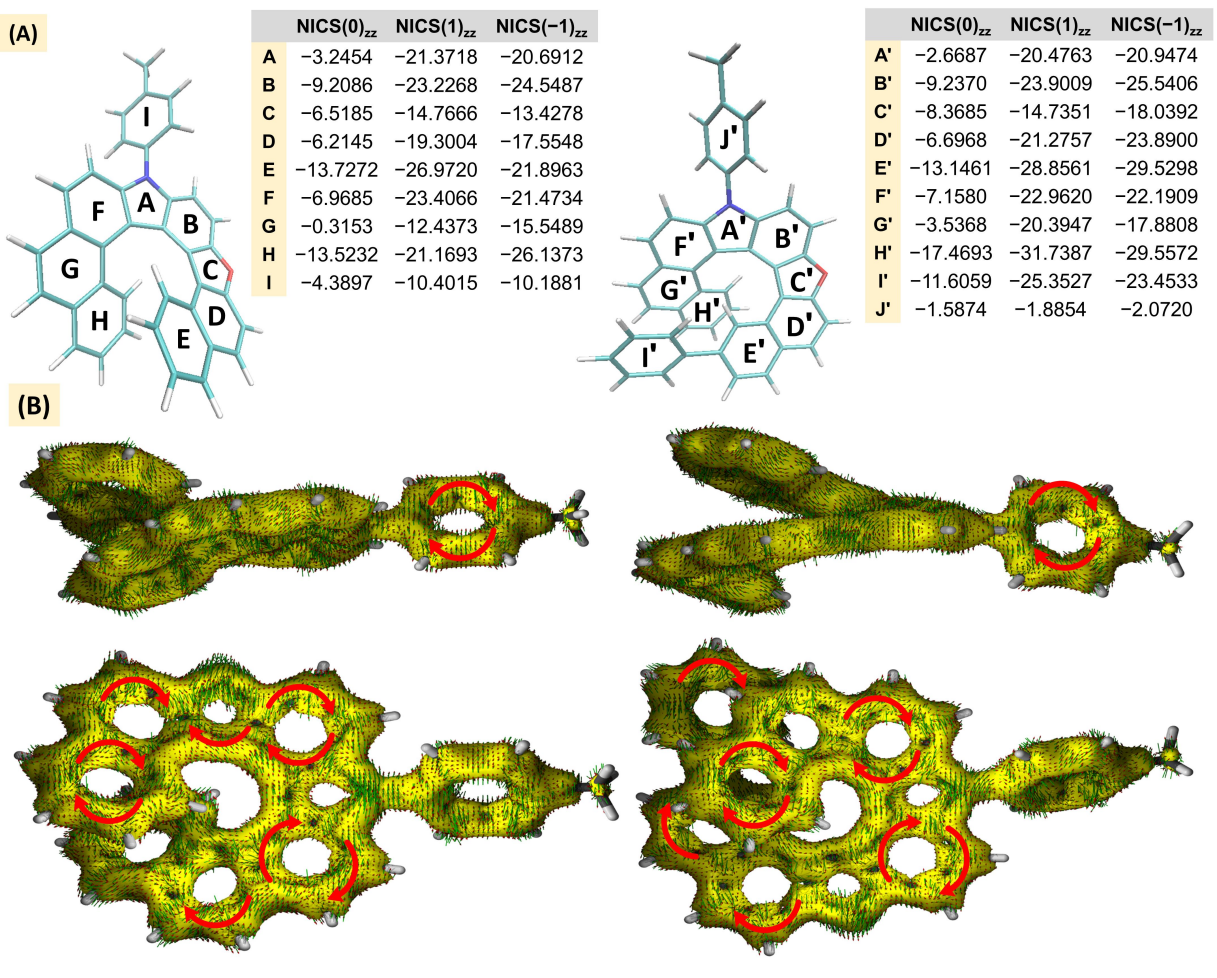
Aromaticity of oxaza[8]helicenes: (A) NICS(0)_zz_ and NICS(1)_zz_ values of **5a** and **5b** calculated at MN15/6-311G(2d,p)/SMD=chloroform level of theory; (B) ACID plots calculated at the B3LYP/6-311G(d,p) level of theory (isosurface value: 0.05).

We evaluated the aromaticity of the oxaza[8]helicenes **5a** and **5b** using nucleus-independent chemical shifts (NICS), as proposed by Schleyer and co-workers [57–58]. As shown in Figure 2A, the terminal rings exhibit higher aromatic character and a progressive increase in aromaticity upon helical elongation compared with the corresponding oxaza[7] analogues (see Supporting Information File 1) [49], as evidenced by NICS(1)_zz_ values of −26.97 and −21.17 for rings E and H in **5a**, and −28.86 and −31.74 for rings E′ and H′ in **5b**. This enhancement can be attributed to magnetic coupling between the face-to-face terminal rings, in line with the Johnson–Bovey model, given the inter-ring distances of approximately 3.75–3.79 Å [59]. On the other hand, the aromaticity of the pyrrole rings (A and A′) and benzene rings G and G′ in **5a** and **5b** decreases, which can be attributed to the increased deviation from planarity, particularly in the more extended helicenes, consistent with previous studies [60].

To gain further insight, we performed anisotropy of the induced current density (AICD) calculations for **5a** and **5b** at the B3LYP/6-311G(d,p) level in the gas phase [61]. The resulting plots show a clockwise diatropic current along the fused heterocycles and benzene rings, in agreement with the ring-current patterns reported for other helicene scaffolds [27] (Figure 2B).

#### Enantiomerization barriers of oxaza[7]helicenes

**Figure 3:**
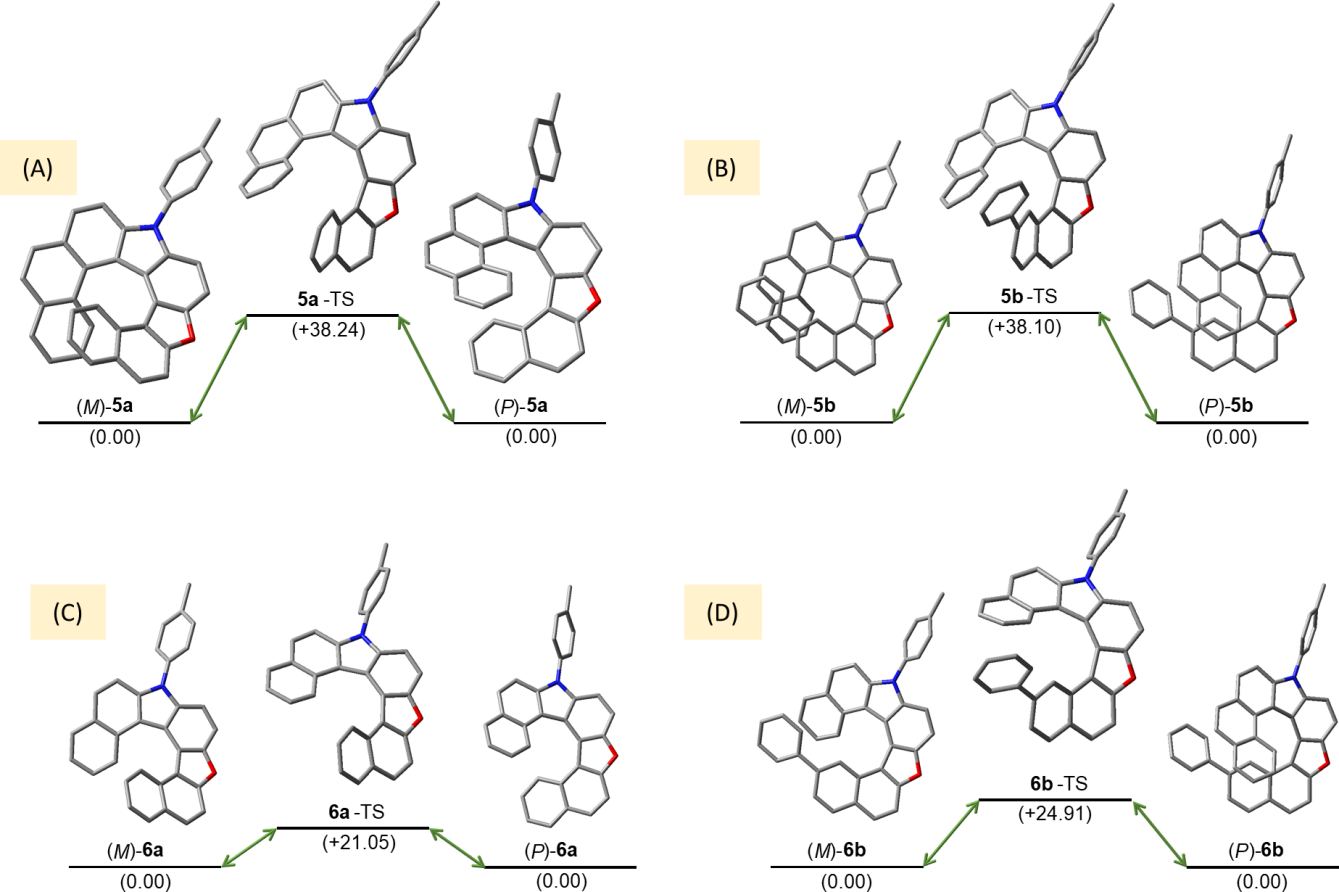
(*P*/*M*) Enantiomerization process of **5a** (A), **5b** (B), **6a** (C), and **6b** (D); relative Gibbs free energies were calculated in (kcal mol^−1^) at the MN15/6-31G(2d,p)/SMD=chloroform level of theory.

To investigate the enantiomerization (*P*/*M*) barriers of oxaza[8]helicenes **5a** and **5b**, we performed DFT calculations to locate the transition states with the highest Gibbs free energies. In both cases, the transition states correspond to conformations in which the terminal rings adopt a face-to-face arrangement along the helical axis (Figure 3). The calculated enantiomerization barriers for **5a** and **5b** are 38.24 and 38.10 kcal mol^−1^, respectively (Figure 3A and 3B), highlighting the pronounced effect of π-extension on the rigidity of the helical backbone. In contrast, the corresponding oxaza[7]helicenes **6a** and **6b** exhibit significantly lower barriers of 21.05 and 24.91 kcal mol^−1^, respectively (Figure 3C and 3D), which leads to rapid enantiomerization within a few hours at room temperature and severely limits their applicability in chiroptical devices despite their favorable CD and CPL properties (vide infra). By comparison, the markedly higher (*P*/*M*) enantiomerization barriers of **5a** and **5b** translate into excellent configurational robustness, as demonstrated by the absence of detectable enantiomerization when solutions of (*M*)-**5a** were heated at 130 °C for 2.5 h.

### Optical properties of oxaza[8]helicenes

#### Photophysical features

**Figure 4:**
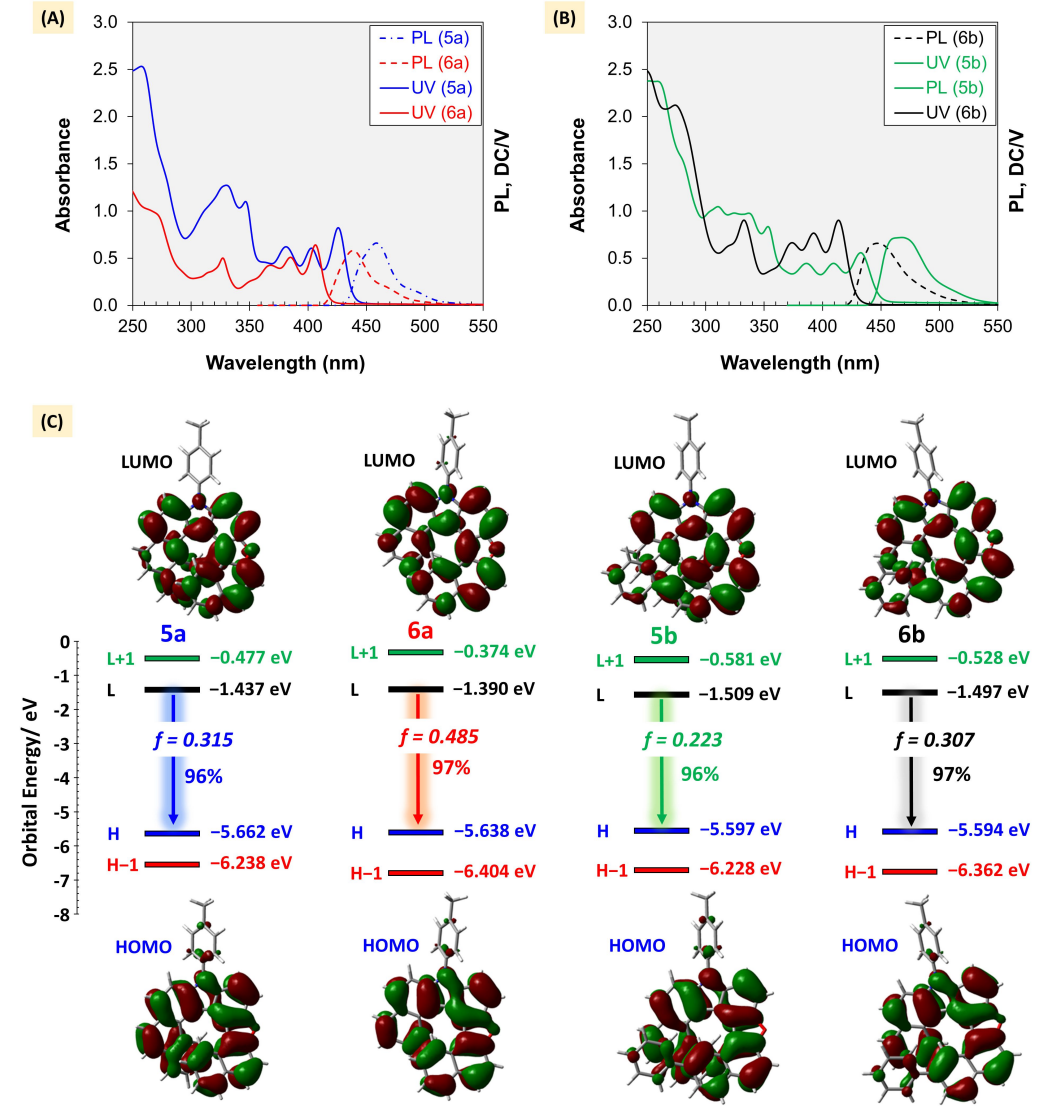
Photophysical characters of oxaza[*n*]helicenes: (A) and (B) UV–vis absorption and PL spectra; (C) Frontier Kohn–Sham molecular orbitals (HOMO and LUMO) optimized in the lowest energy excited state (S_1_) and TD-DFT calculated transitions at MN15/6-311G(2d,p)/SMD=chloroform level of theory.

The absorption and emission spectra of oxaza[8]helicenes **5a** and **5b** in chloroform (1 × 10^−5^ M) were recorded and compared with those of the corresponding oxaza[7]helicenes **6a** and **6b** (Figure 4A and 4B). As expected, extension of the helical π-systems in **5** leads to enhanced conjugation relative to **6**, manifested in red-shifted absorption and emission bands. In chloroform, **5a** shows a pronounced higher absorption peak at 426 nm (ε = 8.23 × 10^4^ M^−1^ cm^−1^) with an optical indirect bandgap (*E_g_*) of 2.78 eV. Similarly, **5b**, **6a**, and **6b** exhibit their higher absorption peaks at 433 nm (ε = 5.59 × 10^4^ M^−1^ cm^−1^), 407 nm (ε = 6.43 × 10^4^ M^−1^ cm^−1^), and 414 nm (ε = 9.02 × 10^4^ M^−1^ cm^−1^), with corresponding optical indirect bandgaps *E_g_* of 2.70, 2.93, and 2.85 eV, respectively (see Supporting Information File 1). The photoluminescence (PL) spectra in chloroform display emission maxima at 459 and 468 nm for **5a** and **5b**, compared to 439 and 447 nm for **6a** and **6b**.

To gain insight into the electronic transitions, we performed time-dependent DFT (TD-DFT) calculations for all oxaza[7]helicenes **6** and oxaza[8]helicenes **5** after geometry optimization at the S_1_ minimum (see Supporting Information File 1 and Supporting Information File 2) [62–63]. The convergence of these structures was confirmed by frequency analysis, which revealed no imaginary frequencies. The frontier orbitals are non-degenerate, and the S_1_ → S_0_ transition is dominated by the LUMO → HOMO contribution. The oscillator strength of the S_1_ → S_0_ transition decreases with helical elongation (Figure 4C), in line with the observed trends in emission efficiency. Fluorescence quantum yields (Φ*_f_*, in chloroform, 1 × 10^−3^ M) for **5a** and **5b** were 25.1% and 22.6%, slightly lower than those of **6a** (40.5%) and **6b** (38.9%). This difference can be rationalized by their radiative rate constants (*k*_f_). The calculated *k*_f,calcd_ values are 0.174, 0.159, 0.243, and 0.194 ns^−1^ for **5a**, **5b**, **6a**, and **6b**, respectively (see Supporting Information File 1). According to Φ_f_ = *k*_f_ /( *k*_f_ + *k*_nr_), where *k*_nr_ is the non-radiative rate constant, the balance between these decay pathways accounts for the observed variation in quantum yields [64].

#### Chiroptical features

**Figure 5:**
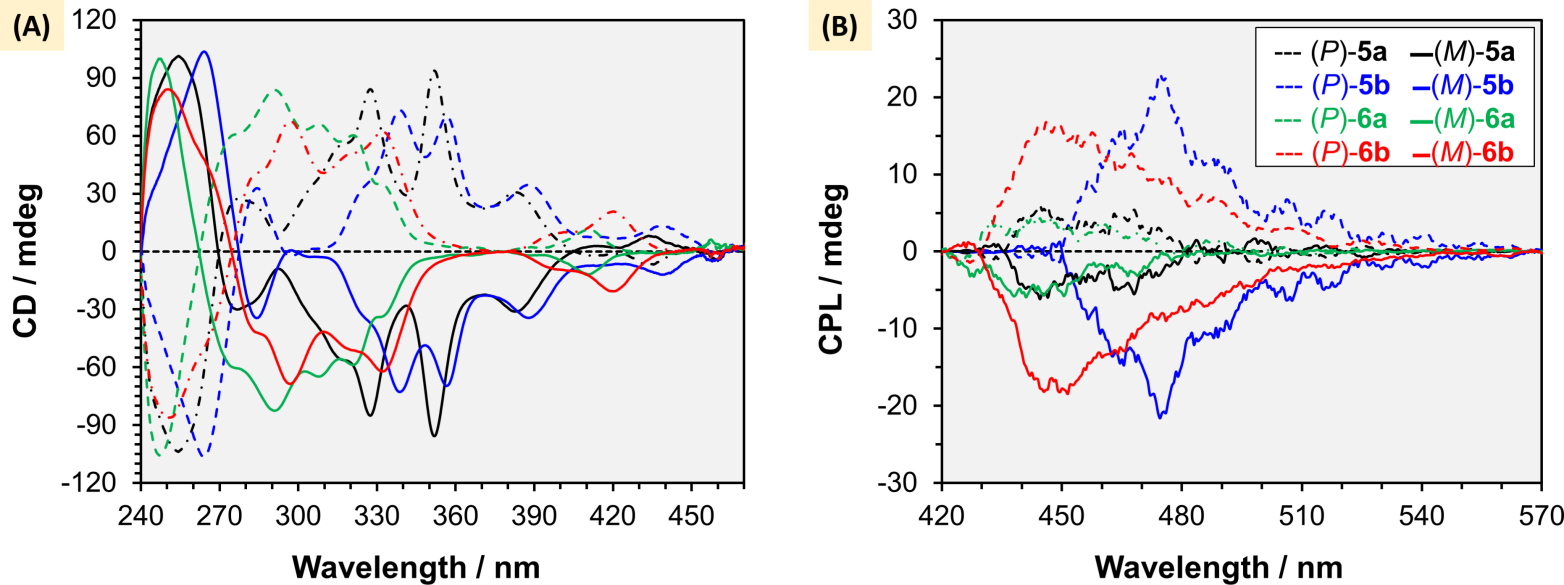
Chiroptical properties of oxaza[*n*]helicenes: (A) CD spectra measured in chloroform (1 × 10^−5^ M); CPL spectra measured in chloroform (1 × 10^−3^ M). Solid lines for (*M*)-configuration and dashed lines for (*P*)-configuration of **5a** (black), **5b** (blue), **6a** (green), and **6b** (red).

The higher enantiomerization barriers of (*P*/*M*)-**5a** and (*P*/*M*)-**5b** enabled complete separation of their enantiomers by HPLC using a Daicel Chiralpak IA column (see Supporting Information File 1). Despite the rapid enantiomerization of (*P*/*M*)-**6a** and (*P*/*M*)-**6b**, we were able to separate the two enantiomers at lower temperature and samples were stored at −20 °C before their chiroptical responses were evaluated. The optical purities of (*P*/*M*)-**6a** and (*P*/*M*)-**6b** measured samples were confirmed to be >97% ee, confirming the reliability of our results. However, this low enantiomerization barriers of oxaza[7]helicenes **6a**,**b** hinders their practical applications. The CD spectra of optically pure **5a**,**b** and **6a**,**b** were recorded (Figure 5A), and compared with reported analogous oxaza[7]helicenes [49], and spectra obtained from TD-DFT to assign their absolute configurations [27]. The absolute configurations in the first and second fractions of the chiral HPLC analysis were assigned as the (*P*)- and (*M*)-enantiomers, respectively, for all **5a**,**b** and **6a**,**b**. As expected, the increase in helical length (*n*) from 7 to 8, **5a** and **5b** exhibited more red-shifted maximum |*g*_abs_| values at around 350 nm, whereas **6a** and **6b** showed values around 290–300 nm for both enantiomers (Figure 5A). High |*g*_abs_| values have also been reported for π-extended helical nanographenes featuring aza[7]helicene subunits [65].

Subsequently, CPL spectra of (*P*/*M*)-**5a**,**b** and (*P*/*M*)-**6a**,**b** were measured to evaluate the potential of these oxaza[*n*]helicenes as chiral emitters. The |*g*_lum_| values were determined to be 0.001 at 498.0 nm for **5a**, 0.0026 at 474.5 nm for **5b**, 0.0006 at 441.8 nm for **6a**, and 0.0018 at 448.8 nm for **6b**, with the (*P*)-configuration exhibiting a positive Cotton effect and the (*M*)-configuration showing a negative Cotton effect (Figure 5B). According to the theory [66], the luminescence dissymmetry factor |*g_lum_*| can be determined by Equation 1:


[1] $$\left|g\right|=\frac{4\cdot \left|\mu \right|\cdot \left|m\right|\cdot \cos\theta \mu ,m}{{\left|\mu \right|}^{2}+{\left|m\right|}^{2}}$$

Therefore, the electric transition dipole moments (ETDM) (μ) and magnetic transition dipole moments (MTDM) (*m*), as well as the angle (θ) between μ and *m*, of (*M*)-**5a**,**b** and (*M*)-**6a**,**b** for their S_1_ → S_0_ transitions were obtained by TD-DFT calculations (see Supporting Information File 1). For most organic CPL-emitters, the |*m*| values are typically much smaller than the |μ| values (Table 1). The above equation can thus be simplified as Equation 2:


[2] $$\left|g\right|=\frac{4\cdot \left|m\right|\cdot \cos\theta \mu ,m}{\left|\mu \right|}$$

Hence, the lower |μ| and larger cos θ values of **5b** lead to an approximately 1.5-fold increase in their calculated *g*_cal_ compared to corresponding oxaza[7]helicene **6b** and around 2.5-fold increase in *g*_cal_ compared to the unsubstituted oxaza[8]helicene **5a** (Table 1), which is consistent with the trend observed experimentally (Figure 5B). With the chiroptical results and Φ_f_ in hand, the brightness *B*_CPL_ values were calculated to be 30.75 M^−1^ cm^−1^ for **5b** and 31.46 M^−1^ cm^−1^ for **6b**. This comprehensive understanding of the influence of phenyl substitution and helical extension on the CPL features of oxaza[*n*]helicenes provides a valuable roadmap for designing future CPL- emitters that integrate synthetic accessibility with superior chiral stability and chiroptical performance.

**Table 1:** Chiroptical features of oxaza[8]helicenes **5** and oxaza[7]helicenes **6**.

	S_0_ → S_1_ transition						CPL	

Oxaza[*n*]helicene	ETDM^a^ |μ| (10^−20^ esu cm)	MTDM^b^ |*m*| (10^−20^ erg G^−1^)	θ_μ,m_ (deg)^c^	(R)^d^ (10^−40^ erg esu cm G^−1^)	*g*_cal_^e^ (10^−3^)	λ_em_ (nm)	*g*_lum_^e^ (10^−3^)^f^	*B*_CPL_ M^−1^ cm^−1^

(*M*)-**5a**	553.8	1.20	95.9	−68.71	−0.90	498.0	−1.0	15.98
(*M*)-**5b**	502.4	1.81	98.7	−137.30	−2.18	474.5	−2.6	30.75
(*M*)-**6a**	665.4	1.06	94.5	−54.84	−0.50	441.8	−0.6	6.13
(*M*)-**6b**	569.0	1.87	96.5	−121.02	−1.50	448.8	−1.8	31.46

^a^Electric transition dipole moments (ETDM) for the S_1_ → S_0_ transitions; ^b^magnetic transition dipole moments (MTDM) for the S_1_ → S_0_ transitions; ^c^the angle between ETDM and MTDM vectors; ^d^rotational strength; ^e^dimensionless values calculated at the MN15/lanl2mb (iefpcm = chloroform); ^f^measured dissymmetry factors.

## Conclusion

In summary, we have established an electrosynthetic strategy to access a novel class of unsymmetrical oxaza[8]helicenes by exploiting the differential oxidation potentials of appropriately designed coupling partners to control both chemo- and regioselectivity. A finely tuned anodic sequence enables selective oxidative hetero-coupling followed by dehydrative cyclization, delivering extended [8]helical scaffolds efficiently under mild, oxidant-free conditions. Combined experimental and DFT analyses reveal that these oxaza[8]helicenes retain the aromatic character of the π‐framework while exhibiting significantly enhanced configurational stability compared to their oxaza[7] congeners, with enantiomerization barriers of up to ≈38 kcal mol^−1^. After chiral separation, the enantiomers display intense chiroptical responses, including mirror-image CD and strong CPL with |*g*_lum_| values up to 0.0026 and CPL brightness approaching 30.8 M^−1^ cm^−1^.

## Supporting Information

File 1 Experimental procedures, synthetic details, NMR spectra, chiral HPLC chromatograms, DFT and TD-DFT calculations.

File 2 Cartesian coordinates of DFT calculations.

## Data Availability

All data that supports the findings of this study is available in the published article and/or the supporting information of this article.
