# Supplementary material for: Electrosynthetic access to unsymmetrical oxaza[8]helicenes with high chiral stability and strong circularly polarized luminescence (CPL)
Source: Beilstein J Org Chem. 2026 Feb 25;22:372–82. doi: 10.3762/bjoc.22.25 (PMC12951319; doi:10.3762/bjoc.22.25)
Supplement: File 1 — Experimental procedures, synthetic details, NMR spectra, chiral HPLC chromatograms, DFT and TD-DFT calculations. [file Beilstein_J_Org_Chem-22-372-s001.pdf]

BEILSTEIN JOURNAL OF ORGANIC CHEMISTRY

Supporting Information

for

Electrosynthetic access to unsymmetrical oxaza[8]helicenes with high chiral stability and strong circularly polarized luminescence (CPL)

Tin Zar Aye, Rubal Sharma, Muthu Karuppasamy, Daiya Suzuki, Haruka Nakajima, Yoshitane Imai, Mitsuhiro Arisawa, Mohamed S. H. Salem and Shinobu Takizawa

Beilstein J. Org. Chem. **2026,** *22,* 372–382. doi:10.3762/bjoc.22.25

Experimental procedures, synthetic details, NMR spectra, chiral HPLC chromatograms, DFT and TD-DFT calculations

License and Terms: This is a supporting information file under the terms of the Creative Commons Attribution License (https://creativecommons.org/licenses/by/4.0). Please note that the reuse, redistribution and reproduction in particular requires that the author(s) and source are credited and that individual graphics may be subject to special legal provisions.
The license is subject to the *Beilstein Journal of Organic Chemistry* terms and conditions: (https://www.beilstein-journals.org/bjoc/terms)

Contents

1. Materials and methods (synthetic procedures and spectra) 2
*2. HPLC Chiral resolution of compounds **5a,b** and **6a,b*** 10
3. *DFT and TD-DFT calculations to study the optoelectronic features* 14
*3.1. Molecular orbitals of **5** and **6*** 15
*3.2. Aromaticity of oxaza[8]helicenes **5a** and **5b** and oxaza[7]helicenes **6a** and **6b*** 23
3.3. Time-dependent density-functional theory (TD-DFT) calculations 36
4. C*alculation of band gap energy from UV–vis absorption (Tauc Plots)* 46

1. Materials and methods (synthetic procedures and spectra)

1.1. General experimental details

^{1}H-, and ^{13}C NMR spectra were recorded with JEOL JMN ECS 400 FT NMR or Bruker AVANCE II (^{1}H NMR 400 MHz, 600 MHz, and 700 MHz ^{13}C NMR 101 MHz, 151 MHz, or 176 MHz). ^{1}H NMR spectra are reported as follows: the chemical shift in ppm downfield of tetramethylsilane (TMS) and referenced to residual solvent peak ($CDCl_3$) at 7.26 ppm, multiplicities (s = singlet, d = doublet, dd = doublet of doublets, t = triplet, q = quartet, m = multiplet), coupling constants (Hz) and integration. ^{13}C NMR spectra were reported in ppm relative to the central line of triplet for $CDCl_3$ at 77.16 ppm. APCI−MS spectra were obtained with JMS−T100LC (JEOL). FT-IR spectra were recorded on a JASCO FT−IR system (FT/IR4100). UV–Vis absorption spectra were obtained on a JASCO V−670 spectrophotometers. CD spectra were recorded on a JASCO J−1500 spectropolarimeter. Emission spectra and CPL spectra were obtained at room temperature using a JASCO CPL − 300 spectrofluoropolarimeter (Tokyo, Japan). The absolute PL quantum yields were measured using an Absolute PL Quantum Yield Measurement System (C9920–02, Hamamatsu Photonics [Hamamatsu, Japan]) in the air at room temperature. All simple chemicals and solvents were purchased from commercial suppliers and used without further purification.

1.2. General procedure for the synthesis of N-(p-tolyl)phenanthren-3-amine **2**

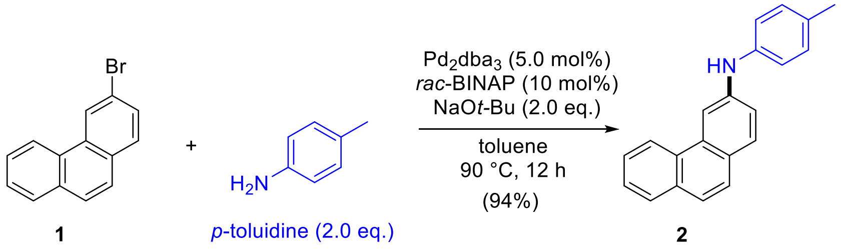

A toluene solution (30 mL) of **1**, *p*-toluidine (2.0 equiv), $Pd_2(dba)_3$ (5.0 mol %), BINAP (10 mol %), and NaO*t*-Bu (2.0 equiv) was stirred at 90 °C under N_2 atmosphere. After stirring for 12 h, the reaction mixture was filtered and the filtrate was directly purified on silica-gel to give **2** as a yellowish white solid (94% yield).

^{1}H NMR (400 MHz, $CDCl_3$) δ 8.52-8.55 (m, 1H), 8.28 (d, J = 2.3 Hz, 1H), 7.88-7.91 (m, 1H), 7.79 (d, J = 8.7 Hz, 1H), 7.69 (d, J = 8.7 Hz, 1H), 7.60-7.62 (m, 3H), 7.30 (dd, J = 8.5, 2.1 Hz, 1H), 7.16-7.22 (m, 4H), 5.90 (s, 1H), 2.41 (s, 3H).

^{13}C NMR (101 MHz, $CDCl_3$) δ 142.78, 140.30, 132.71, 131.78, 131.50, 130.16, 129.83, 129.72, 128.62, 126.77, 126.71, 126.64, 126.10, 124.17, 122.85, 119.30, 118.50, 107.96, 20.89.

HRMS (APCI): calcd for $C_{21}H_{18}N^{+}$: m/z 284.1434 [M + H]+, found 284.1430.

IR (KBr): 3399, 3033, 2913, 1614, 1518, 1324, 809, 751 cm^{-1}.

m.p.: 121 – 122 °C.

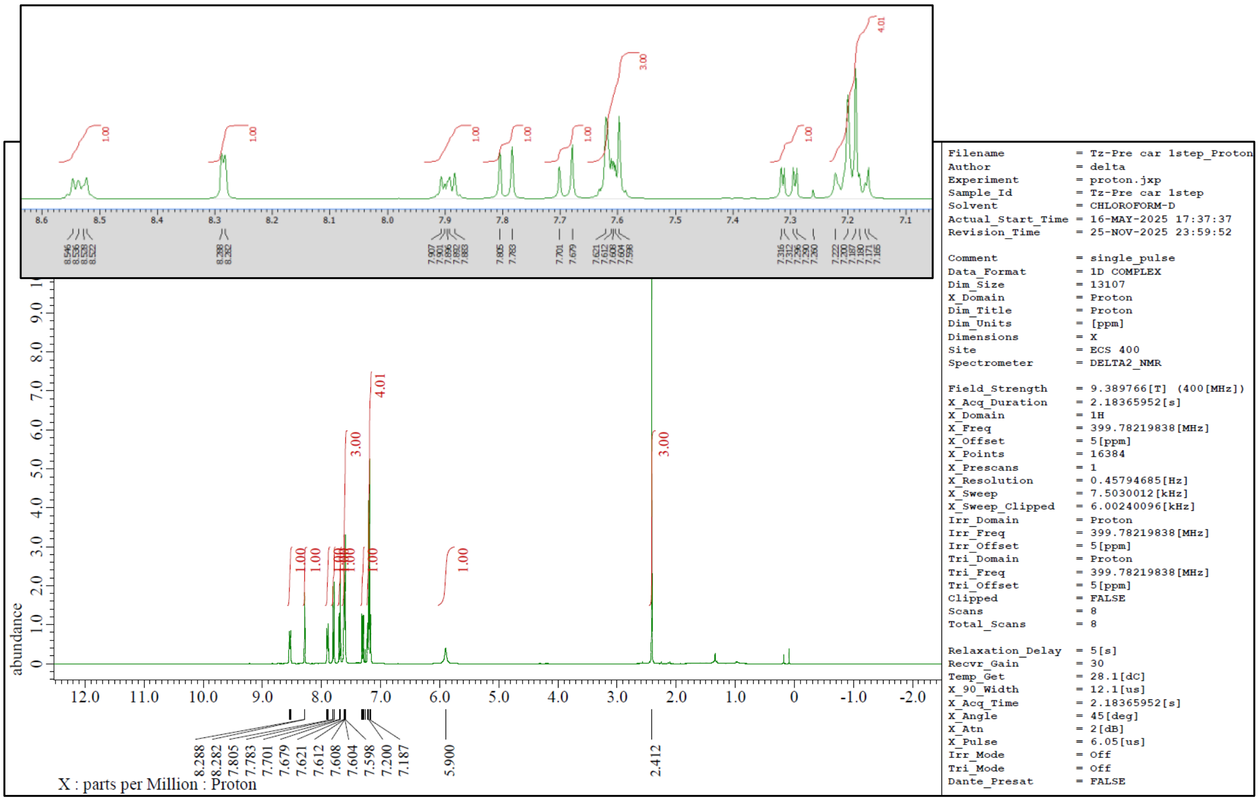

2 (^{1}H NMR, 400 MHz, $CDCl_3$).

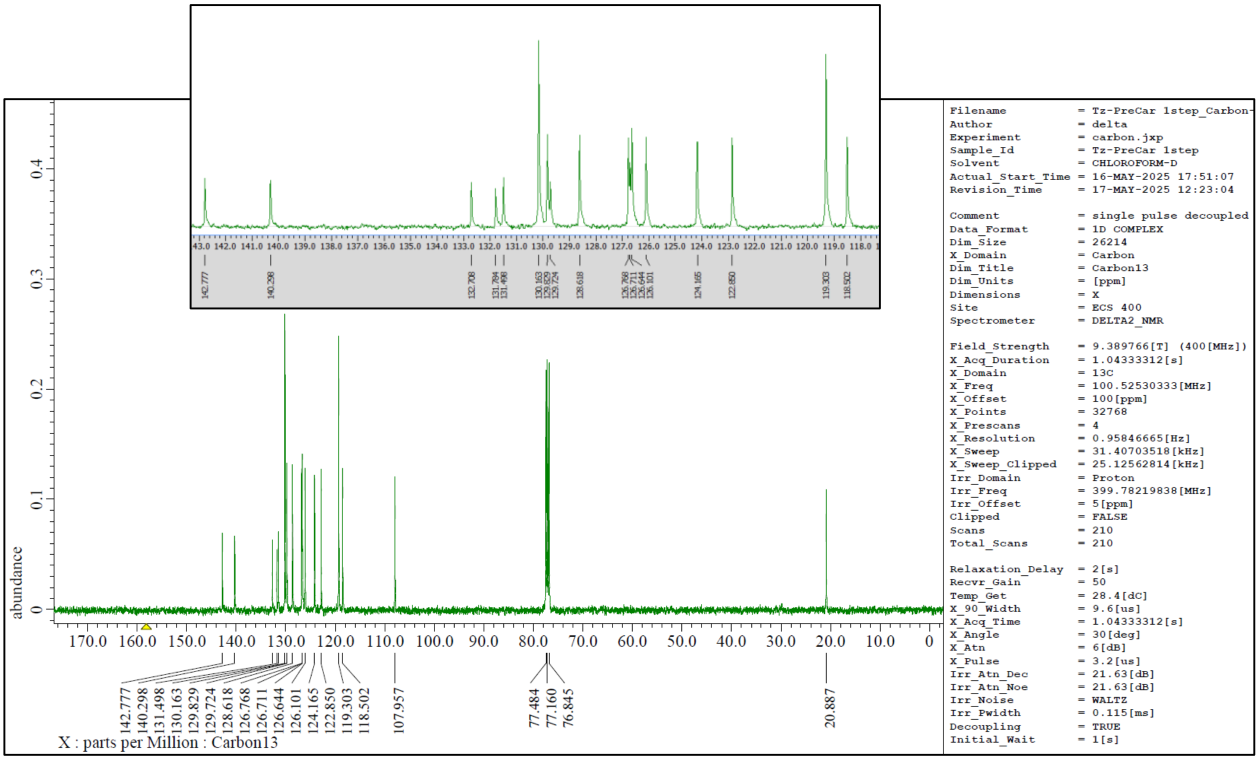

2 (^{13}C NMR, 101 MHz, $CDCl_3$).

*1.3. General procedure for the synthesis of 9-(p-tolyl)-9H-naphtho[2,1-c]carbazol-12-ol **3***

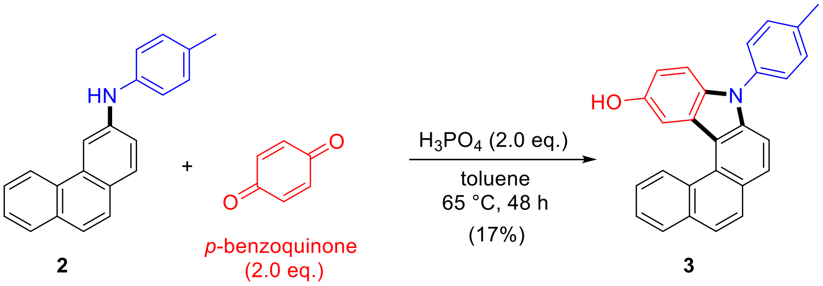

To a solution of **2** and *p*−benzoquinone (2.0 equiv) in dry toluene (25 mL), *ortho*-phosphoric acid (2.0 equiv) dissolved in (25 mL) toluene was added dropwise. The reaction mixture was stirred at 65 °C for 48 h under N_2 atmosphere until its completion. Next, the reaction was quenched via water, extracted with EtOAc and the combined organic extracts dried over Na_2SO_4, and evaporated in vacuo. The crude mixture was purified on silica column chromatography (eluent: *n*−hexane/DCM/ethyl acetate) to give double **3** as a white solid in 17% yield.

^{1}H NMR (600 MHz, $CDCl_3$) *δ* 9.32-9.34 (m, 1H), 8.27 (d, *J* = 2.7 Hz, 1H), 8.00 (dd, *J* = 7.9, 1.7 Hz, 1H), 7.84 (d, *J* = 8.9 Hz, 1H), 7.80 (d, *J* = 8.9 Hz, 1H), 7.75 (d, *J* = 8.2 Hz, 1H), 7.64-7.69 (m, 2H), 7.55 (d, *J* = 8.2 Hz, 1H), 7.43-7.47 (m, 4H), 7.32 (d, *J* = 8.2 Hz, 1H), 7.02 (dd, *J* = 8.9, 2.7 Hz, 1H), 4.79 (s, 1H), 2.52 (s, 3H).

^{13}C NMR (151 MHz, $CDCl_3$) *δ* 148.72, 141.90, 138.14, 136.59, 134.93, 133.30, 130.72, 129.56, 128.04, 127.95, 127.91, 127.63, 127.60, 127.40, 126.67, 124.68, 124.39, 124.21, 116.62, 114.52, 111.46, 111.13, 108.95, 21.43 (One carbon overlapped).

HRMS (APCI): calcd for $C_{27}H_{20}NO^+$: m/z 374.1539 [M + H]+, found 374.1538.

IR (KBr): 3331, 3040, 2922, 1512, 1449, 1342, 1196, 830 cm^{-1}.

mp: 210 – 211 °C.

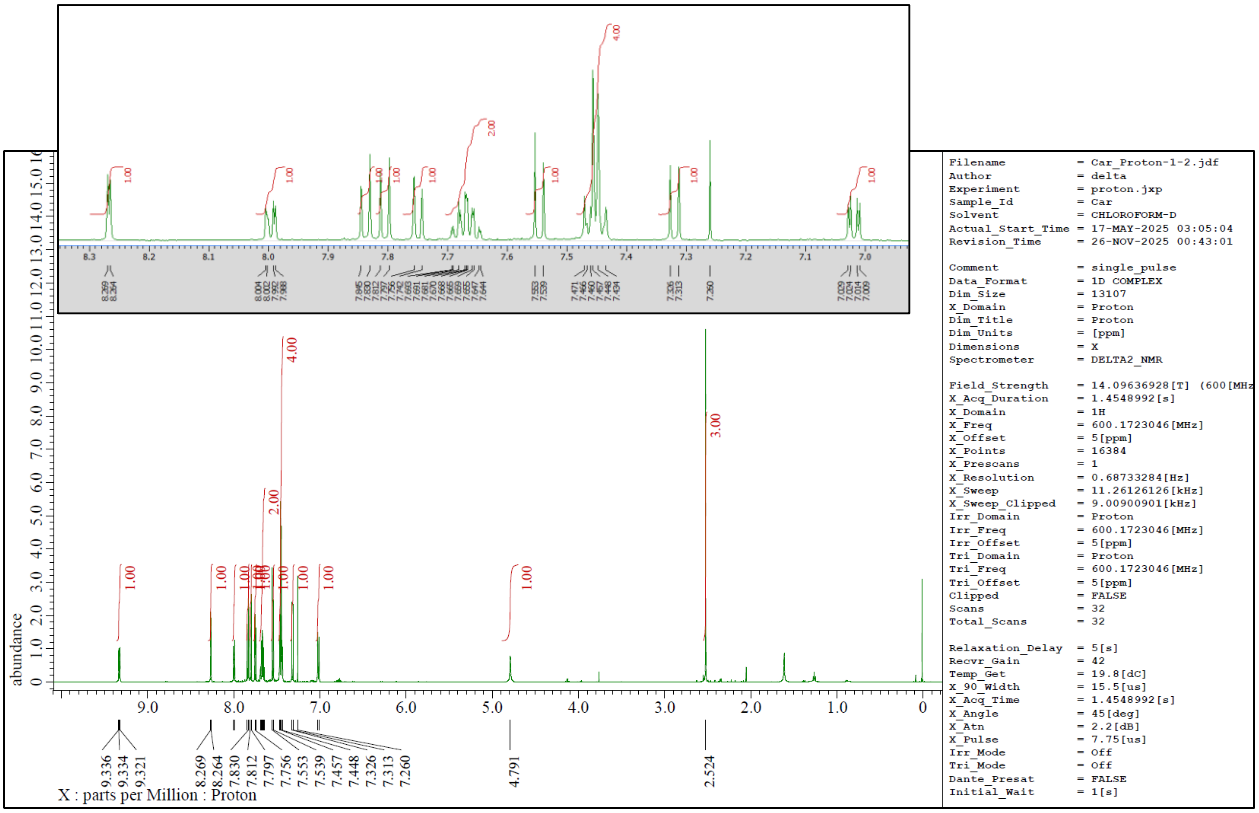

3 (^{1}H NMR, 600 MHz, $CDCl_3$).

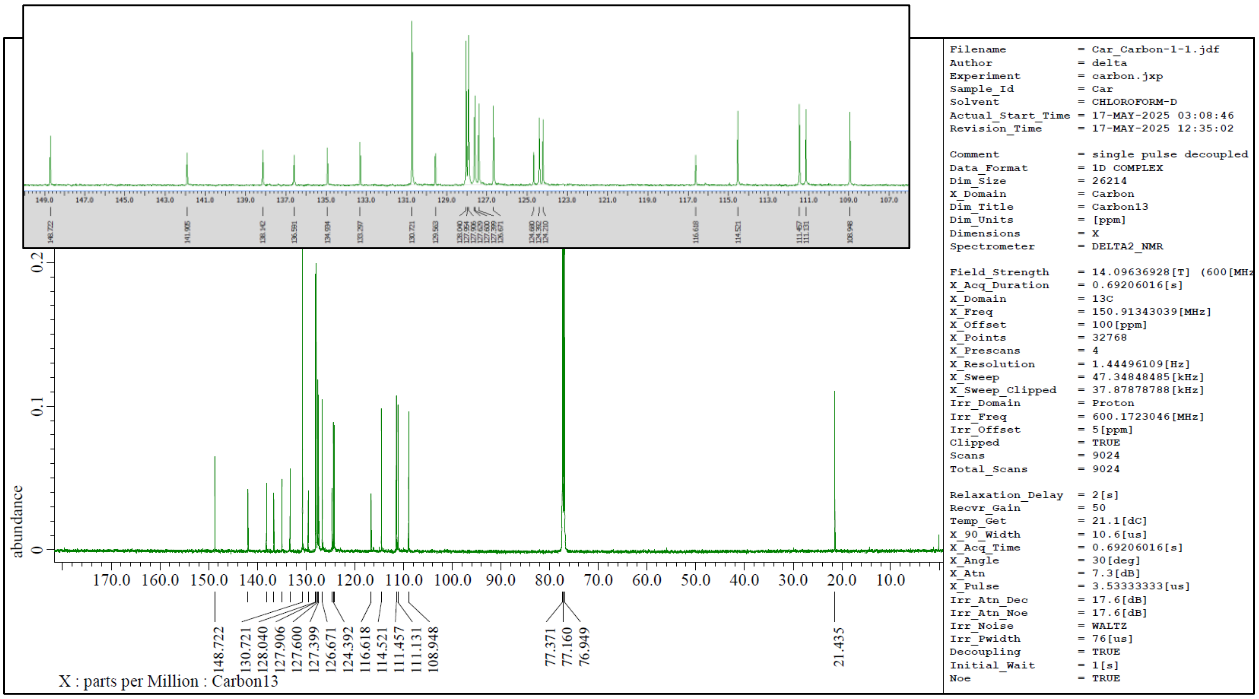

3 (^{13}C NMR, 151 MHz, $CDCl_3$).

*1.4. General procedure for the synthesis of 9-(p-tolyl)-9H-naphtho[2,1-c]carbazol-12-ol **3***

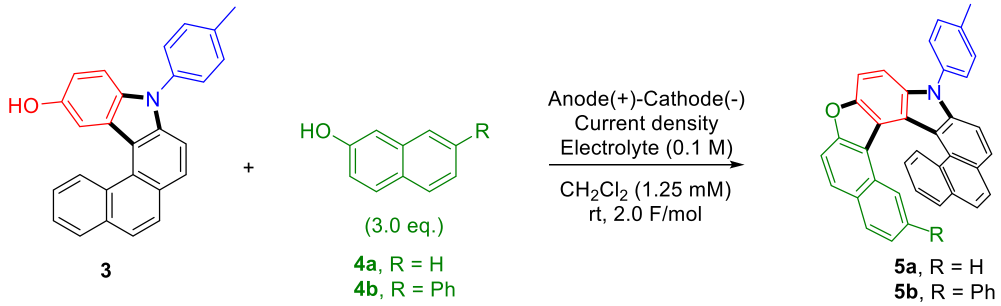

				Yield %	
**Anode(+)**	**Cathode(-)**	**Bu_4NPF_6 (0.1 M)**	**Current density**	**5a**	**5b**
Pt	Pt	Bu_4NPF_6 (0.1 M)	J = 0.75 mA/cm^2	33%	17%
Pt	C	Bu_4NPF_6 (0.1 M)	J = 0.75 mA/cm^2	47%	35%
Pt	C	$LiClO_4$ (0.01 M)	J = 0.75 mA/cm^2	61%	<5%
Pt	C	Bu_4NPF_6 (0.01 M)	J = 0.5 mA/cm^2	55%	<5%
Pt	C	Bu_4NPF_6 (0.1 M)	J = 0.25 mA/cm^2	66%	41%
Pt	C	Bu_4NPF_6 (0.1 M)	J = 0.5 mA/cm^2	62%	45%
Pt	C	Bu_4NPF_6 (0.1 M)	J = 1.0 mA/cm^2	58%	21%

A 20 mL DCM solution of **3** (0.025 mmol), β-naphthol (0.075 mmol), and *n*-tetrabutylammonium hexafluorophosphate(V) (2.0 mmol) was transferred into an undivided electrolysis cell. This cell is equipped with one Pt anode and one carbon cathode connected to a DC power supply. At room temperature, a constant current of 2.0 mA was applied. After the completion of reaction, the electrolysis was stopped and crude mixture was purified by column chromatography (SiO_2, EtOAc/*n*-hexane) to afford oxaza[8]helicenes **5** as a yellow solid.

*10-(p-Tolyl)-10H-naphtho[2,1-c]naphtho[1',2':4,5]furo[3,2-g]carbazole **5a***

^{1}H NMR (400 MHz, $CDCl_3$) δ 8.59 (d, J = 8.2 Hz, 1H), 7.98 (d, J = 8.7 Hz, 1H), 7.95 (d, J = 8.7 Hz, 1H), 7.83 (d, J = 9.2 Hz, 1H), 7.79-7.80 (m, 2H), 7.73 (d, J = 8.2 Hz, 1H), 7.68 (d, J = 8.7 Hz, 1H), 7.57-7.60 (m, 4H), 7.49-7.53 (m, 4H), 6.95-6.99 (m, 1H), 6.86-6.90 (m, 1H), 6.36-6.41 (m, 2H), 2.57 (s, 3H).

^{13}C NMR (176 MHz, $CDCl_3$) δ 153.69, 151.96, 140.90, 138.56, 138.50, 134.81, 132.13, 131.95, 130.86, 130.37, 129.39, 128.35, 128.21, 127.41, 127.36, 127.06, 127.02, 126.82, 126.79, 126.66, 125.84, 124.65, 124.08, 124.02, 123.31, 121.17, 119.02, 117.03, 116.66, 111.93, 110.77, 109.82, 108.94, 21.53 (One carbon overlapped).

HRMS (APCI): calcd for $C_{37}H_{24}NO^+$: m/z 498.1852 [M + H]+, found 498.1856.

IR (KBr): 3021, 2935, 1758, 1630, 1367, 1207, 899, 601 cm^{-1}.

mp: 241 – 242 °C.

2-Phenyl-10-(p-tolyl)-10H-naphtho[2,1-c]naphtho[1',2':4,5]furo[3,2-g]carbazole ***5b***

^{1}H NMR (600 MHz, $CDCl_3$) *δ* 8.69 (d, *J* = 8.2 Hz, 1H), 7.92 (d, *J* = 1.7 Hz, 1H), 7.86 (d, *J* = 8.9 Hz, 1H), 7.82 (d, *J* = 8.2 Hz, 2H), 7.78 (d, *J* = 8.9 Hz, 1H), 7.52-7.67 (m, 7H), 7.42 (d, *J* = 8.9 Hz, 1H), 7.38 (d, *J* = 7.6 Hz, 1H), 7.28 (dd, *J* = 8.2, 2.1 Hz, 1H), 7.24-7.26 (m, 1H), 7.17 (t, *J* = 7.6 Hz, 2H), 7.04 (d, *J* = 8.2 Hz, 1H), 6.88 (t, *J* = 6.9 Hz, 1H), 6.82-6.83 (m, 2H), 6.40-6.43 (m, 1H), 2.57 (s, 3H).

^{13}C NMR (151 MHz, $CDCl_3$) *δ* 154.04, 152.04, 140.83, 140.49, 138.65, 138.59, 136.61, 134.87, 131.74, 131.54, 130.92, 130.86, 128.61, 128.46, 128.26, 128.05, 127.86, 127.80, 127.47, 127.42, 127.18, 127.13, 126.81, 126.61, 125.71, 125.57, 125.55, 124.76, 123.73, 122.55, 121.79, 119.14, 117.21, 116.81, 111.98, 110.80, 109.79, 109.07, 21.52.

HRMS (APCI): calcd for $C_{43}H_{28}NO^+$: m/z 574.2165 [M + H]+, found 574.2163.

IR (KBr): 2953, 2927, 1658, 1581, 1514, 1084, 795, 753 cm^{-1}.

mp: >300 °C.

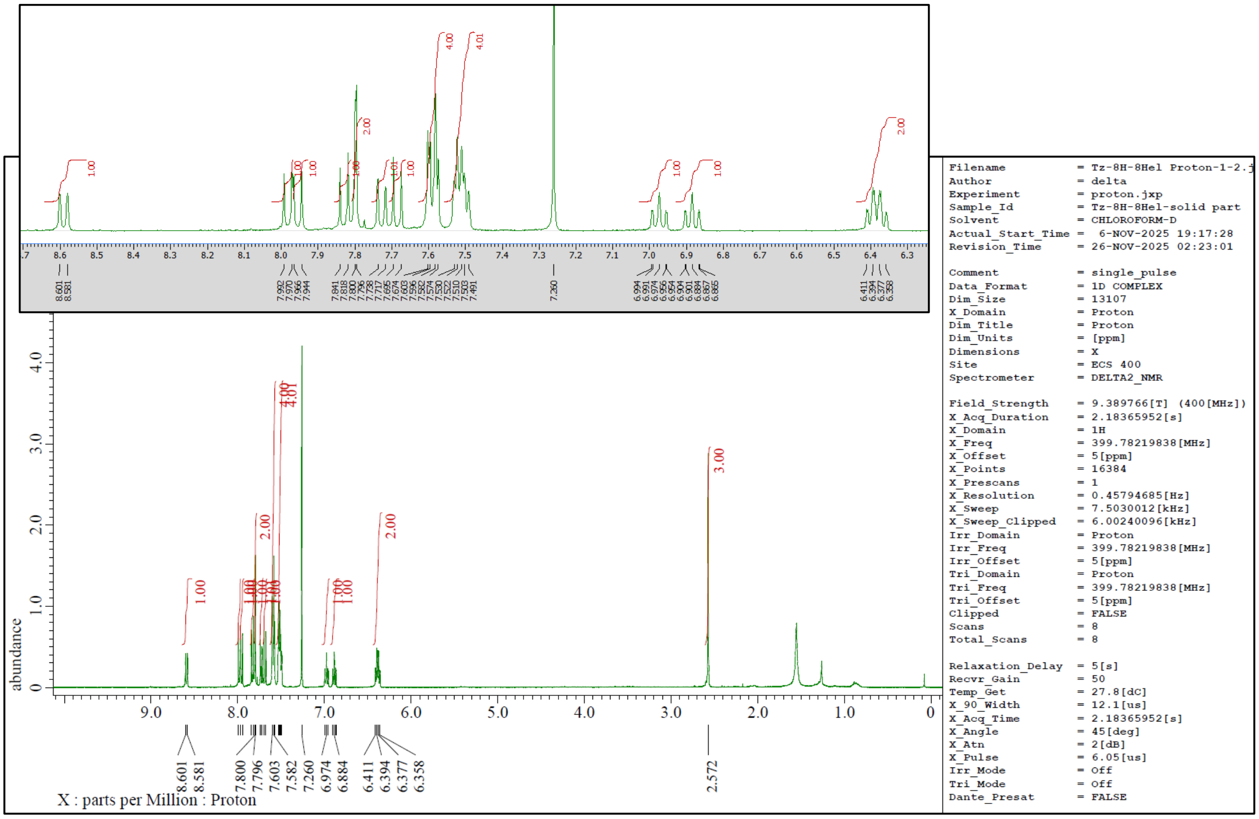

5a (^{1}H NMR, 400 MHz, $CDCl_3$).

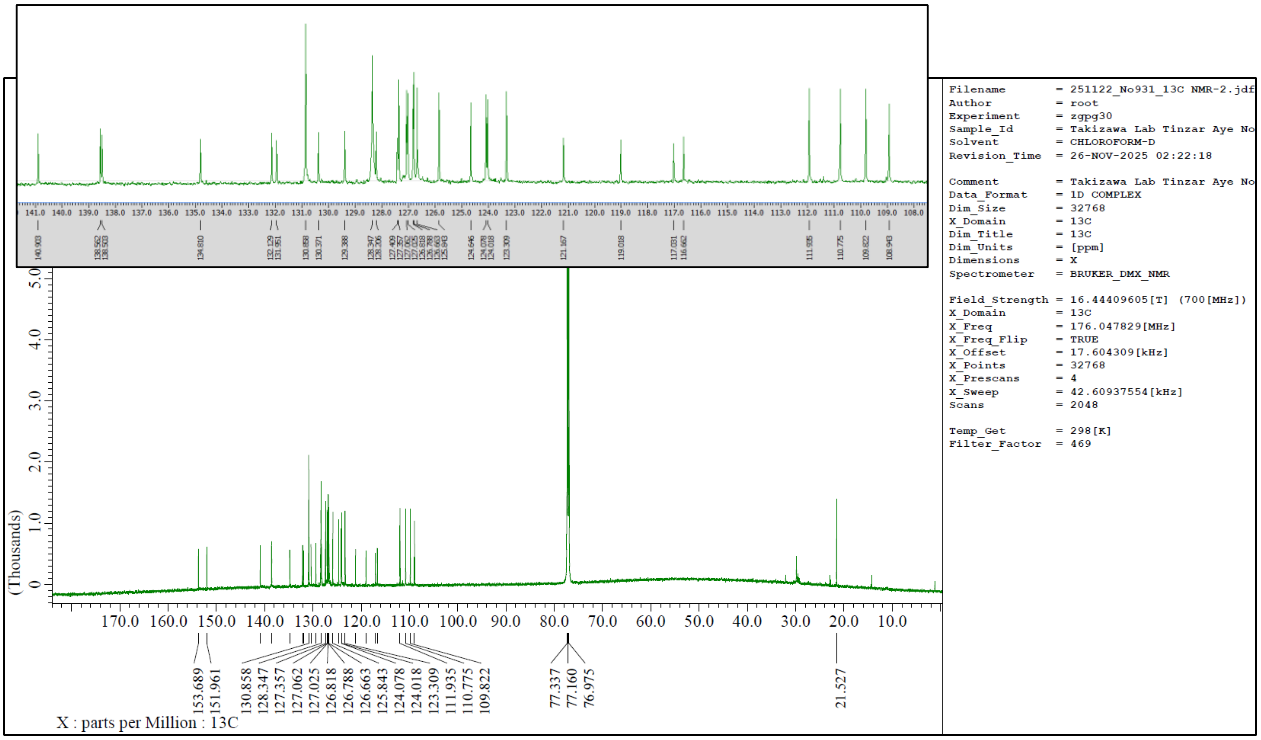

5a (^{13}C NMR, 176 MHz, $CDCl_3$).

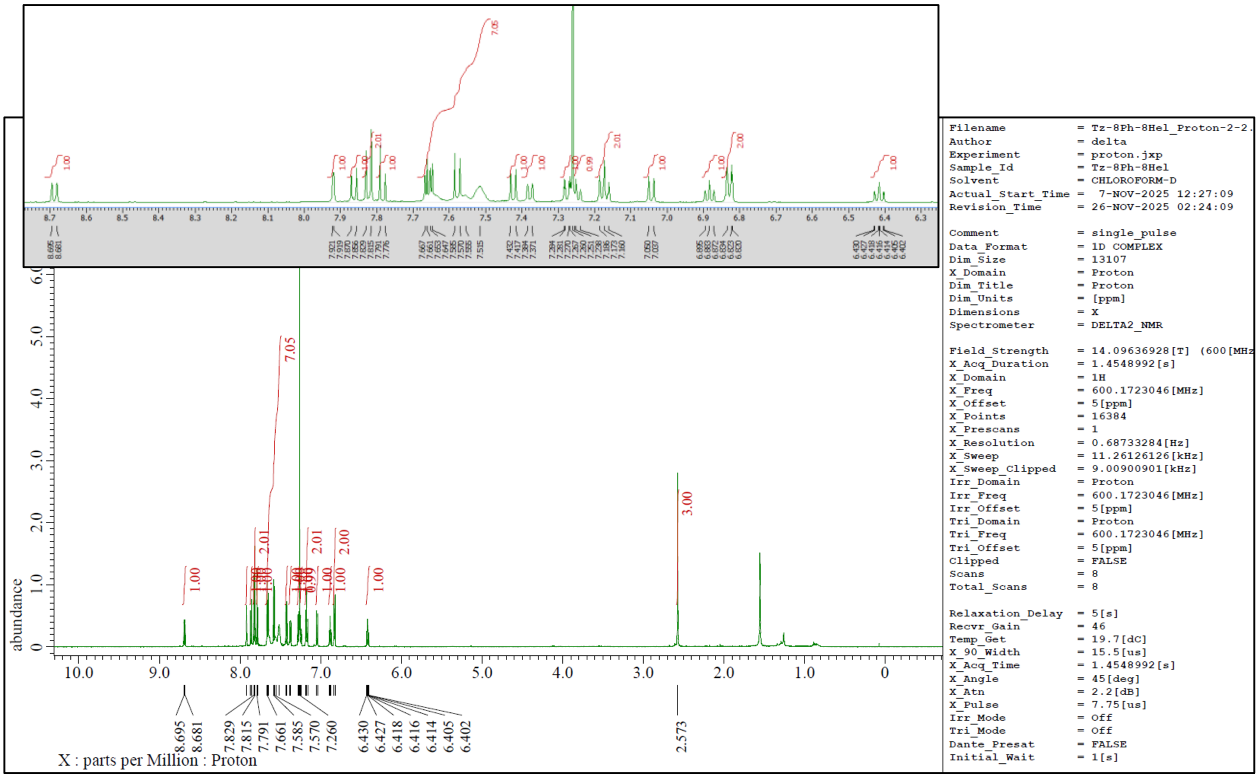

5b (^{1}H NMR, 600 MHz, $CDCl_3$).

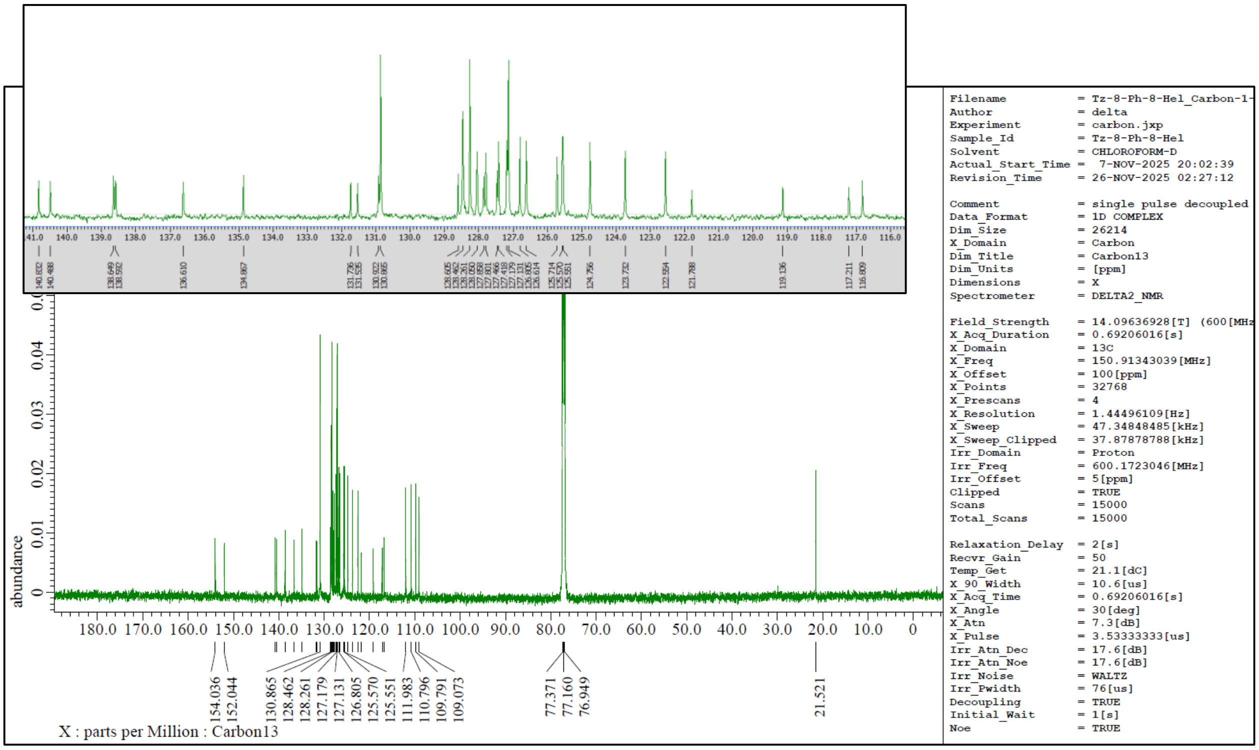

5b (^{13}C NMR, 151 MHz, $CDCl_3$).

2. *HPLC Chiral resolution of compounds* ***5a,b*** *&* ***6a,b***

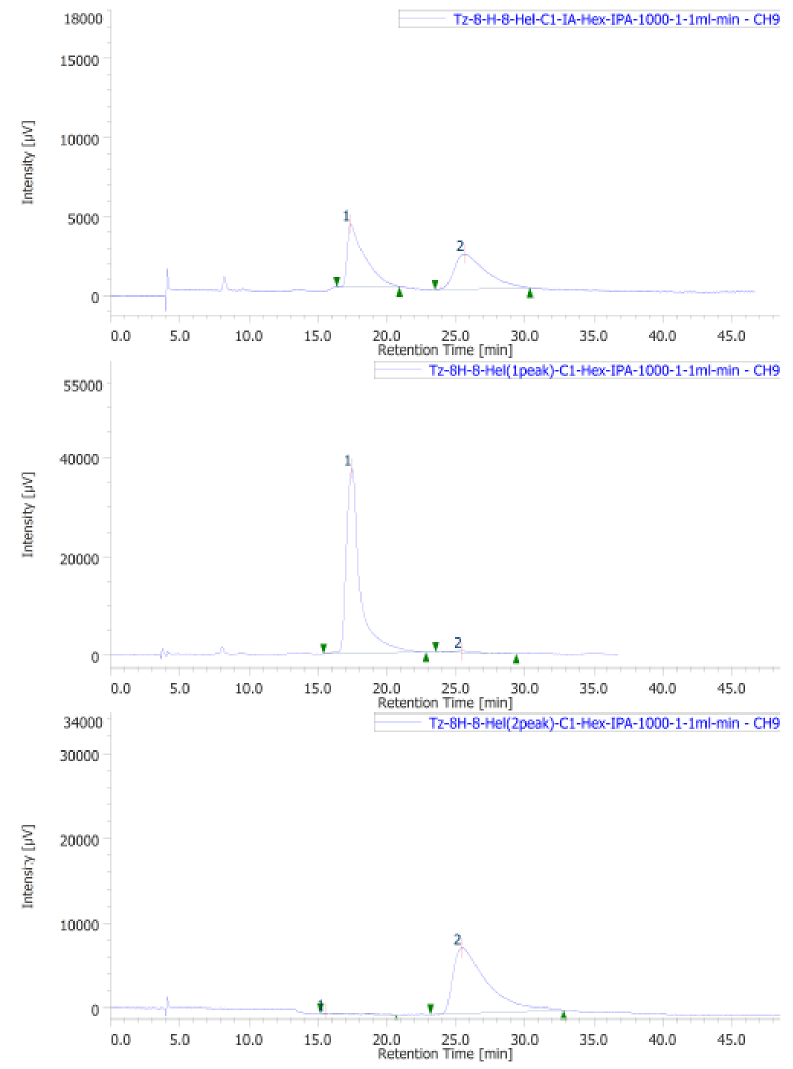

Channel & Peak Information Table
Chromatogram Name Tz-8-H-8-Hel-C1-IA-Hex-IPA-1000-1-1ml-min-CH9
Sample Name
Channel Name 295.0nm

#	Peak Name	CH	tR [min]	Area [µV·sec]	Height [µV]	Area%	Height%	Quantity	NTP	Resolution	Symmetry Factor	Warning
1	Unknown	9	17.380	348154	3985	49.707	63.990	N/A	1100	2.686	3.369	
2	Unknown	9	25.647	352259	2243	50.293	36.010	N/A	634	N/A	1.723	

Chromatogram Name Tz-8H-8-Hel(1peak)-C1-Hex-IPA-1000-1-1ml-min-CH9
Sample Name
Channel Name 295.0nm

#	Peak Name	CH	tR [min]	Area [µV·sec]	Height [µV]	Area%	Height%	Quantity	NTP	Resolution	Symmetry Factor	Warning
1	Unknown	9	17.487	2589978	37306	98.511	99.052	N/A	2237	3.535	2.117	
2	Unknown	9	25.467	39148	357	1.489	0.948	N/A	1117	N/A	1.682	

Chromatogram Name Tz-8H-8-Hel(2peak)-C1-Hex-IPA-1000-1-1ml-min-CH9
Sample Name
Channel Name 295.0nm

#	Peak Name	CH	tR [min]	Area [µV·sec]	Height [µV]	Area%	Height%	Quantity	NTP	Resolution	Symmetry Factor	Warning
1	Unknown	9	15.547	3549	17	0.270	0.220	N/A	48	1.515	11.192	
2	Unknown	9	25.427	1310048	7710	99.730	99.780	N/A	611	N/A	3.006	

HPLC (Chiral analytical IA column), *n*-Hexane / iPrOH = 1000/1, Flow rate: 1.0 mL/min, λ = 295 nm, t_1 (*P*)-**5a** = 17.3 min, t_2 (*M*)-**5a** = 25.6 min.

HPLC (Chiral semi-preparative IA column), *n*-Hexane / iPrOH = 1000/1, Flow rate: 5.0 mL/min, λ = 295 nm, t_1 (*P*)-**5a** = 69.2 min, t_2 (*M*)-**5a** = 98.3 min.

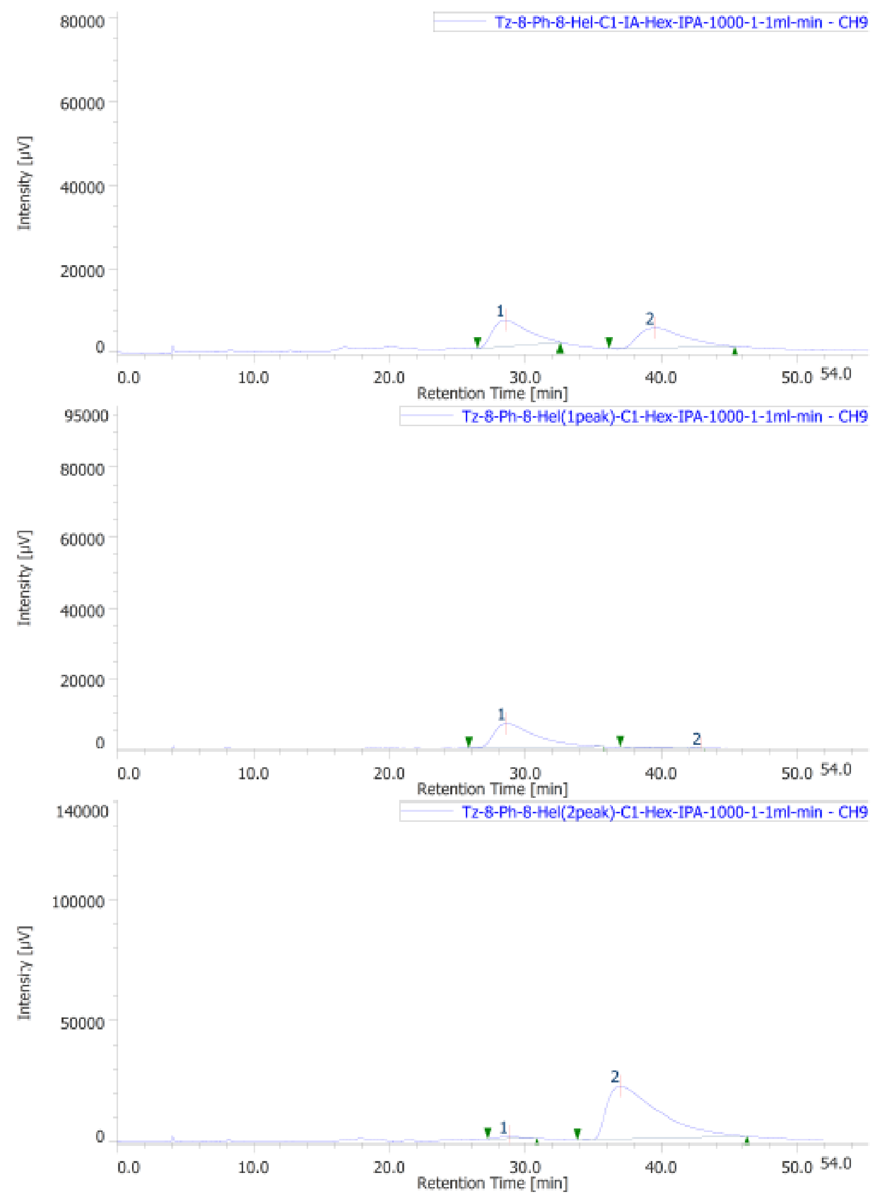

Channel & Peak Information Table

Chromatogram Name Tz−8−Ph−8−Hel−C1−IA−Hex−IPA−1000−1−1ml−min−CH9
Sample Name
Channel Name 270.0nm

#	Peak Name	CH	tR [min]	Area [µV·sec]	Height [µV]	Area%	Height%	Quantity	NTP	Resolution	Symmetry Factor	Warning
1	Unknown	9	28.513	1107975	6302	50.155	57.175	N/A	575	2.007	1.587	
2	Unknown	9	39.527	1101115	4720	49.845	42.825	N/A	640	N/A	1.721	

Chromatogram Name Tz−8−Ph−8−Hel(1peak)−C1−Hex−IPA−1000−1−1ml−min−CH9
Sample Name
Channel Name 270.0nm

#	Peak Name	CH	tR [min]	Area [µV·sec]	Height [µV]	Area%	Height%	Quantity	NTP	Resolution	Symmetry Factor	Warning
1	Unknown	9	28.593	1465719	6915	98.841	99.817	N/A	462	1.851	2.205	
2	Unknown	9	42.967	17194	13	1.159	0.183	N/A	281	N/A	0.516	

Chromatogram Name Tz−8−Ph−8−Hel(2peak)−C1−Hex−IPA−1000−1−1ml−min−CH9
Sample Name
Channel Name 270.0nm

#	Peak Name	CH	tR [min]	Area [µV·sec]	Height [µV]	Area%	Height%	Quantity	NTP	Resolution	Symmetry Factor	Warning
1	Unknown	9	28.743	107931	863	1.833	3.800	N/A	988	1.570	1.181	
2	Unknown	9	36.953	5779217	21838	98.167	96.200	N/A	469	N/A	2.805	

HPLC (Chiral analytical IA column), *n*-Hexane / iPrOH = 1000/1, Flow rate: 1.0 mL/min, λ = 270 nm, t_1 (*P*)-**5b** = 28.5 min, t_2 (*M*)-**5b** = 39.5 min.

HPLC (Chiral semi-preparative IA column), *n*-Hexane / iPrOH = 1000/1, Flow rate: 5.0 mL/min, λ = 270 nm, t_1 (*P*)-**5b** = 98.2 min, t_2 (*M*)-**5b** = 124.2 min.

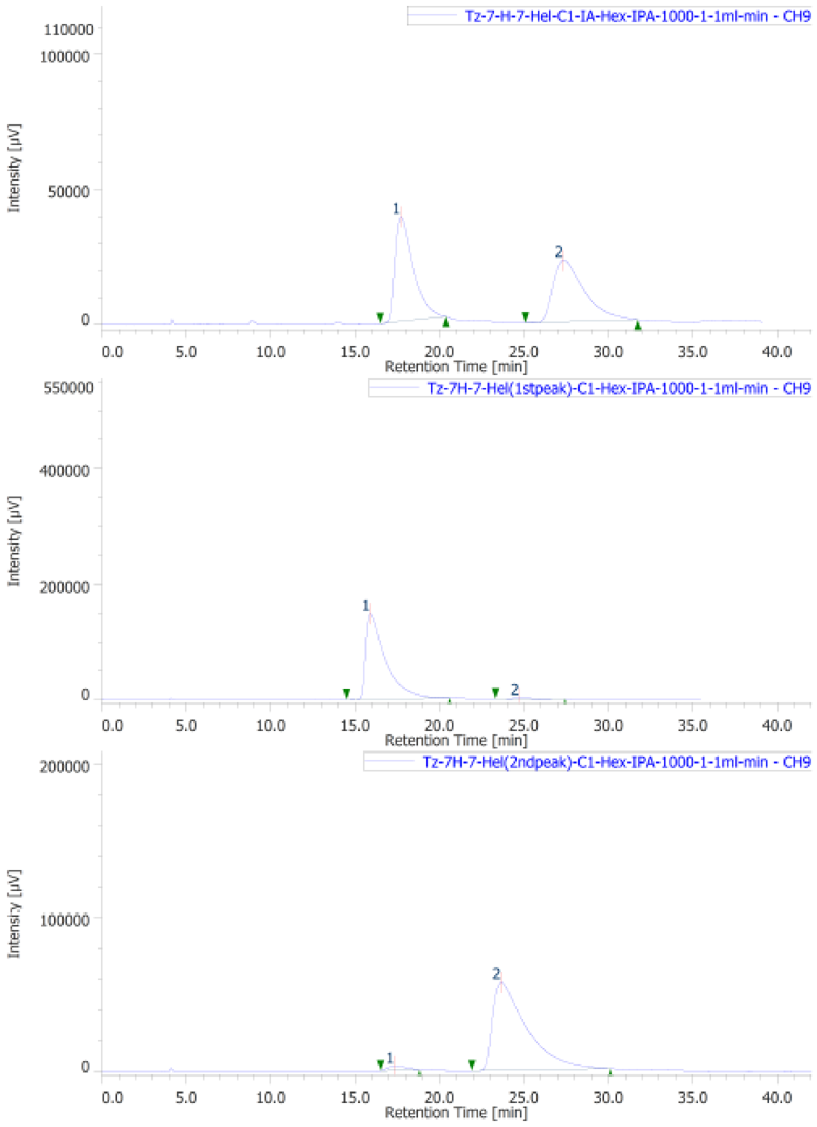

Channel & Peak Information Table

Chromatogram Name Tz−7−H−7−Hel−C1−IA−Hex−IPA−1000−1−1ml−min−CH9
Sample Name
Channel Name 270.0nm

#	Peak Name	CH	tR [min]	Area [µV·sec]	Height [µV]	Area%	Height%	Quantity	NTP	Resolution	Symmetry Factor	Warning
1	Unknown	9	17.703	2874740	38579	49.534	62.985	N/A	1412	3.743	2.027	
2	Unknown	9	27.340	2928772	22672	50.466	37.015	N/A	1113	N/A	1.923	

Chromatogram Name Tz−7H−7−Hel(1stpeak)−C1−Hex−IPA−1000−1−1ml−min−CH9
Sample Name
Channel Name 270.0nm

#	Peak Name	CH	tR [min]	Area [µV·sec]	Height [µV]	Area%	Height%	Quantity	NTP	Resolution	Symmetry Factor	Warning
1	Unknown	9	15.900	12045906	148389	98.981	99.263	N/A	1086	3.577	3.060	
2	Unknown	9	24.720	124011	1101	1.019	0.737	N/A	1076	N/A	1.613	

Chromatogram Name Tz−7H−7−Hel(2ndpeak)−C1−Hex−IPA−1000−1−1ml−min−CH9
Sample Name
Channel Name 270.0nm

#	Peak Name	CH	tR [min]	Area [µV·sec]	Height [µV]	Area%	Height%	Quantity	NTP	Resolution	Symmetry Factor	Warning
1	Unknown	9	17.340	195402	2579	2.376	4.317	N/A	1018	2.257	1.378	
2	Unknown	9	23.643	8029908	57144	97.624	95.683	N/A	762	N/A	2.886	

HPLC (Chiral analytical IA column), *n*-Hexane / iPrOH = 1000/1, Flow rate: 1.0 mL/min, λ = 270 nm, t_1 (*P*)-**6a** = 17.7 min, t_2 (*M*)-**6a** = 27.3 min.

HPLC (Chiral semi-preparative IA column), *n*-Hexane / iPrOH = 1000/1, Flow rate: 5.0 mL/min, λ = 270 nm, t_1 (*P*)-**6a** = 59.3 min, t_2 (*M*)-**6a** = 95.1 min.

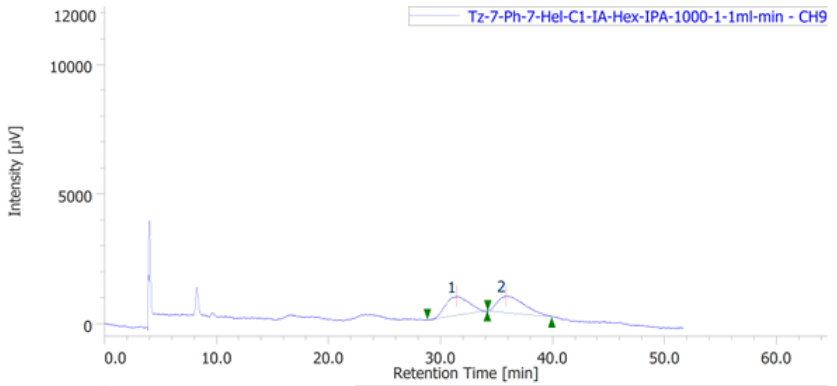

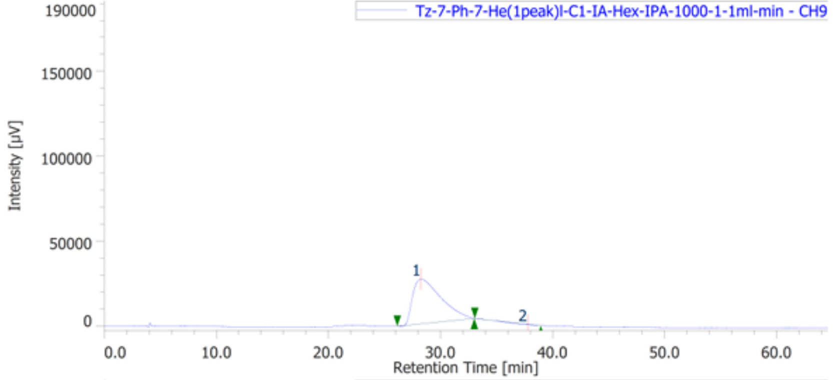

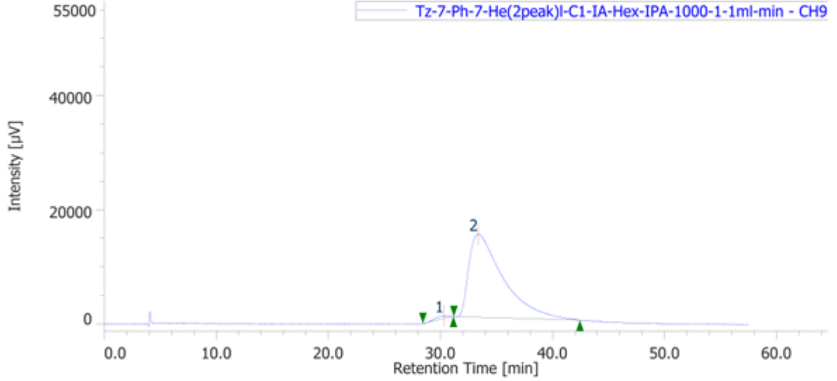

Channel & Peak Information Table

Chromatogram Name Tz-7-Ph-7-Hel-C1-IA-Hex-IPA-1000-1-1ml-min-CH9
Sample Name
Channel Name 290.0nm

#	Peak Name	CH	tR [min]	Area [µV·sec]	Height [µV]	Area%	Height%	Quantity	NTP	Resolution	Symmetry Factor	Warning
1	Unknown	9	31.430	103595	732	49.126	52.802	N/A	935	1.039	1.114	
2	Unknown	9	35.863	107281	654	50.874	47.198	N/A	1042	N/A	1.867	

Chromatogram Name Tz-7-Ph-7-He(1peak)l-C1-IA-Hex-IPA-1000-1-1ml-min-CH9
Sample Name
Channel Name 290.0nm

#	Peak Name	CH	tR [min]	Area [µV·sec]	Height [µV]	Area%	Height%	Quantity	NTP	Resolution	Symmetry Factor	Warning
1	Unknown	9	28.270	4621428	26352	98.534	98.731	N/A	572	1.947	2.098	
2	Unknown	9	37.760	68780	339	1.466	1.269	N/A	895	N/A	0.622	

Chromatogram Name Tz-7-Ph-7-He(2peak)l-C1-IA-Hex-IPA-1000-1-1ml-min-CH9
Sample Name
Channel Name 290.0nm

#	Peak Name	CH	tR [min]	Area [µV·sec]	Height [µV]	Area%	Height%	Quantity	NTP	Resolution	Symmetry Factor	Warning
1	Unknown	9	30.333	43238	506	1.380	3.374	N/A	2413	0.793	0.779	
2	Unknown	9	33.377	3090288	14497	98.620	96.626	N/A	652	N/A	2.640	

HPLC (Chiral analytical IA column), *n*-Hexane / iPrOH = 1000/1, Flow rate: 1.0 mL/min, λ = 290 nm, t_1 (*P*)-**6b** = 31.4 min, t_2 (*M*)-**6b** = 35.8 min.

HPLC (Chiral semi-preparative IA column), *n*-Hexane / iPrOH = 1000/1, Flow rate: 5.0 mL/min, λ = 290 nm, t_1 (*P*)-**6b** = 112.2 min, t_2 (*M*)-**6b** = 131.3 min.

3. *DFT and TD-DFT calculations to study the optoelectronic features*

All DFT calculations were performed using the Gaussian 16 and Gaussian 9 packages of programs. The geometries of the structures of oxaza[7]helicenes **6** and oxaza[8]helicenes **5** were optimized at both the ground S_0 and excited S_1 states, calculated with the MN15/6-311G (2d,p)/SMD=chloroform level of theory. All stationary points were identified as stable minima by frequency calculations, and the geometry optimization was achieved using the standard criteria in Gaussian software. The nucleus-independent chemical shift (NICS) indices were calculated at the center of each ring ($NICS_{(0)}$), 1 Å and 2 Å above/below the center ($NICS_{(1,2)}$) within the gauge-independent atomic orbital (GIAO) approximation at MN15/6-311G (2d,p)/SMD=chloroform level of theory and visualized using py.Aroma: an intuitive graphical user interface for diverse aromaticity analyses. For the anisotropy of the induced current density (AICD) simulations, the AICD-3.0.4 software was used. TD-DFT calculations were performed on optimized structures at the lowest energy singlet excited state (S_1).

*3.1. Molecular orbitals of **5** and **6***

Table S1. Selected molecular orbitals of **5a** optimized in the ground state S_0 calculated at MN15/6-311G (2d,p)/SMD=chloroform level of theory (isosurface value = 0.02 a.u.).

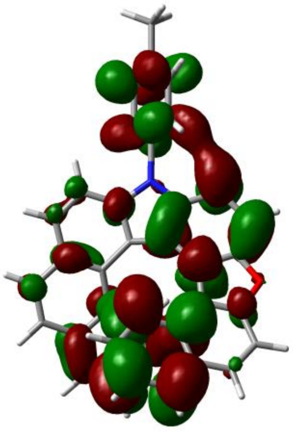<br>LUMO+3 (−0.122 eV)	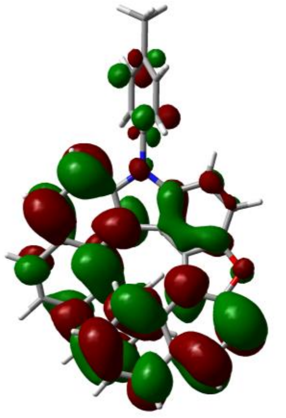<br>LUMO+2 (−0.2667 eV)	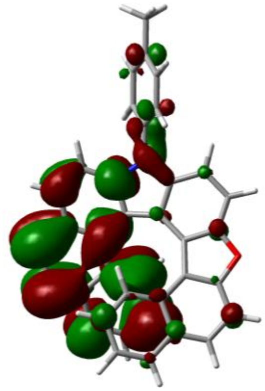<br>LUMO+1 (−0.426 eV)
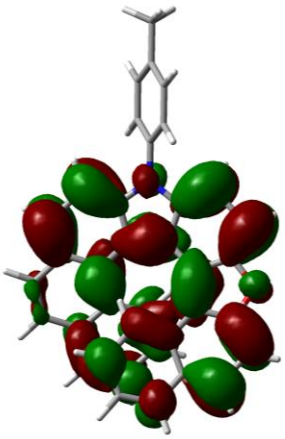<br>LUMO (−1.157 eV)	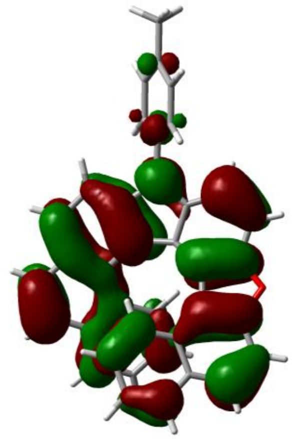<br>HOMO (−5.886 eV)	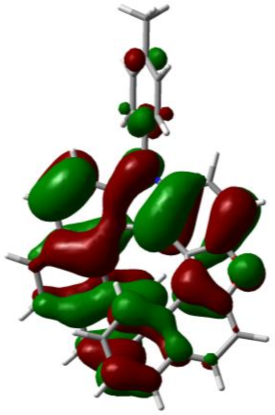<br>HOMO−1 (−6.249 eV)
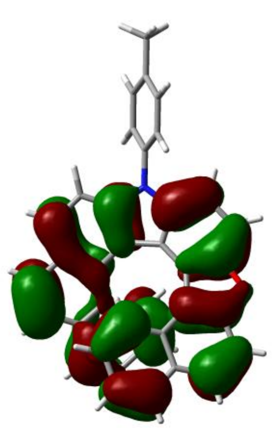<br>HOMO−2 (−6.693 eV)	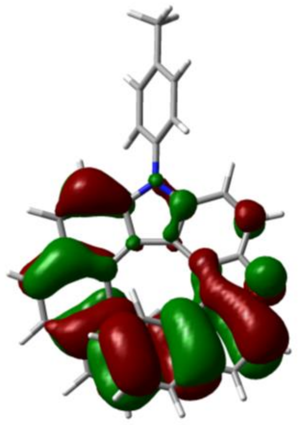<br>HOMO−3 (−7.168 eV)	

Table S2. Selected molecular orbitals of **5a** optimized in the excited state S_1 calculated at MN15/6-311G (2d,p)/SMD=chloroform level of theory (isosurface value = 0.02 a.u.).

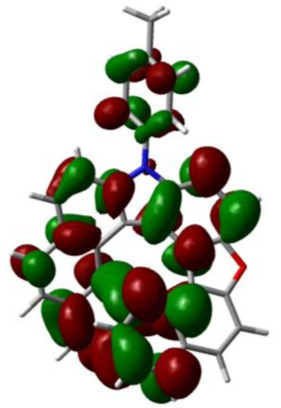<br>LUMO+3 (0.069 eV)	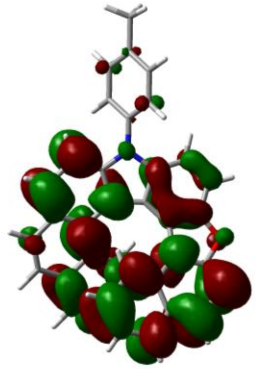<br>LUMO+2 (−0.336 eV)	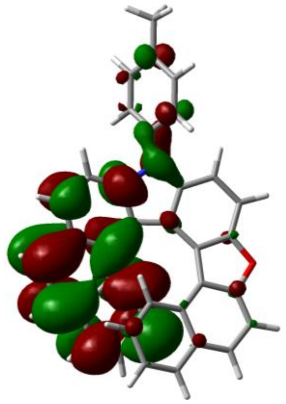<br>LUMO+1 (−0.477 eV)
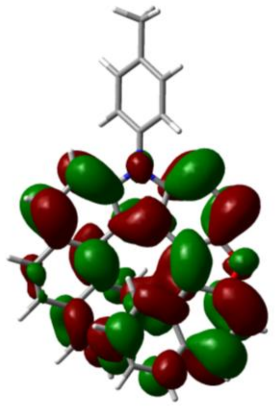<br>LUMO (−1.437 eV)	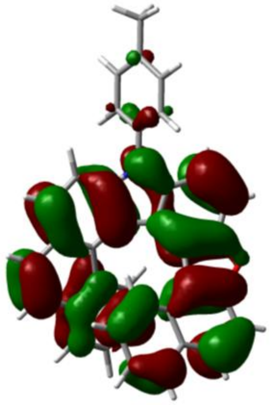<br>HOMO (−5.662 eV)	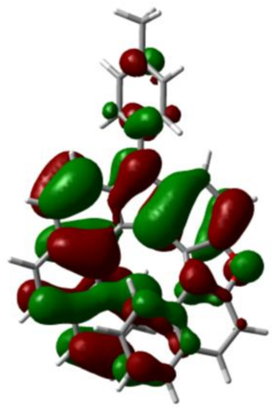<br>HOMO−1 (−6.238 eV)
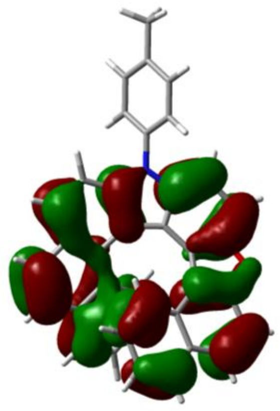<br>HOMO−2 (−6.676 eV)	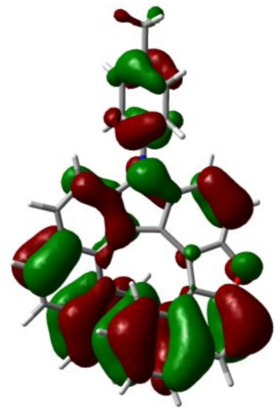<br>HOMO−3 (−7.131 eV)	

Table S3. Selected molecular orbitals of **5b** optimized in the ground state S_0 calculated at MN15/6-311G (2d,p)/SMD=chloroform level of theory (isosurface value = 0.02 a.u.).

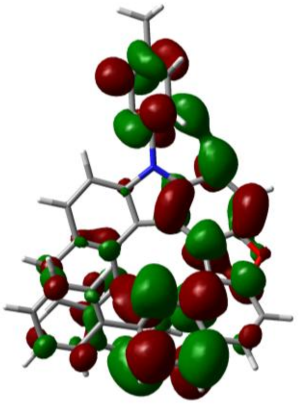 LUMO+3 (0.071 eV)	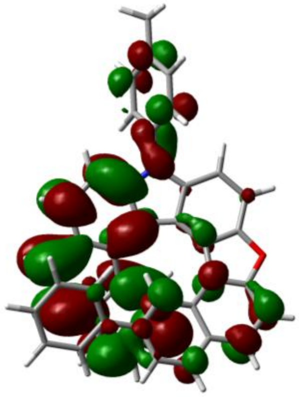 LUMO+2 (−0.366 eV)	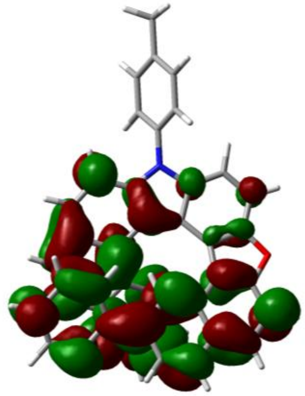 LUMO+1 (−0.526 eV)
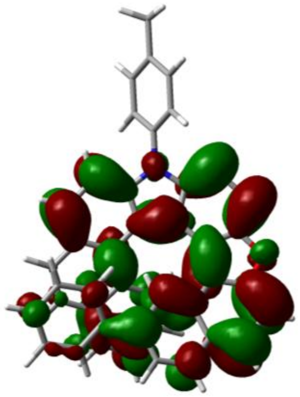 LUMO (−1.204 eV)	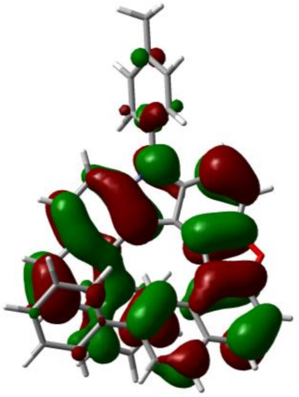 HOMO (−5.858 eV)	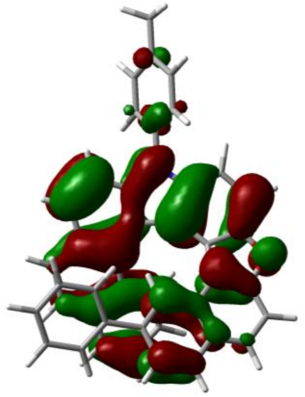 HOMO−1 (−6.243 eV)
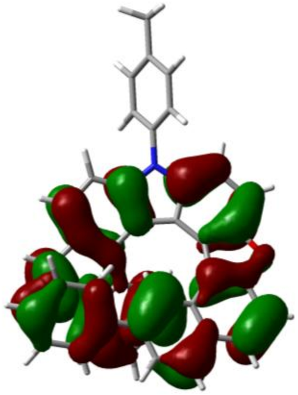 HOMO−2 (−6.606 eV)	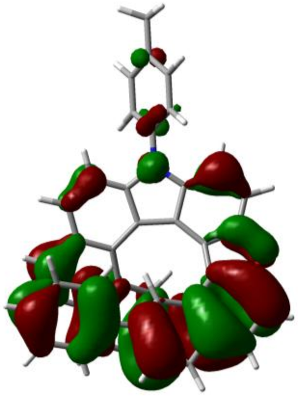 HOMO−3 (−6.961 eV)	

Table S4. Selected molecular orbitals of **5b** optimized in the excited state S_1 calculated at MN15/6-311G (2d,p)/SMD=chloroform level of theory (isosurface value = 0.02 a.u.).

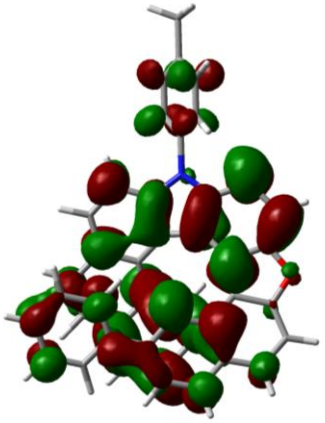<br>LUMO+3 (−0.002 eV)	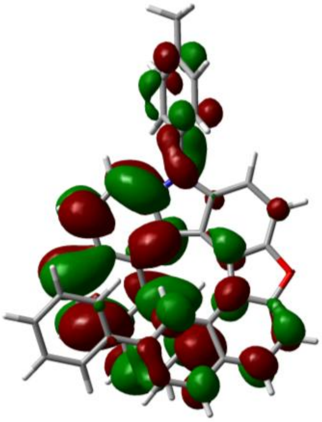<br>LUMO+2 (−0.437 eV)	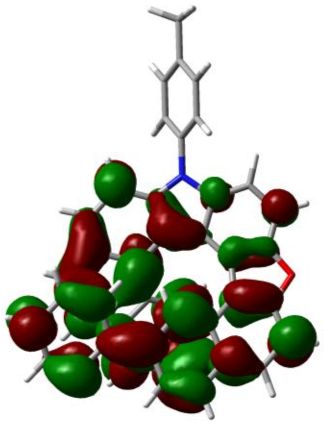<br>LUMO+1 (−0.581 eV)
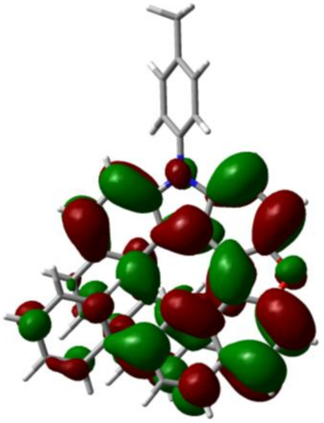<br>LUMO (−1.509 eV)	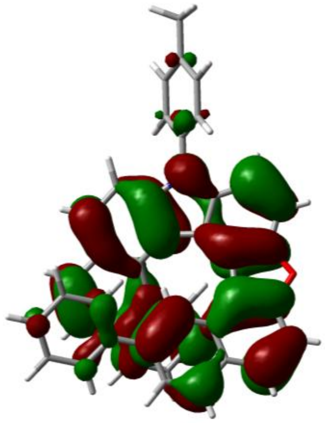<br>HOMO (−5.597 eV)	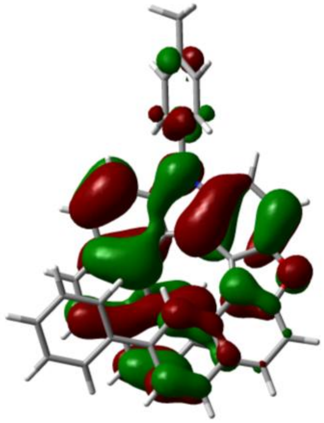<br>HOMO−1 (−6.228 eV)
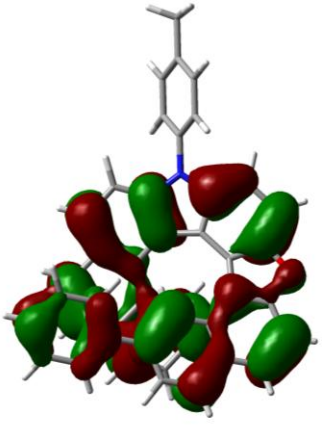<br>HOMO−2 (−6.588 eV)	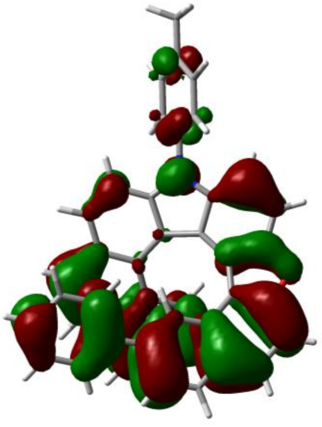<br>HOMO−3 (−6.887 eV)	

Table S5. Selected molecular orbitals of **6a** optimized in the ground state S_0 calculated at MN15/6-311G (2d,p)/SMD=chloroform level of theory (isosurface value = 0.02 a.u.).

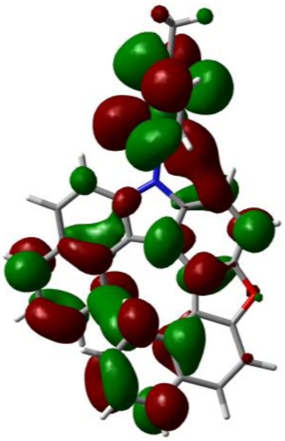	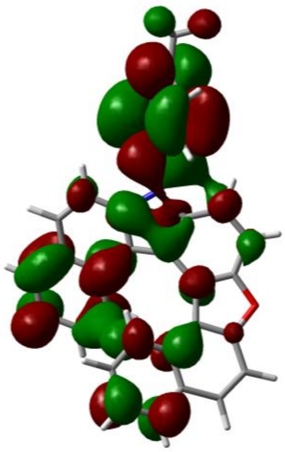	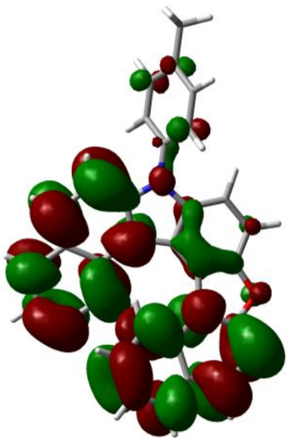
LUMO+3 (0.122 eV)	LUMO+2 (0.104 eV)	LUMO+1 (−0.276 eV)
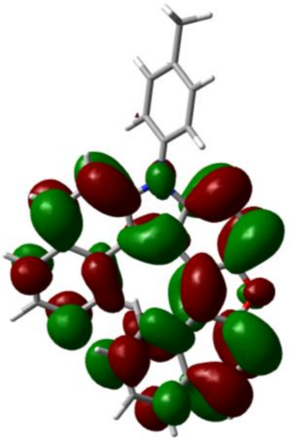	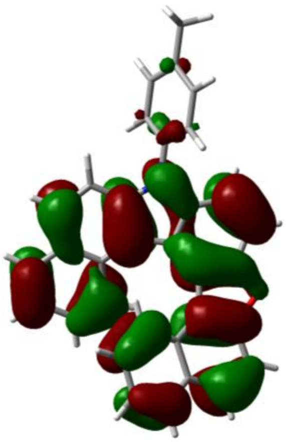	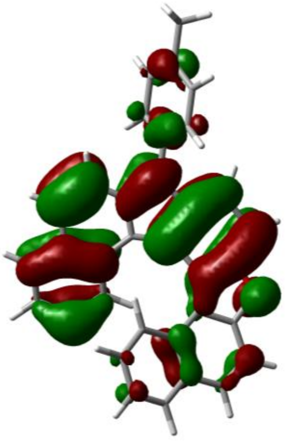
LUMO (−1.076 eV)	HOMO (−5.911 eV)	HOMO−1 (−6.343 eV)
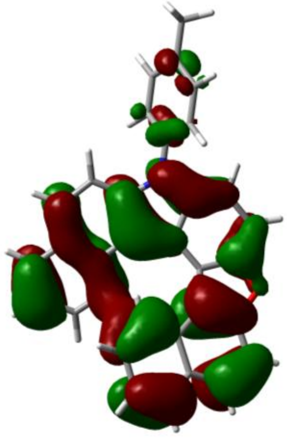	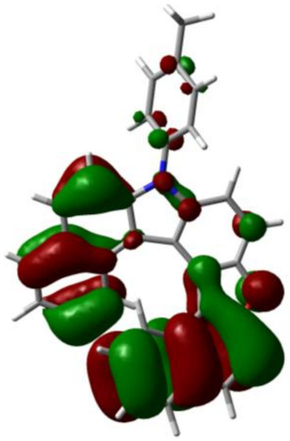	
HOMO−2 (−6.867 eV)	HOMO−3 (−7.277 eV)	

Table S6. Selected molecular orbitals of **6a** optimized in the excited state S_1 calculated at MN15/6-311G (2d,p)/SMD=chloroform level of theory (isosurface value = 0.02 a.u.).

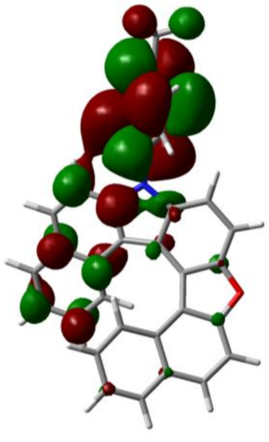<br>LUMO+3 (0.111 eV)	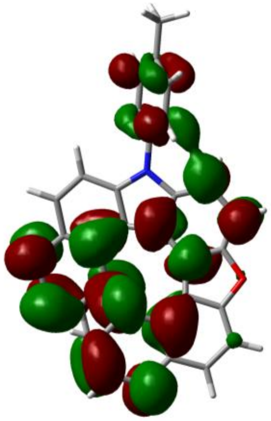<br>LUMO+2 (0.064 eV)	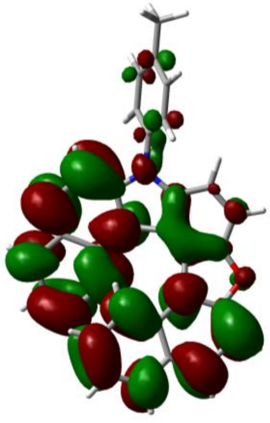<br>LUMO+1 (−0.374 eV)
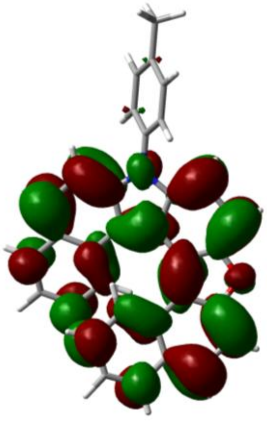<br>LUMO (−1.390 eV)	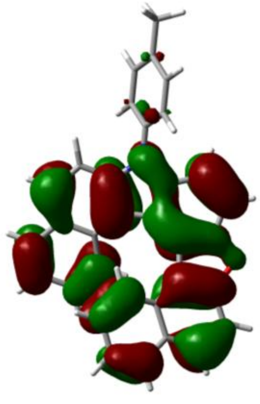<br>HOMO (−5.638 eV)	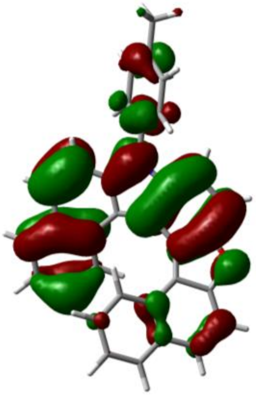<br>HOMO−1 (−6.404 eV)
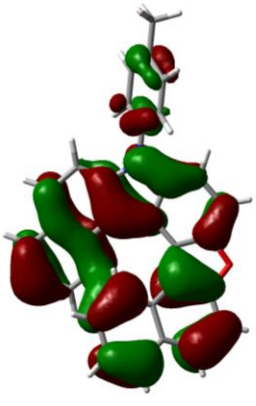<br>HOMO−2 (−6.836 eV)	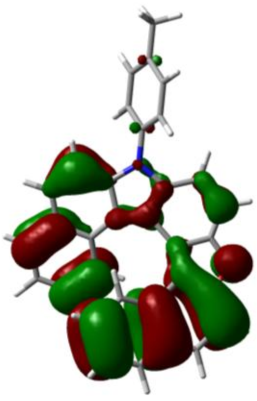<br>HOMO−3 (−7.289 eV)	

Table S7. Selected molecular orbitals of **6b** optimized in the ground state S_0 calculated at MN15/6-311G (2d,p)/SMD=chloroform level of theory (isosurface value = 0.02 a.u.).

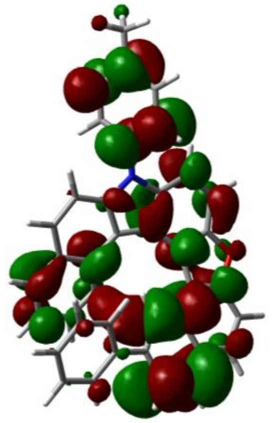<br>LUMO+3 (0.060 eV)	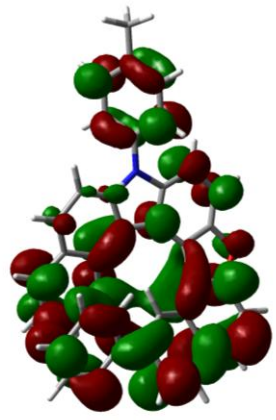<br>LUMO+2 (0.008 eV)	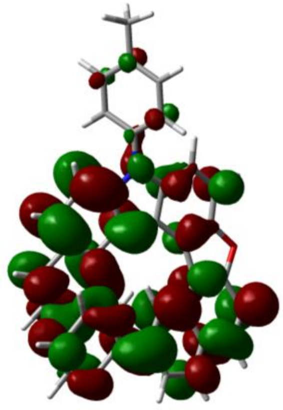<br>LUMO+1 (−0.411 eV)
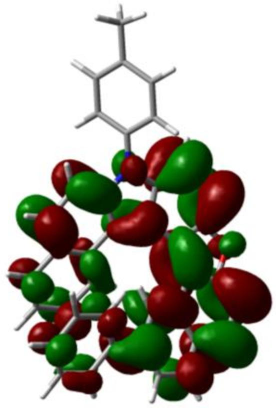<br>LUMO (−1.153 eV)	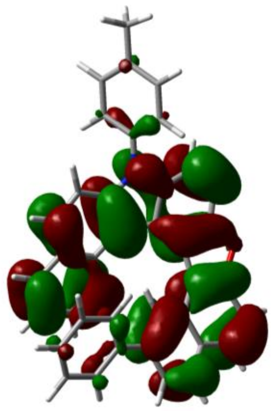<br>HOMO (−5.863 eV)	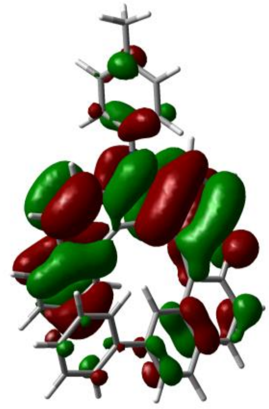<br>HOMO−1 (−6.310 eV)
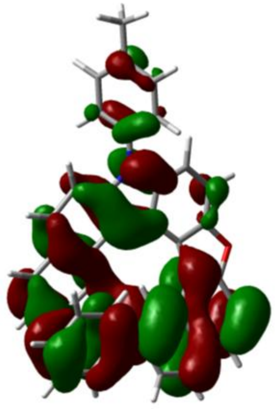<br>HOMO−2 (−6.753 eV)	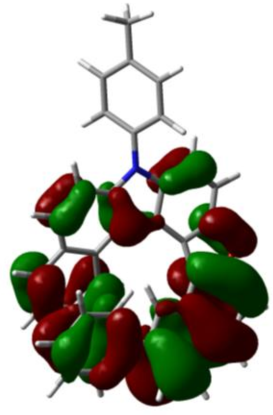<br>HOMO−3 (−7.036 eV)	

Table S8. Selected molecular orbitals of **6b** optimized in the excited state S_1 calculated at MN15/6-311G (2d,p)/SMD=chloroform level of theory (isosurface value = 0.02 a.u.).

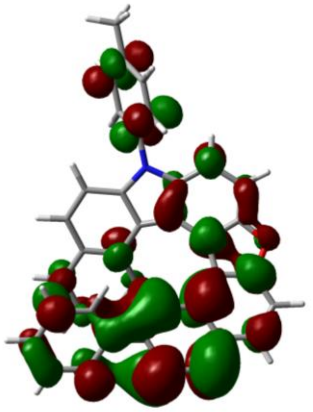 LUMO+3 (0.033 eV)	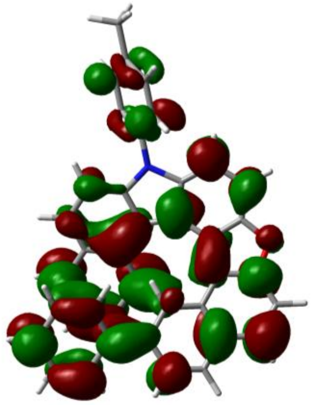 LUMO+2 (−0.086 eV)	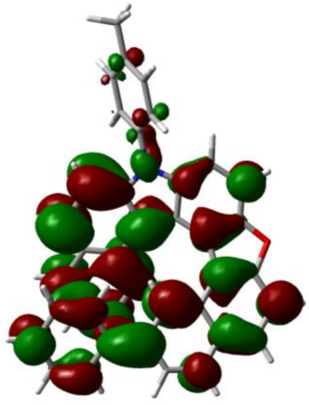 LUMO+1 (−0.528 eV)
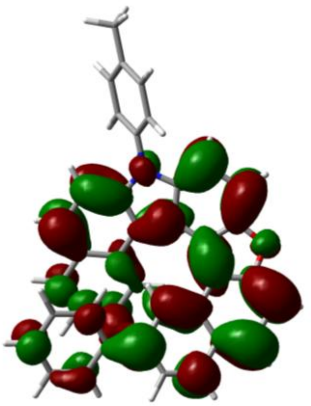 LUMO (−1.497 eV)	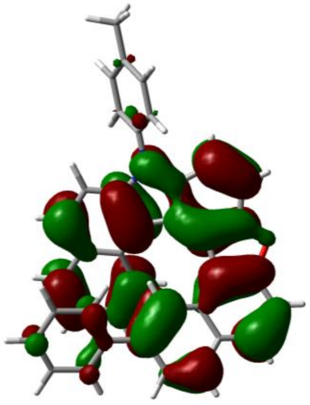 HOMO (−5.594 eV)	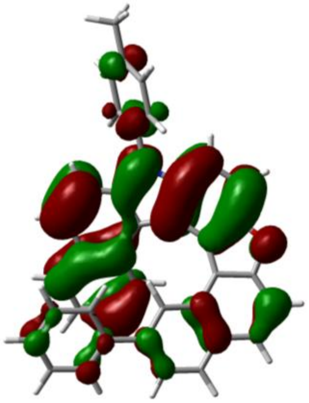 HOMO−1 (−6.362 eV)
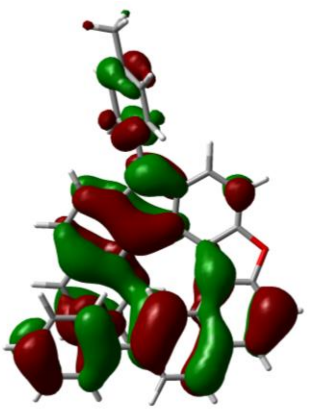 HOMO−2 (−6.710 eV)	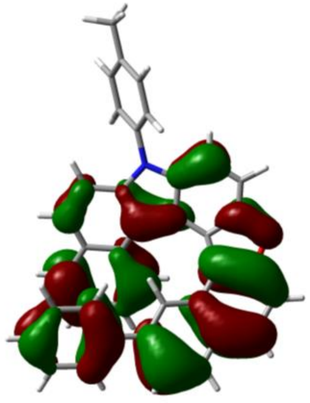 HOMO−3 (−7.035 eV)	

3.2. *Aromaticity of oxaza[8]helicenes **5a** and **5b** and oxaza[7]helicenes **6a** and **6b***

<u>Aromaticity of oxaza[8]helicene **5a**</u>

calculated at MN15/6-311G(2d,p)/SMD=chloroform level of theory

NICS(r)iso and NICS(r)zz of oxaza[8]helicene **5a**

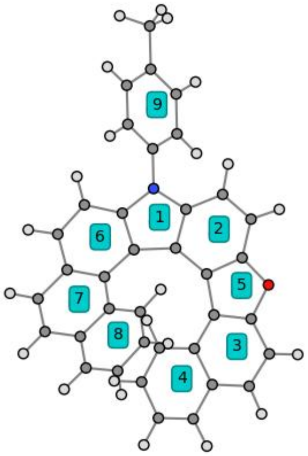

Ring	NICS(0)iso	NICS(0)zz	NICS(1)iso	NICS(1)zz	NICS(−1)iso	NICS(−1)zz	NICS(2)iso	NICS(2)zz	NICS(−2)iso	NICS(−2)zz
1	−7.6807	−3.2454	−8.2843	−21.3718	−8.1334	−20.6912	−3.8317	−13.9206	−3.7174	−13.4315
2	−8.6859	−9.2086	−10.2550	−23.2268	−9.5810	−24.5487	−4.9516	−14.2120	−4.3824	−15.9015
3	−7.7115	−6.2145	−10.8719	−19.3004	−9.0484	−17.5548	−5.8426	−12.7225	−4.2442	−9.2955
4	−8.7492	−13.7272	−12.4878	−26.9720	−11.9342	−21.8963	−6.5154	−17.2658	−8.5893	−15.4612
5	−6.3502	−6.5185	−8.0473	−14.7666	−6.1080	−13.4278	−4.2697	−10.0887	−2.4383	−9.4424
6	−6.9549	−6.9685	−9.3902	−23.4066	−9.9421	−21.4734	−4.5981	−15.5899	−5.0755	−13.3815
7	−4.2867	−0.3153	−6.8485	−12.4373	−9.2251	−15.5489	−3.0189	−6.7323	−5.4520	−11.3380
8	−8.7096	−13.5232	−11.8980	−21.1693	−12.6577	−26.1373	−10.4494	−20.4188	−6.5215	−16.2742
9	−6.6370	−4.3897	−9.1430	−10.4015	−9.0805	−10.1881	−3.9091	−4.3879	−3.8920	−4.1956

NICS-XY-Scan of oxaza[8]helicene **5a**

Path of NICS-XY-Scan	NICS(iso)-XY-Scan	NICS(zz)-XY-Scan
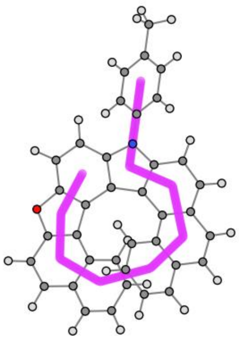	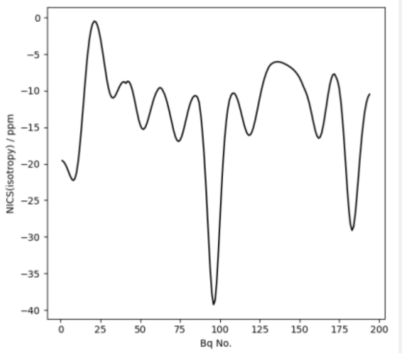	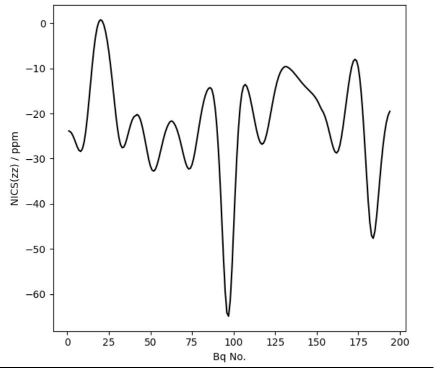

Integral NICS of oxaza[8]helicene **5a**

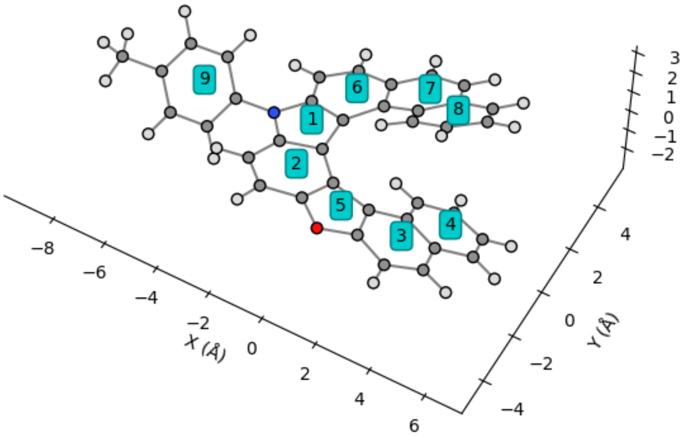

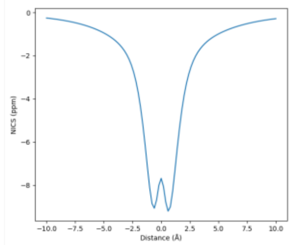
NICS(iso) plot of **ring 1**

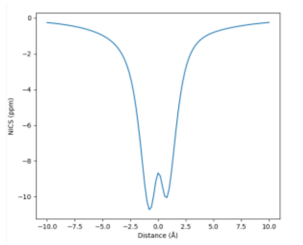
NICS(iso) plot of **ring 2**

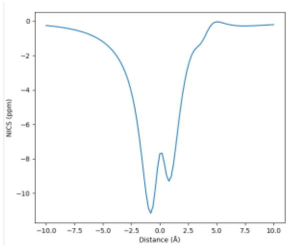
NICS(iso) plot of **ring 3**

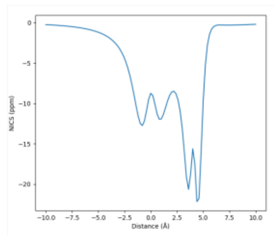
NICS(iso) plot of **ring 4**

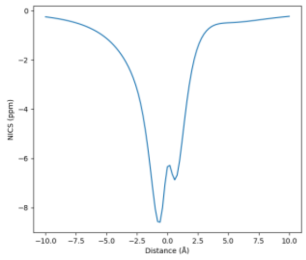
NICS(iso) plot of **ring 5**

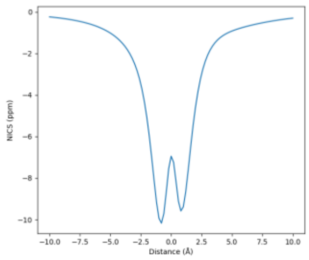
NICS(iso) plot of **ring 6**

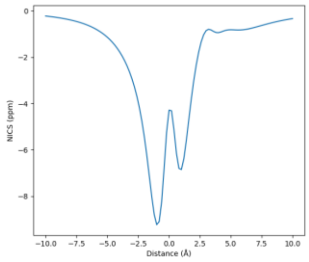
NICS(iso) plot of **ring 7**

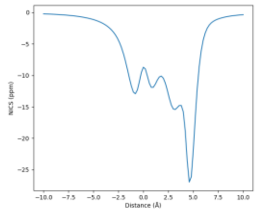
NICS(iso) plot of **ring 8**

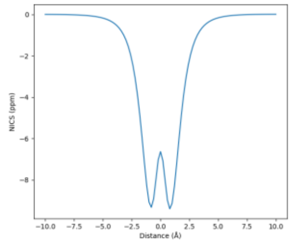
NICS(iso) plot of **ring 9**

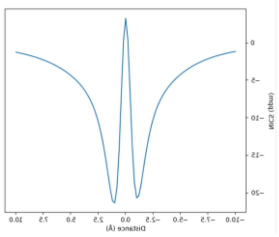
NICS(zz) plot of **ring 1**

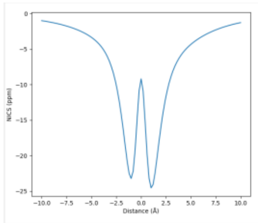
NICS(zz) plot of **ring 2**

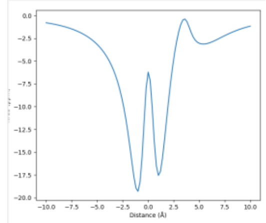
NICS(zz) plot of **ring 3**

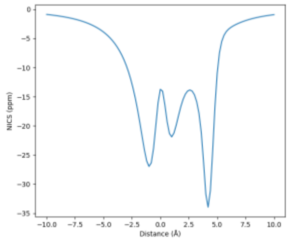
NICS(zz) plot of **ring 4**

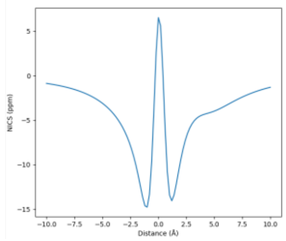
NICS(zz) plot of **ring 5**

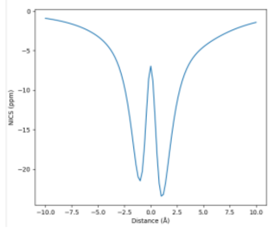
NICS(zz) plot of **ring 6**

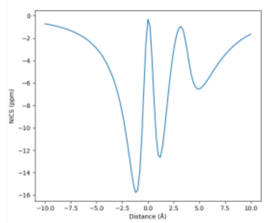
NICS(zz) plot of **ring 7**

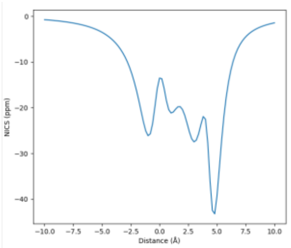

NICS(zz) plot of **ring 8**

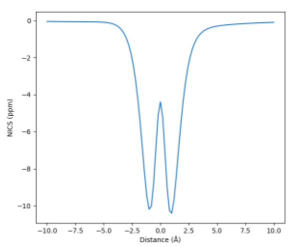

NICS(zz) plot of **ring 9**

2D NICS/ICSS of oxaza[8]helicene **5a**

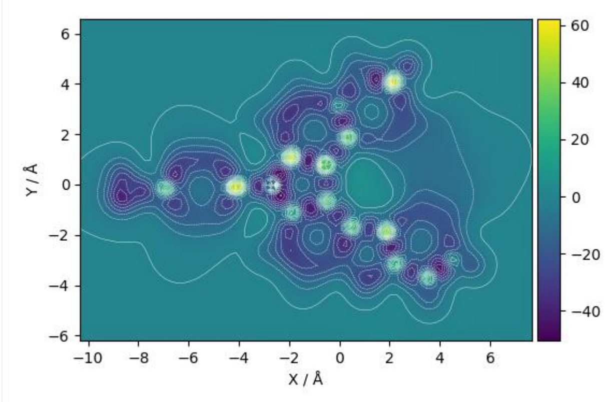

2D heatmap on the XY plane
Component of shielding tensors: isotropy

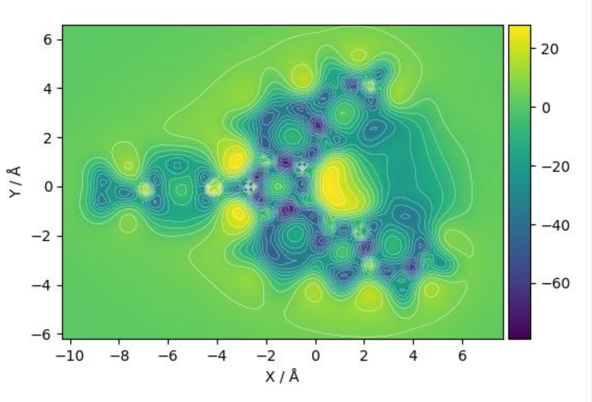

2D heatmap on the XY plane
Component of shielding tensors: zz

HOMA & HOMER of oxaza[8]helicene **5a**

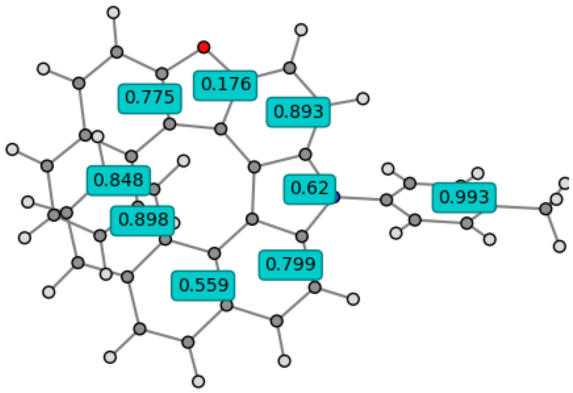

HOMA

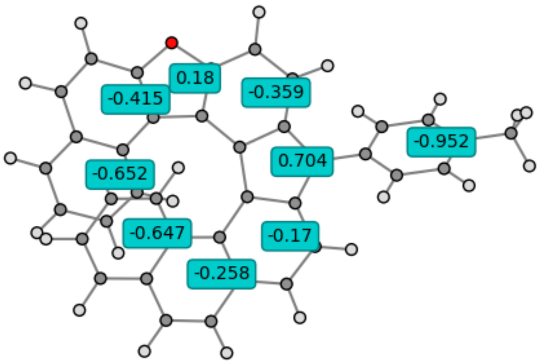

HOMER

Aromaticity of oxaza[8]helicene **5b**

calculated at MN15/6-311G(2d,p)/SMD=chloroform level of theory

NICS(r)iso and NICS(r)zz of oxaza[8]helicene **5b**

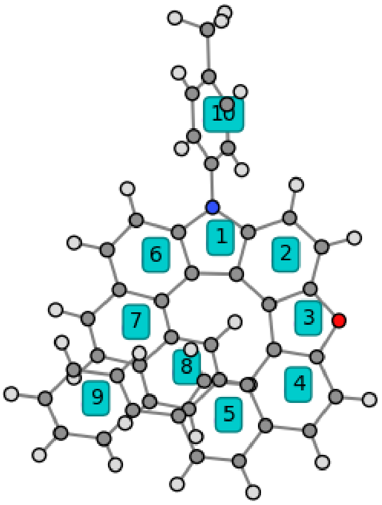

Ring	NICS(0)iso	NICS(0)zz	NICS(1)iso	NICS(1)zz	NICS(−1)iso	NICS(−1)zz	NICS(2)iso	NICS(2)zz	NICS(−2)iso	NICS(−2)zz
1	−7.6849	−2.6687	−8.1883	−20.4763	−8.1703	−20.9474	−3.7528	−12.9341	−3.6372	−13.2464
2	−8.4967	−9.2370	−9.3971	−23.9009	−10.1197	−25.5406	−4.3183	−14.9135	−4.8243	−16.2146
3	−6.3578	−8.3685	−6.0779	−14.7351	−8.0368	−18.0392	−2.3812	−9.6493	−4.1541	−13.2754
4	−7.1953	−6.6968	−8.7059	−21.2757	−10.3183	−23.8900	−5.5283	−12.6401	−3.9904	−16.6285
5	−8.1380	−13.1461	−11.4082	−28.8561	−11.5002	−29.5298	−5.9686	−22.2860	−8.3319	−19.0744
6	−7.0955	−7.1580	−10.0985	−22.9620	−9.4179	−22.1909	−5.1908	−14.8444	−4.4009	−12.9079
7	−5.0023	−3.5368	−9.5587	−20.3947	−8.2448	−17.8808	−5.6700	−15.6083	−6.8661	−14.7353
8	−8.7017	−17.4693	−12.5827	−31.7387	−11.8066	−29.5572	−6.5472	−20.2190	−10.6712	−29.7028
9	−7.3122	−11.6059	−10.9898	−25.3527	−10.3033	−23.4533	−8.6432	−20.0927	−5.0459	−14.1120
10	−6.6325	−1.5874	−9.0984	−1.8854	−9.1052	−2.0720	−3.8938	−1.4671	−3.8865	−1.1021

NICS-XY-Scan of oxaza[8]helicene **5b**

Path of NICS-XY-Scan	NICS(iso)-XY-Scan	NICS(zz)-XY-Scan
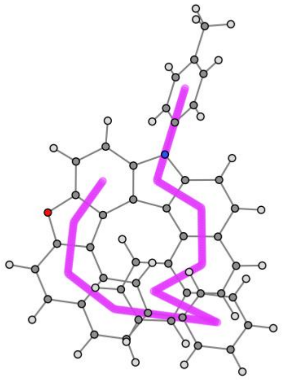	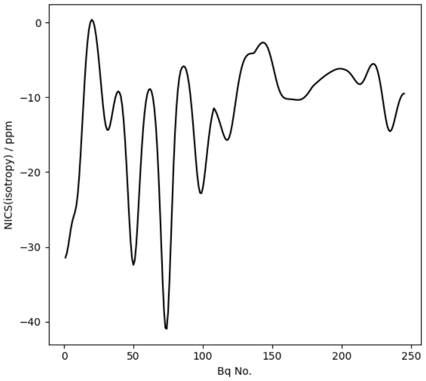	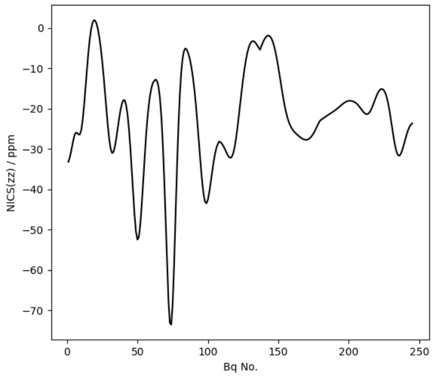

Integral NICS of oxaza[8]helicene **5b**

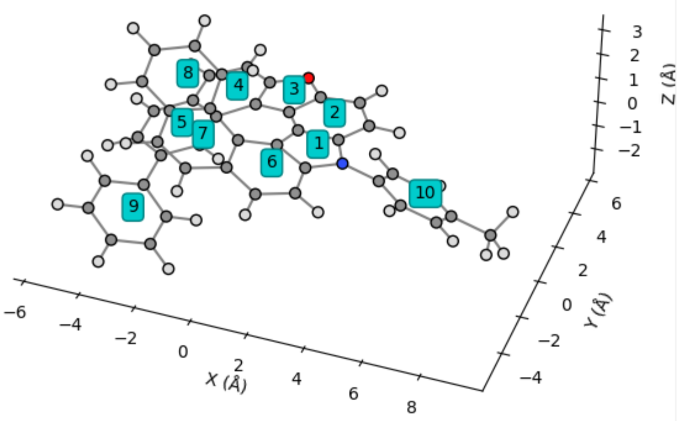

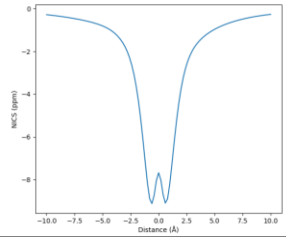

NICS(iso) plot of **ring 1**

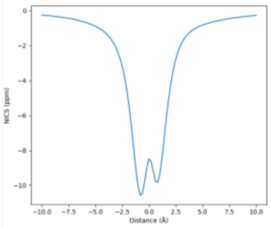

NICS(iso) plot of **ring 2**

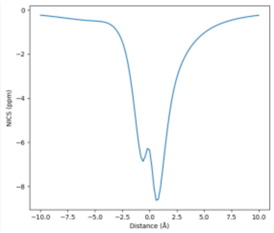

NICS(iso) plot of **ring 3**

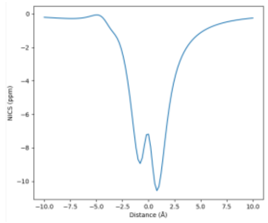

NICS(iso) plot of **ring 4**

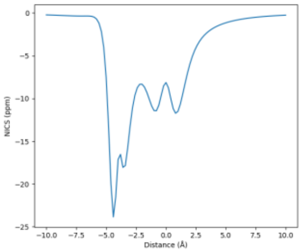

NICS(iso) plot of **ring 5**

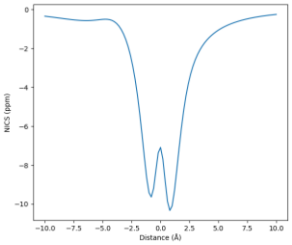

NICS(iso) plot of **ring 6**

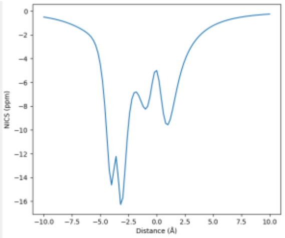

NICS(iso) plot of **ring 7**

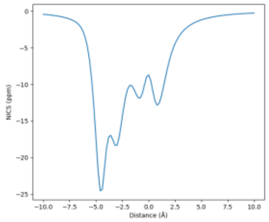

NICS(iso) plot of **ring 8**

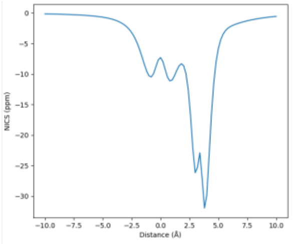

NICS(iso) plot of **ring 9**

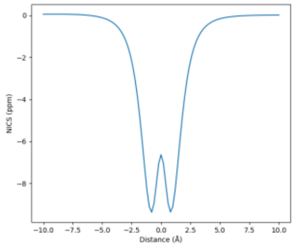

NICS(iso) plot of **ring 10**

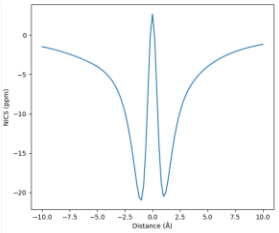

NICS(zz) plot of **ring 1**

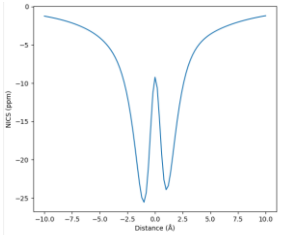

NICS(zz) plot of **ring 2**

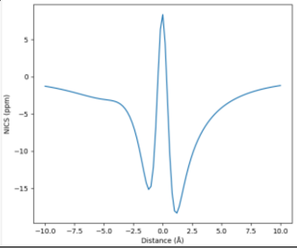

NICS(zz) plot of **ring 3**

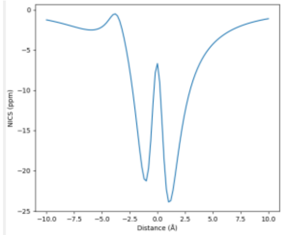

NICS(zz) plot of **ring 4**

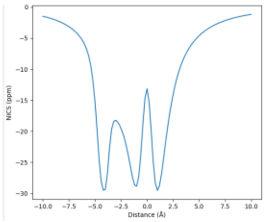

NICS(zz) plot of **ring 5**

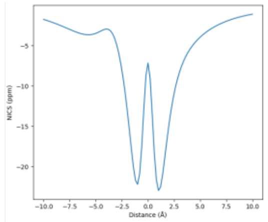

NICS(zz) plot of **ring 6**

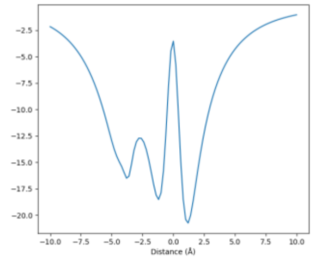

NICS(zz) plot of **ring 7**

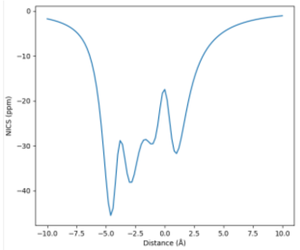

NICS(zz) plot of **ring 8**

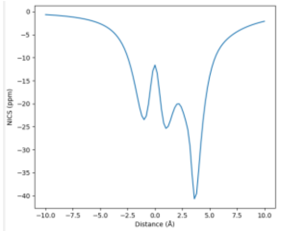

NICS(zz) plot of **ring 9**

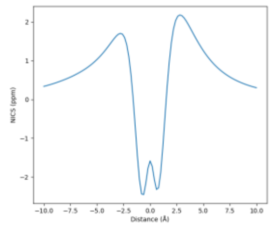

NICS(zz) plot of **ring 10**

2D NICS/ICSS of oxaza[8]helicene **5b**

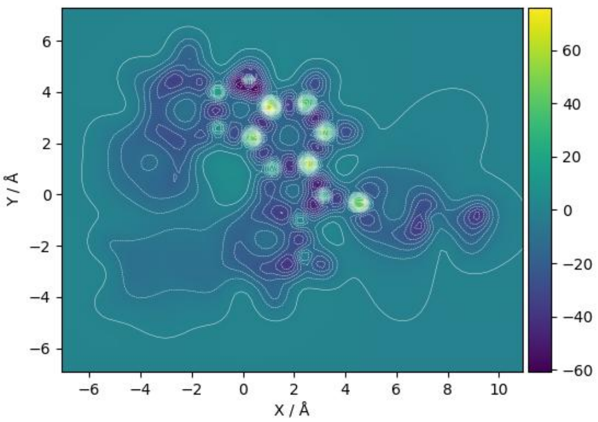

2D heatmap on the XY plane
Component of shielding tensors: isotropy

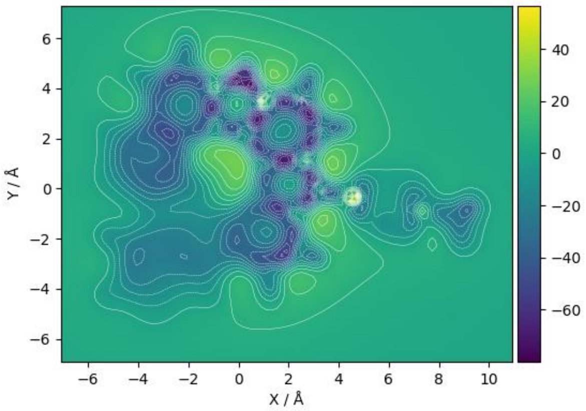

2D heatmap on the XY plane
Component of shielding tensors: zz

HOMA & HOMER of oxaza[8]helicene **5b**

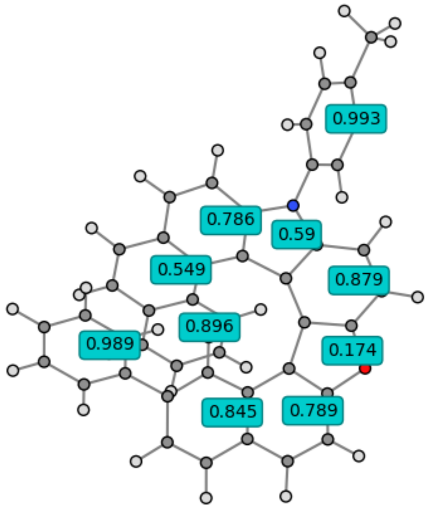

HOMA

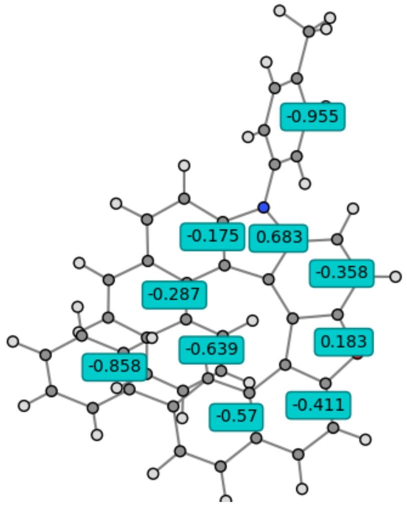

HOMER

<u>Aromaticity of oxaza[7]helicene **6a**</u>

calculated at MN15/6-311G(2d,p)/SMD=chloroform level of theory

NICS(r)iso and NICS(r)zz of oxaza[7]helicene **6a**

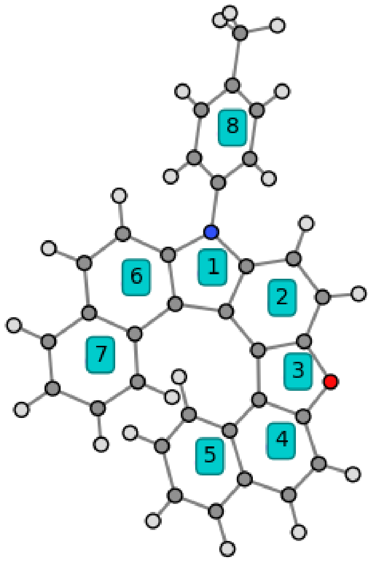

Ring	NICS(0)iso	NICS(0)zz	NICS(1)iso	NICS(1)zz	NICS(−1)iso	NICS(−1)zz	NICS(2)iso	NICS(2)zz	NICS(−2)iso	NICS(−2)zz
1	−8.3087	2.0407	−9.4589	−22.7069	−7.7606	−21.0183	−4.5867	−14.3857	−3.3544	−13.7096
2	−8.9425	−10.1419	−10.2814	−25.8592	−10.2716	−23.8268	−5.0295	−17.0420	−4.8411	−14.5659
3	−6.1553	6.6647	−6.1255	−14.5502	−7.9692	−14.6701	−2.7800	−11.5234	−4.1831	−9.9745
4	−7.4746	−6.2563	−8.6524	−18.0353	−10.8315	−19.1517	−4.1485	−11.7755	−5.6878	−12.1590
5	−7.7471	−10.7680	−9.9080	−16.0091	−11.8944	−24.6869	−4.3460	−6.1104	−6.1336	−15.7961
6	−6.4027	−4.1169	−10.1162	−21.0927	−8.1715	−19.8983	−5.3856	−14.2827	−4.1137	−14.2628
7	−7.6833	−11.2043	−11.8176	−27.8283	−9.7313	−20.3508	−6.0569	−17.5196	−4.1945	−9.5103
8	−6.7369	−4.1074	−9.1696	−9.0288	−9.2195	−8.9832	−3.9623	−3.7307	−3.9704	−3.2093

NICS-XY-Scan of oxaza[7]helicene **6a**

Path of NICS-XY-Scan	NICS(iso)-XY-Scan	NICS(zz)-XY-Scan
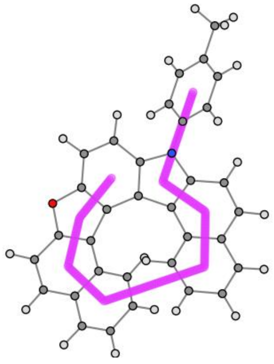	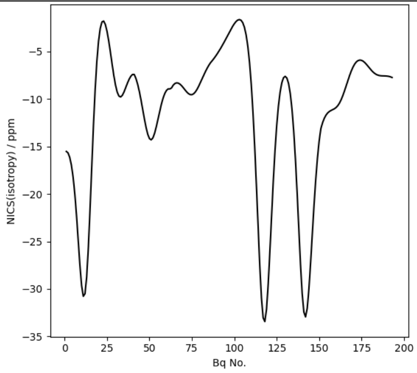	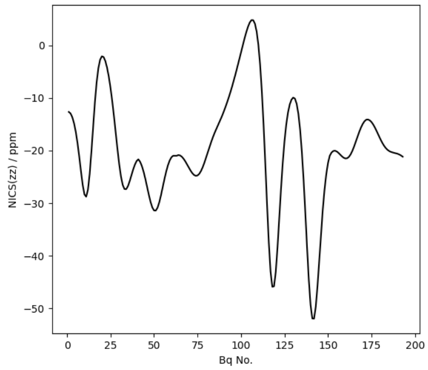

Integral NICS of oxaza[7]helicene **6a**

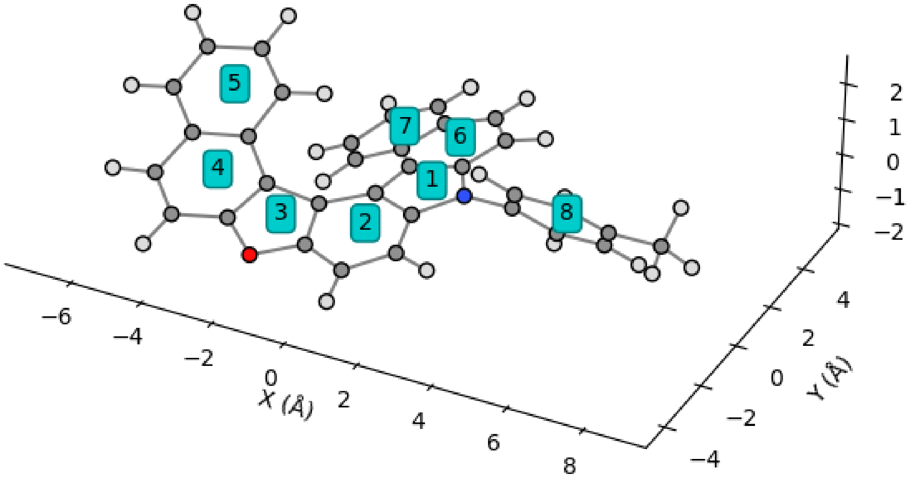

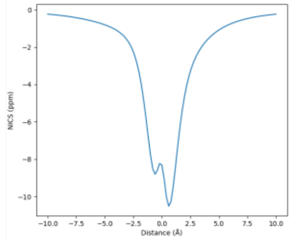	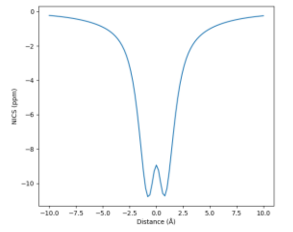	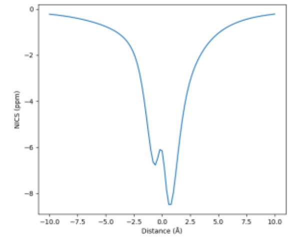	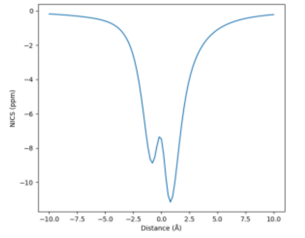
NICS(iso) plot of **ring 1**	NICS(iso) plot of **ring 2**	NICS(iso) plot of **ring 3**	NICS(iso) plot of **ring 4**
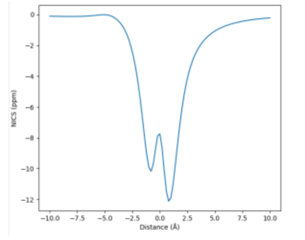	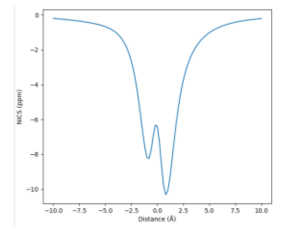	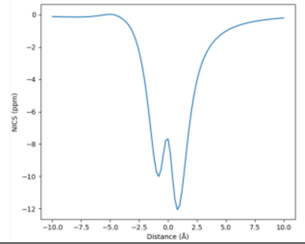	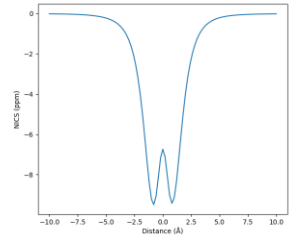
NICS(iso) plot of **ring 5**	NICS(iso) plot of **ring 6**	NICS(iso) plot of **ring 7**	NICS(iso) plot of **ring 8**
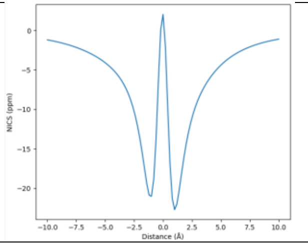	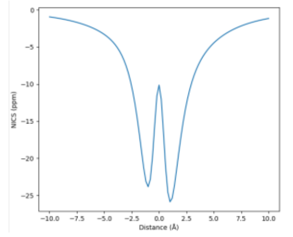	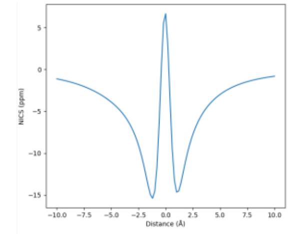	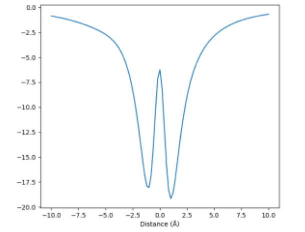
NICS(zz) plot of **ring 1**	NICS(zz) plot of **ring 2**	NICS(zz) plot of **ring 3**	NICS(zz) plot of **ring 4**
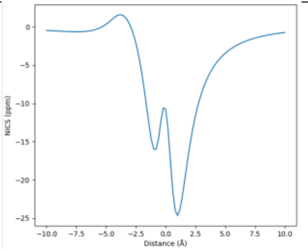	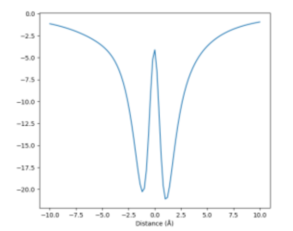	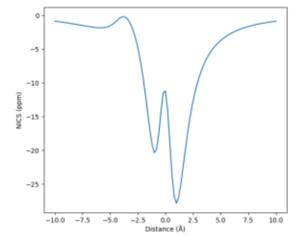	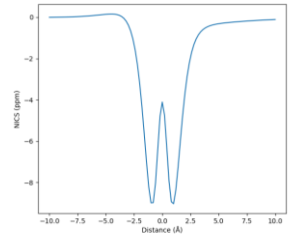
NICS(zz) plot of **ring 5**	NICS(zz) plot of **ring 6**	NICS(zz) plot of **ring 7**	NICS(zz) plot of **ring 8**

2D NICS/ICSS of oxaza[7]helicene **6a**

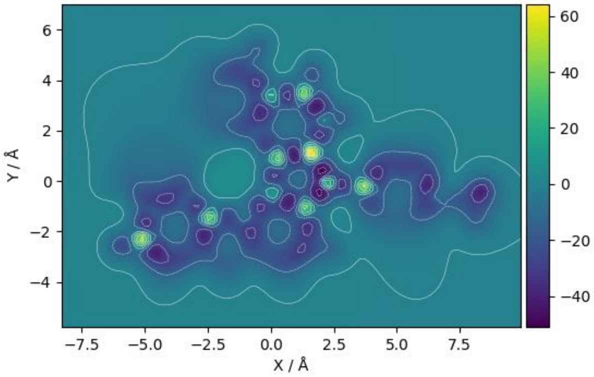

2D heatmap on the XY plane
Component of shielding tensors: isotropy

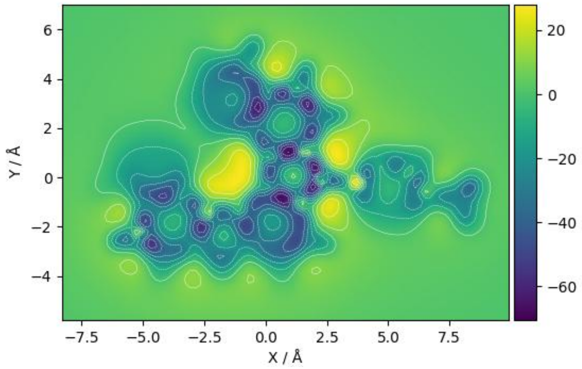

2D heatmap on the XY plane
Component of shielding tensors: zz

HOMA & HOMER of oxaza[7]helicene **6a**

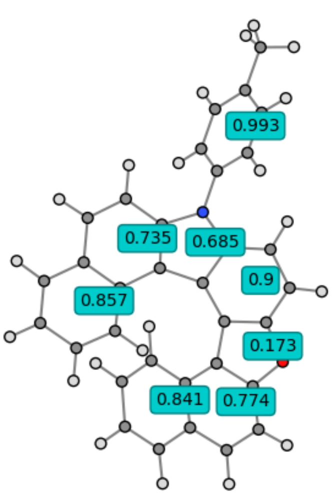

HOMA

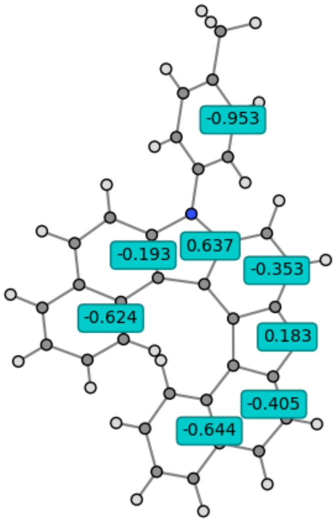

HOMER

Aromaticity of oxaza[7]helicene **6b**

calculated at MN15/6-311G(2d,p)/SMD=chloroform level of theory

NICS(r)iso and NICS(r)zz of oxaza[7]helicene **6b**

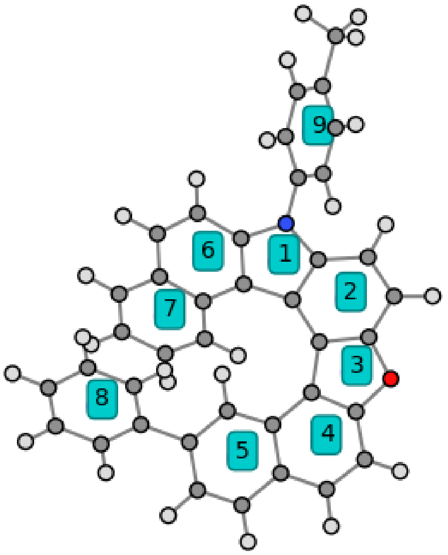

Ring	NICS(0)iso	NICS(0)zz	NICS(1)iso	NICS(1)zz	NICS(−1)iso	NICS(−1)zz	NICS(2)iso	NICS(2)zz	NICS(−2)iso	NICS(−2)zz
1	−8.3150	1.3510	−9.2025	−21.9716	−7.9986	−20.5720	−4.3940	−13.6072	−3.3946	−12.8897
2	−8.7488	−9.7255	−9.9118	−24.8346	−10.2832	−25.8586	−4.8303	−16.1068	−4.8095	−16.2758
3	−6.2651	8.9204	−6.1919	−15.6185	−7.9788	−17.5687	−2.7461	−11.7064	−4.0900	−12.8079
4	−7.0664	6.1361	−8.6268	−21.2623	−10.2274	−23.2406	−4.1546	−14.1826	−5.3532	−15.7598
5	−7.1652	−9.1458	−9.6218	−22.2722	−10.7812	−26.9891	−4.1581	−11.4255	−5.5543	−17.6373
6	−6.3721	−3.9477	−9.9594	−20.2640	−8.2919	−17.5414	−5.3412	−14.0229	−3.9510	−10.5795
7	−8.1833	−13.1759	−11.9969	−28.1300	−10.9515	−22.5163	−6.1801	−18.0539	−8.6863	−17.3792
8	−7.2525	−10.6049	−11.3873	−24.4980	−10.2039	−22.7086	−8.5246	−18.3957	−4.9885	−13.6138
9	−6.7255	−2.0520	−9.2035	−2.7227	−9.1665	−3.0339	−3.9671	0.9623	−3.9426	0.2914

NICS-XY-Scan of oxaza[7]helicene **6b**

Path of NICS-XY-Scan	NICS(iso)-XY-Scan	NICS(zz)-XY-Scan
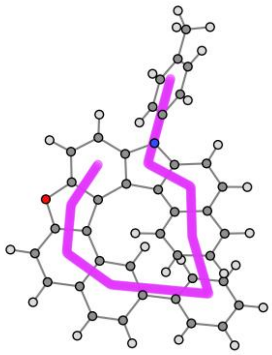	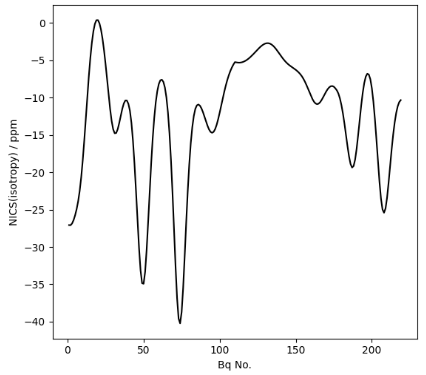	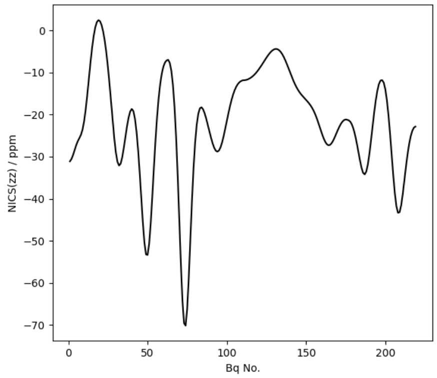

Integral NICS of oxaza[7]helicene **6b**

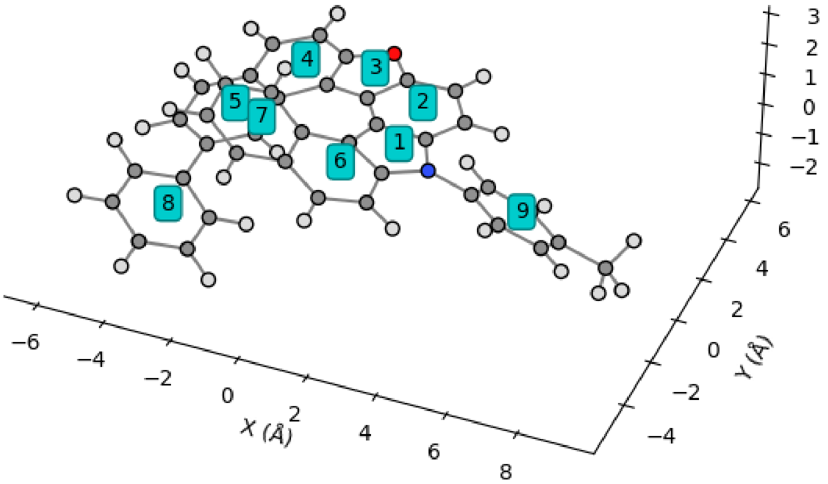

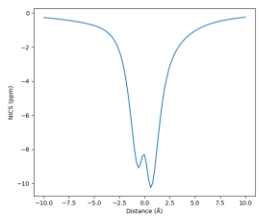
NICS(iso) plot of **ring 1**

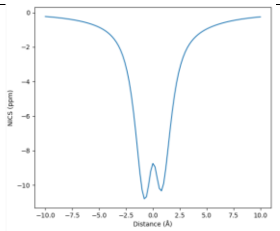
NICS(iso) plot of **ring 2**

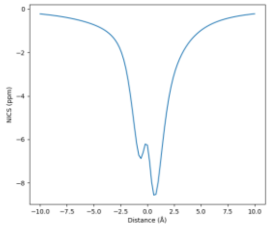
NICS(iso) plot of **ring 3**

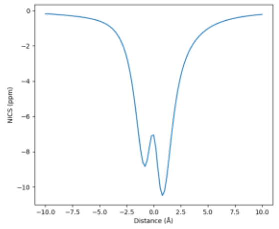
NICS(iso) plot of **ring 4**

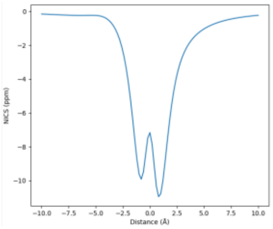
NICS(iso) plot of **ring 5**

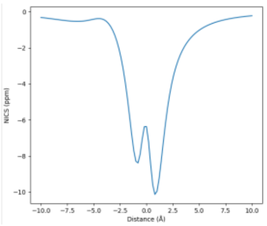
NICS(iso) plot of **ring 6**

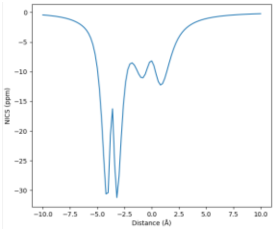
NICS(iso) plot of **ring 7**

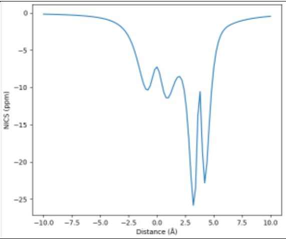
NICS(iso) plot of **ring 8**

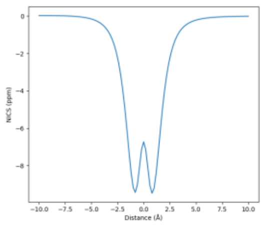
NICS(iso) plot of **ring 9**

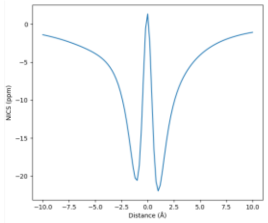
NICS(zz) plot of **ring 1**

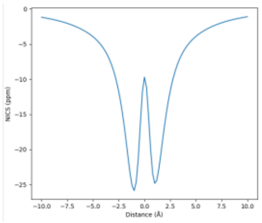
NICS(zz) plot of **ring 2**

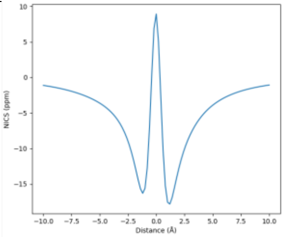
NICS(zz) plot of **ring 3**

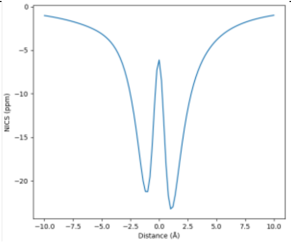

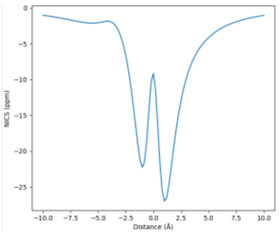

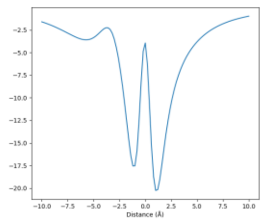

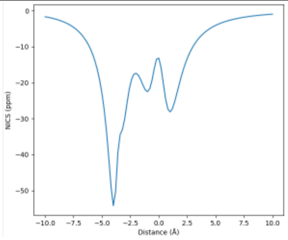

NICS(zz) plot of **ring 4**	NICS(zz) plot of **ring 5**	NICS(zz) plot of **ring 6**	NICS(zz) plot of **ring 7**
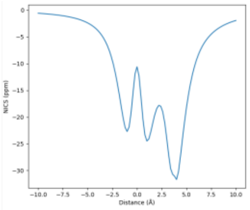	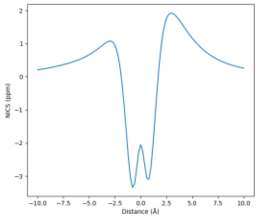		
NICS(zz) plot of **ring 8**	NICS(zz) plot of **ring 9**		

2D NICS/ICSS of oxaza[7]helicene **6b**

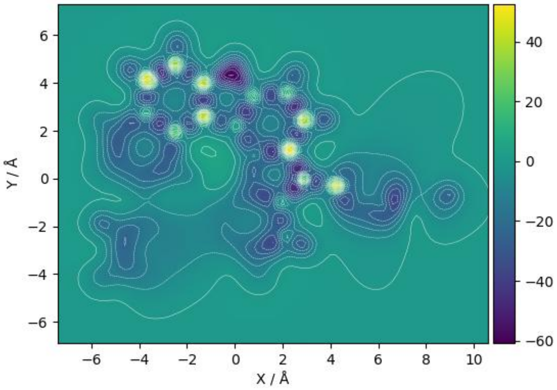

2D heatmap on the XY plane
Component of shielding tensors: isotropy

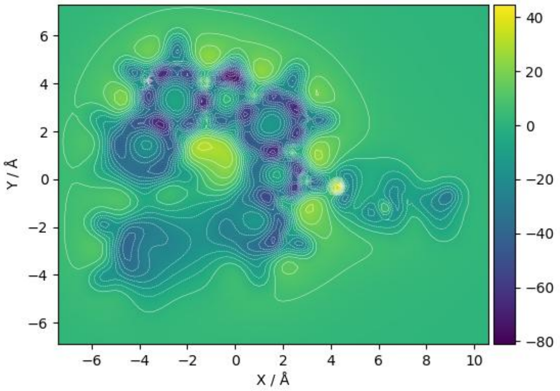

2D heatmap on the XY plane
Component of shielding tensors: zz

HOMA & HOMER of oxaza[7]helicene **6b**

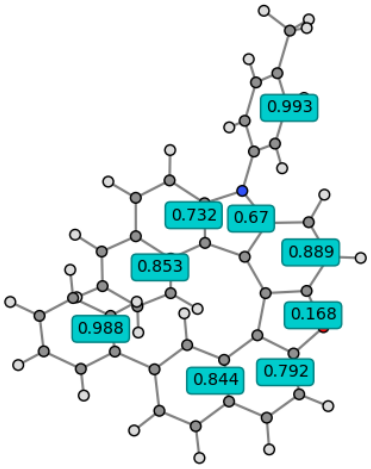

HOMA

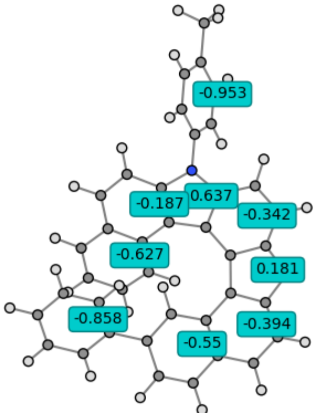

HOMER

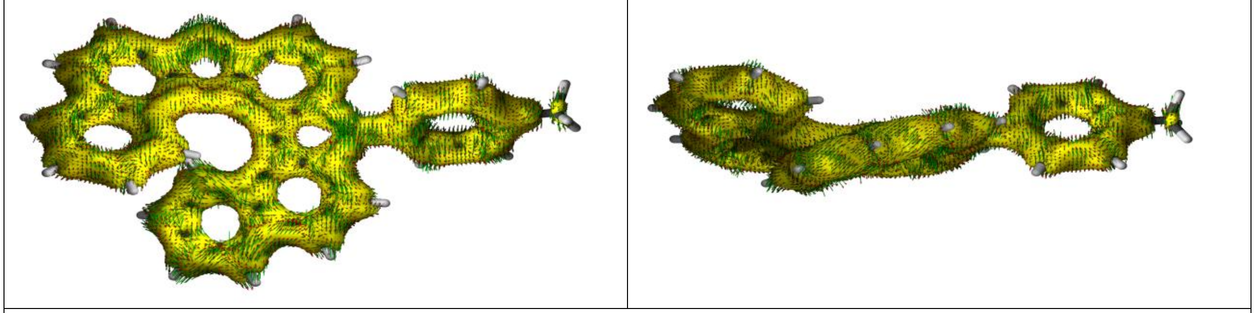

ACID plots of **6a** calculated at the B3LYP/6-311G(d,p) level of theory (isosurface value: 0.05).

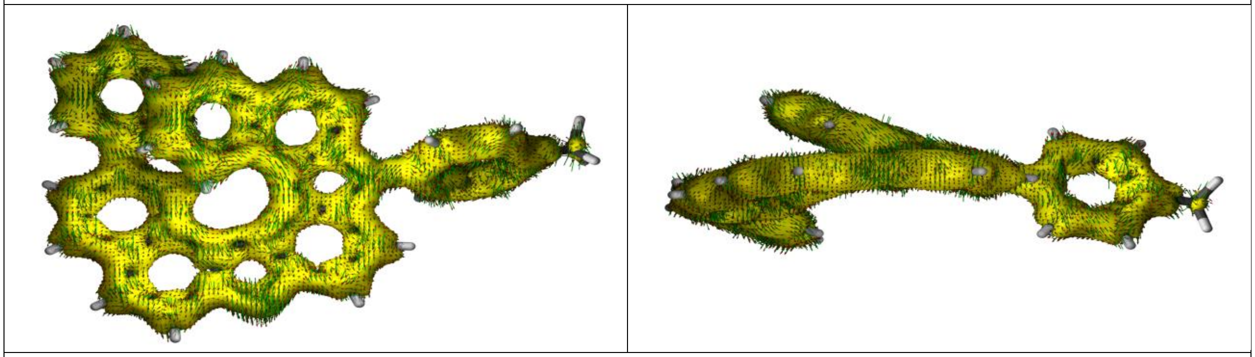

ACID plots of **6b** calculated at the B3LYP/6-311G(d,p) level of theory (isosurface value: 0.05).

3.3. Time-dependent density-functional theory (TD-DFT) calculations

Table S9. Summary of the TD-DFT calculation results of **5a** (S_0 state geometry) at MN15/6-311G(2d,p)/SMD=chloroform level of theory.

Excited states	Energy (eV)	Wavelength (nm)	Oscillator strength	Major contributions (%)		
S_1	3.2184	385.24	0.2849	HOMO	LUMO	93
S_2	3.5497	349.28	0.1384	HOMO−1	LUMO	89
				HOMO	LUMO+1	3
S_3	3.9157	316.64	0.0772	HOMO−2	LUMO	37
				HOMO	LUMO+2	26
				HOMO−1	LUMO+1	19
				HOMO	LUMO+1	5

S_1~S_3

Table S10. Summary of the TD-DFT calculation results of **5b** (S_0 state geometry) at MN15/6-311G(2d,p)/SMD=chloroform level of theory.

Excited states	Energy (eV)	Wavelength (nm)	Oscillator strength	Major contributions (%)		
S_1	3.1482	393.83	0.2208	HOMO	LUMO	93
S_2	3.5229	351.94	0.1093	HOMO−1	LUMO	86
				HOMO	LUMO+1	5
S_3	3.8247	324.17	0.2923	HOMO−2	LUMO	32
				HOMO−1	LUMO+1	6
				HOMO−1	LUMO+2	7
				HOMO	LUMO+1	28
				HOMO	LUMO+2	12

S_1~S_3

Table S11. Summary of the TD-DFT calculation results of **6a** (S_0 state geometry) at MN15/6-311G(2d,p)/SMD=chloroform level of theory.

Excited states	Energy (eV)	Wavelength (nm)	Oscillator strength	Major contributions (%)		
S_1	3.3483	370.29	0.4725	HOMO	LUMO	95
S_2	3.7374	331.74	0.246	HOMO−1	LUMO	86
				HOMO	LUMO+4	4
S_3	4.0938	302.86	0.1291	HOMO−2	LUMO	29
				HOMO−1	LUMO+2	2
				HOMO	LUMO+1	57

S_1~S_3

Table S12. Summary of the TD-DFT calculation results of **6b** (S_0 state geometry) at MN15/6-311G(2d,p)/SMD=chloroform level of theory.

Excited states	Energy (eV)	Wavelength (nm)	Oscillator strength	Major contributions (%)		
S_1	3.2279	384.1	0.3322	HOMO	LUMO	95
S_2	3.6851	336.45	0.259	HOMO−1	LUMO	84
				HOMO−1	LUMO+1	3
				HOMO	LUMO+2	3
S_3	3.9633	312.83	0.2679	HOMO−3	LUMO	2
				HOMO−2	LUMO	19
				HOMO	LUMO+1	65

S_1~S_3

Table S13. Summary of the TD-DFT calculation results of **5a** (S_1 state geometry) at MN15/6-311G(2d,p)/SMD=chloroform level of theory.

Excited states	Energy (eV)	Wavelength (nm)	Oscillator strength	Major contributions (%)		
S_1	2.7622	448.86	0.3146	HOMO	LUMO	96
S_2	3.2909	376.75	0.0947	HOMO−1	LUMO	88
				HOMO	LUMO+1	4
S_3	3.7281	332.57	0.1648	HOMO−2	LUMO	51
				HOMO−1	LUMO+1	13
				HOMO	LUMO+1	3
				HOMO	LUMO+2	23
S_4	3.7751	328.42	0.5069	HOMO−1	LUMO	5
				HOMO	LUMO+1	83
S_5	3.9293	315.54	0.0037	HOMO−2	LUMO	33
				HOMO	LUMO+2	58
S_6	4.04	306.89	0.0603	HOMO−4	LUMO	7
				HOMO−3	LUMO	34
				HOMO−1	LUMO+1	2
				HOMO−1	LUMO+2	4
				HOMO	LUMO+2	3
				HOMO	LUMO+3	25
				HOMO	LUMO+5	3
				HOMO	LUMO+6	5
S_7	4.1192	300.99	0.0726	HOMO−5	LUMO	5
				HOMO−4	LUMO	31
				HOMO−2	LUMO	6

				HOMO−1	LUMO+1	10
				HOMO−1	LUMO+2	12
				HOMO	LUMO+3	18
				HOMO	LUMO+6	5
S_8	4.2737	290.11	0.1386	HOMO−4	LUMO	17
				HOMO−3	LUMO	15
				HOMO−1	LUMO+1	46
				HOMO	LUMO+2	8
S_9	4.3452	285.34	0.1749	HOMO−4	LUMO	10
				HOMO−3	LUMO	24
				HOMO−1	LUMO+1	11
				HOMO−1	LUMO+2	4
				HOMO	LUMO+3	43
S_{10}	4.4801	276.75	0.1022	HOMO−4	LUMO	4
				HOMO−3	LUMO	6
				HOMO−1	LUMO+2	50
				HOMO	LUMO+4	24

S_1~S_{10}

Table S14. Summary of the TD-DFT calculation results of **5b** (S_1 state geometry) at MN15/6-311G(2d,p)/SMD=chloroform level of theory.

Excited states	Energy (eV)	Wavelength (nm)	Oscillator strength	Major contributions (%)		
S_1	2.6301	471.4	0.2234	HOMO	LUMO	96
S_2	3.2443	382.16	0.063	HOMO−1	LUMO	86
				HOMO	LUMO+1	6
S_3	3.5866	345.69	0.4981	HOMO−2	LUMO	17
				HOMO−1	LUMO	5
				HOMO−1	LUMO+2	4
				HOMO	LUMO+1	60
				HOMO	LUMO+2	3
S_4	3.6648	338.31	0.0561	HOMO−2	LUMO	44
				HOMO−1	LUMO	2
				HOMO−1	LUMO+2	3
				HOMO	LUMO+1	25
				HOMO	LUMO+2	20
S_5	3.7666	329.17	0.1763	HOMO−2	LUMO	25

				HOMO−1	LUMO+1	2
				HOMO	LUMO+2	60
S_6	3.8409	322.8	0.1682	HOMO−3	LUMO	53
				HOMO−1	LUMO+1	6
				HOMO	LUMO+2	5
				HOMO	LUMO+3	9
				HOMO	LUMO+4	9
				HOMO	LUMO+5	3
S_7	4.0319	307.51	0.0522	HOMO−6	LUMO	3
				HOMO−4	LUMO	14
				HOMO−2	LUMO	5
				HOMO−1	LUMO+1	2
				HOMO−1	LUMO+2	19
				HOMO	LUMO+3	39
				HOMO	LUMO+4	4
S_8	4.1714	297.22	0.0595	HOMO−6	LUMO	3
				HOMO−4	LUMO	19
				HOMO−3	LUMO	29
				HOMO−1	LUMO+1	5
				HOMO−1	LUMO+2	5
				HOMO	LUMO+2	3
				HOMO	LUMO+3	11
				HOMO	LUMO+4	16
S_9	4.2505	291.7	0.1008	HOMO−4	LUMO	6
				HOMO−1	LUMO+1	64
				HOMO−1	LUMO+2	5
				HOMO	LUMO+2	2
				HOMO	LUMO+3	9
				HOMO	LUMO+4	4
S_{10}	4.2992	288.39	0.2822	HOMO−4	LUMO	11
				HOMO−3	LUMO	6
				HOMO−1	LUMO+1	7
				HOMO−1	LUMO+2	44
				HOMO	LUMO+3	20

S_1~S_{10}

Table S15. Summary of the TD-DFT calculation results of **6a** (S_1 state geometry) at MN15/6-311G(2d,p)/SMD=chloroform level of theory.

Excited states	Energy (eV)	Wavelength (nm)	Oscillator strength	Major contributions (%)		
S_1	2.8262	438.7	0.4852	HOMO	LUMO	97
S_2	3.5281	351.42	0.2441	HOMO−1	LUMO	86
				HOMO	LUMO+5	5
S_3	3.8194	324.62	0.1513	HOMO−2	LUMO	16
				HOMO	LUMO+1	77
S_4	4.028	307.8	0.0569	HOMO−2	LUMO	67
				HOMO−1	LUMO+1	3
				HOMO	LUMO+1	19
S_5	4.0848	303.53	0.0021	HOMO−3	LUMO	17
				HOMO−2	LUMO	3
				HOMO−2	LUMO+5	3
				HOMO−1	LUMO+1	3
				HOMO	LUMO+2	55
				HOMO	LUMO+4	3
				HOMO	LUMO+5	3
				HOMO	LUMO+6	4
S_6	4.1924	295.74	0.0484	HOMO−4	LUMO	8
				HOMO−3	LUMO	21
				HOMO−3	LUMO+1	3
				HOMO−1	LUMO+1	7
				HOMO	LUMO+2	32
				HOMO	LUMO+3	5
				HOMO	LUMO+4	2
				HOMO	LUMO+5	7
				HOMO	LUMO+6	7
S_7	4.3646	284.07	0.24	HOMO−5	LUMO	3
				HOMO−3	LUMO	3
				HOMO−2	LUMO	5
				HOMO−2	LUMO+3	3
				HOMO−1	LUMO+2	2
				HOMO	LUMO+3	40
				HOMO	LUMO+4	5
				HOMO	LUMO+5	21
				HOMO	LUMO+6	2
S_8	4.5351	273.39	0.0473	HOMO−4	LUMO	3

				HOMO−1	LUMO	4
				HOMO−1	LUMO+1	25
				HOMO−1	LUMO+3	3
				HOMO	LUMO+3	31
				HOMO	LUMO+5	20
				HOMO	LUMO+6	3
S_9	4.6529	266.47	0.1252	HOMO−4	LUMO	47
				HOMO−3	LUMO	30
				HOMO	LUMO+2	2
				HOMO	LUMO+5	5
				HOMO	LUMO+6	3
S_{10}	4.702	263.68	0.2386	HOMO−4	LUMO	3
				HOMO−3	LUMO	4
				HOMO−1	LUMO+1	21
				HOMO−1	LUMO+3	2
				HOMO	LUMO+3	4
				HOMO	LUMO+4	27
				HOMO	LUMO+5	2
				HOMO	LUMO+6	21

S_1~S_{10}

Table S16. Summary of the TD-DFT calculation results of **6b** (S_1 state geometry) at MN15/6-311G(2d,p)/SMD=chloroform level of theory.

Excited states	Energy (eV)	Wavelength (nm)	Oscillator strength	Major contributions (%)		
S_1	2.6652	465.19	0.307	HOMO	LUMO	97
S_2	3.449	359.48	0.2588	HOMO−1	LUMO	84
S_3	3.6602	338.74	0.3576	HOMO−2	LUMO	8
				HOMO−1	LUMO	3
				HOMO	LUMO+1	81
S_4	3.7972	326.51	0.0586	HOMO−3	LUMO	2
				HOMO−2	LUMO	68
				HOMO−1	LUMO+1	5
				HOMO	LUMO+1	9
				HOMO	LUMO+3	6
S_5	3.9401	314.68	0.0474	HOMO−3	LUMO	38
				HOMO−2	LUMO	8
				HOMO	LUMO+1	3

				HOMO	LUMO+2	3
				HOMO	LUMO+3	32
				HOMO	LUMO+4	3
S_6	4.0269	307.89	0.0761	HOMO−5	LUMO	4
				HOMO−3	LUMO	7
				HOMO	LUMO+2	71
				HOMO	LUMO+3	3
S_7	4.2514	291.63	0.4405	HOMO−5	LUMO	9
				HOMO−3	LUMO	24
				HOMO−2	LUMO	7
				HOMO−1	LUMO+1	5
				HOMO	LUMO+3	20
				HOMO	LUMO+4	11
				HOMO	LUMO+6	7
S_8	4.2914	288.91	0.2334	HOMO−5	LUMO	2
				HOMO−3	LUMO	14
				HOMO−1	LUMO	3
				HOMO−1	LUMO+1	16
				HOMO	LUMO+2	10
				HOMO	LUMO+3	24
				HOMO	LUMO+4	14
				HOMO	LUMO+6	4
S_9	4.4174	280.67	0.1182	HOMO−4	LUMO	9
				HOMO−3	LUMO	4
				HOMO−1	LUMO+1	37
				HOMO	LUMO+4	35
S_{10}	4.5464	272.71	0.0367	HOMO−5	LUMO	44
				HOMO−4	LUMO	2
				HOMO	LUMO+2	6
				HOMO	LUMO+5	2
				HOMO	LUMO+6	28
				HOMO	LUMO+7	3

S_1~S_{10}

Table S17. Screening TD-DFT calculations of *oxaza[n]helicenes* using different basis sets

	Level of theory	$S_0 \rightarrow S_1$ transitions				
		$\lvert\mu\rvert$ (10^{-20} esu cm)[a]	$\lvert m\rvert$ (10^{-20} erg G^{-1})[b]	$\cos(\theta_{\mu,m})$[c]	(R) (10^{-40} (erg esu cm G^{-1})[d]	g_{cal}[e] (10^{-3})
5b	MN15/6-311+g(2d,p) (smd, solvent=chloroform)	471.9	2.7	−0.174	−219.6	−0.0039
	MN15/6-31+g(2d,p) (smd, solvent=chloroform)	472.7	2.7	−0.173	−218.1	−0.0039
	MN15/6-31+g(d,p) (smd, solvent=chloroform)	474.9	2.6	−0.167	−207.1	−0.0037
	MN15/6-311g(d,p) (smd, solvent=chloroform)	477.1	2.6	−0.184	−229.6	−0.0040
	MN15/cc-pvdz (smd, solvent=chloroform)	481.0	2.6	−0.191	−239.0	−0.0041
	MN15/lanl2dz (smd, solvent=chloroform)	477.5	2.3	−0.173	−189.4	−0.0033
	MN15/lanl2mb (smd, solvent=chloroform)	512.3	1.8	−0.147	−136.8	−0.0021
	MN15/dgdzvp2 (smd, solvent=chloroform)	476.8	2.6	−0.177	−221.9	−0.0039
	MN15/6-311g(2d,p) (iefpcm, solvent=chloroform)	463.4	2.6	−0.186	−228.9	−0.0043
	MN15/lanl2mb gas phase	450.4	1.8	−0.193	−156.6	−0.0031
	MN15/lanl2dz gas phase	399.4	2.3	−0.214	−196.3	−0.0049
	MN15/lanl2mb (cpcm, solvent=chloroform)	509.9	1.8	−0.147	−136.1	−0.0021
	MN15/lanl2dz (cpcm, solvent=chloroform)	478.3	2.3	−0.176	−191.6	−0.0034
	MN15/lanl2mb (iefpcm, solvent=chloroform)	502.4	1.8	−0.151	−137.3	−0.0022
	MN15/lanl2dz (iefpcm, solvent=chloroform)	467.1	2.3	−0.176	−187.8	−0.0034
	MN15/lanl2mb (ipcm, solvent=chloroform)	450.4	1.8	−0.193	−156.7	−0.0031
	MN15/lanl2dz/qzv (smd, solvent=chloroform)	477.5	2.3	−0.173	−189.4	−0.0033
	MN15/lanl2dz/dga2 (smd, solvent=chloroform)	477.5	2.3	−0.173	−189.4	−0.0033
	MN15/lanl2dz/def2sv (smd, solvent=chloroform)	477.5	2.3	−0.173	−189.4	−0.0033
	MN15/lanl2mb/def2sv (smd, solvent=chloroform)	512.3	1.8	−0.147	−136.8	−0.0021
	MN15/lanl2mb/qzv (smd, solvent=chloroform)	512.3	1.8	−0.147	−136.8	−0.0021
	MN15/lanl2mb/dga2 (smd, solvent=chloroform)	512.3	1.8	−0.147	−136.8	−0.0021
6b	MN15/lanl2mb scrf=(ipcm, solvent=chloroform)	514.9	1.9	−0.143	−137.2	−0.0021
	MN15/lanl2mb scrf=(iefpcm, solvent=chloroform)	569.0	1.9	−0.114	−121.0	−0.0015
5a	MN15/lanl2mb scrf=(iefpcm, solvent=chloroform)	553.8	1.2	−0.103	−68.7	−0.0009
	MN15/lanl2mb scrf=(ipcm, solvent=chloroform)	494.2	1.2	−0.142	−84.0	−0.0014
6a	MN15/lanl2mb scrf=(iefpcm, solvent=chloroform)	665.4	1.1	−0.078	−54.8	−0.0005

[a] *Electric transition dipole moments (ETDM) for the $S_0 \rightarrow S_1$ transitions.* [b] *Magnetic transition dipole moments (MTDM) for the $S_0 \rightarrow S_1$ transitions.* [c] *The angle between ETDM and MTDM vectors.* [d] *Rotational strength.* [e] *Dimensionless values.*

Theoretically calculated radiative rate constants $k_{f,calcd}$

$$k_{f,calcd} = \frac{16\pi^3}{3h\varepsilon_0} \nu^3 (D + G) \quad Equation\ (S1)$$

Table S18. Calculation of the radiative rate constants $k_{f,calcd}$ of oxaza[*n*]helicenes

	$\lambda_{ex}{}^a$ /nm	v^b /cm^{-1}	v^3 (× 10^{13})	(D) (×10^{-40} (esu^2 cm^2)^c	(G) (×10^{-40} (erg^2 G^{-2})^d	(D + G) (×10^{-40})^e	$k_{f,calcd}$ /ns^{-1}	Φ_f
**5a**	381	26246.7	1.8	306733.0	1.439	306734.5	0.17370	25.1%
**5b**	368	27173.9	2.0	252356.9	3.280	252360.2	0.15859	22.6%
**6a**	385	25974.0	1.8	442754.5	1.128	442755.6	0.24299	40.1%
**6b**	374	26738.0	1.9	323757.6	3.485	323761.1	0.19383	38.9%

[a] For the $S_1 \rightarrow S_0$ transitions. [b] Deexcitation energy (in cm^{-1}) was converted as follows: v (cm^{-1}) = 10^7 / λ_{ex} (nm). [c] Electric dipole strength = $|\mu|^2$. [d] Magnetic dipole strength = $|m|^2$. [e] Calculated at MN15/lanl2mb (iefpcm, solvent=chloroform) level of theory.

ETDM and MTDM of oxaza[8]helicenes and oxaza[7]helicenes

Table S19. Calculation of ETDM and MTDM of oxaza[8]helicenes **5a** & **5b** and oxaza[7]helicenes **6a** & **6b**

	$S_1 \rightarrow S_0$ transition											
	$\|\mu_x\|$	$\|\mu_y\|$	$\|\mu_z\|$	$\|\mu'\|$	$\|m_x\|$	$\|m_y\|$	$\|m_z\|$	$\|m'\|$	$\theta_{\mu,m}$ (deg)^c	cos($\theta_{\mu,m}$)	(R) (×10^{-40} (erg esu cm G^{-1})^d	$g_{cal}{}^e$ (×10^3)
	(×10^{-20} esu cm)^a				(×10^{-20} erg G^{-1})^b							
**5a**	−437.7	−327.2	89.6	553.8	−0.2	0.2	−1.2	1.20	95.9	−0.103	−68.7	0.90
**5b**	−434.6	178.6	−177.7	502.4	−0.3	0.2	1.8	1.81	98.7	−0.151	−137.3	2.18
**6a**	−560.1	−354.6	57.1	665.4	−0.2	0.3	−1.0	1.06	94.5	−0.078	−54.8	0.50
**6b**	−497.5	236.8	−141.9	569.0	−0.3	−0.1	1.8	1.87	96.5	−0.114	−121.0	1.50

[a] Electric transition dipole moments (ETDM) for the $S_1 \rightarrow S_0$ transitions. [b] Magnetic transition dipole moments (MTDM) for the $S_1 \rightarrow S_0$ transitions. [c] The angle between ETDM and MTDM vectors. [d] Rotational strength. [e] Dimensionless values. Calculated at MN15/lanl2mb (iefpcm, solvent=chloroform) level of theory.

Simulated ECD spectrum of (*M*)-oxaza[7] & oxaza[8]helicenes

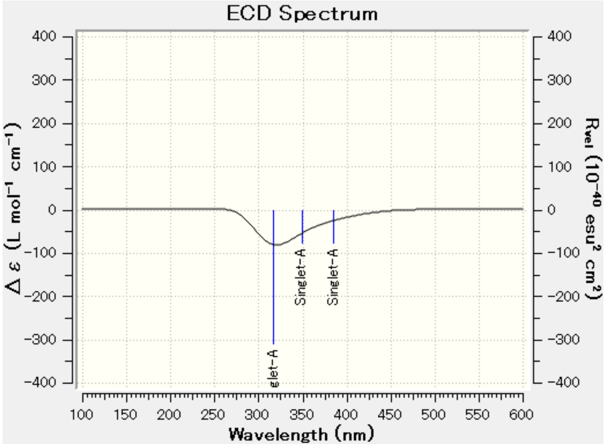

Simulated ECD spectrum of (*M*)−**5a** at MN15/6−311G(2d,p) (SMD, solvent=chloroform); (Wavelength (nm) = 316.64, R_{vel} (10^{-40} esu^2 cm^2) = −308.3914.

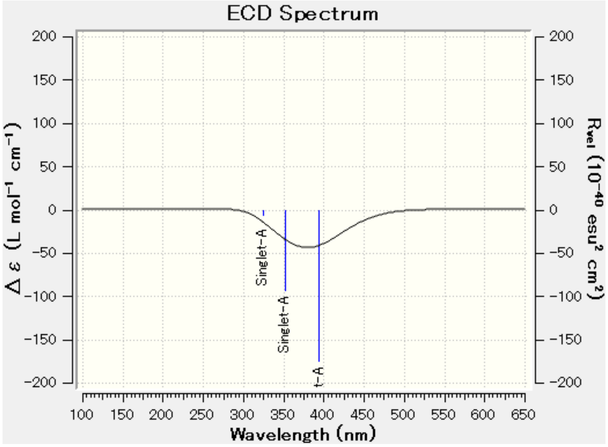

Simulated ECD spectrum of (*M*)−**5b** at MN15/6−311G(2d,p) (SMD, solvent=chloroform); (Wavelength (nm) = 393.83, R_{vel} (10^{-40} esu^2 cm^2) = −174.9947.

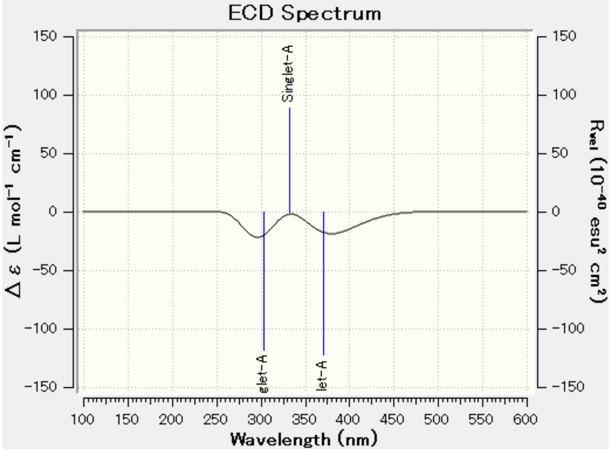

Simulated ECD spectrum of (*M*)−**6a** at MN15/6−311G(2d,p) (SMD, solvent=chloroform); (Wavelength (nm) = 302.86, R_{vel} (10^{-40} esu^2 cm^2) = −117.5699.

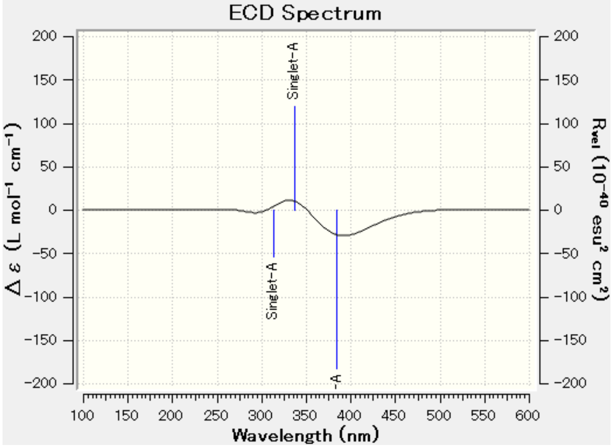

Simulated ECD spectrum of (*M*)−**6b** at MN15/6−311G(2d,p) (SMD, solvent=chloroform); (Wavelength (nm) = 384.10, R_{vel} (10^{-40} esu^2 cm^2) = −182.8564.

4. *Calculation of band gap energy from UV–Vis absorption (Tauc Plots)*

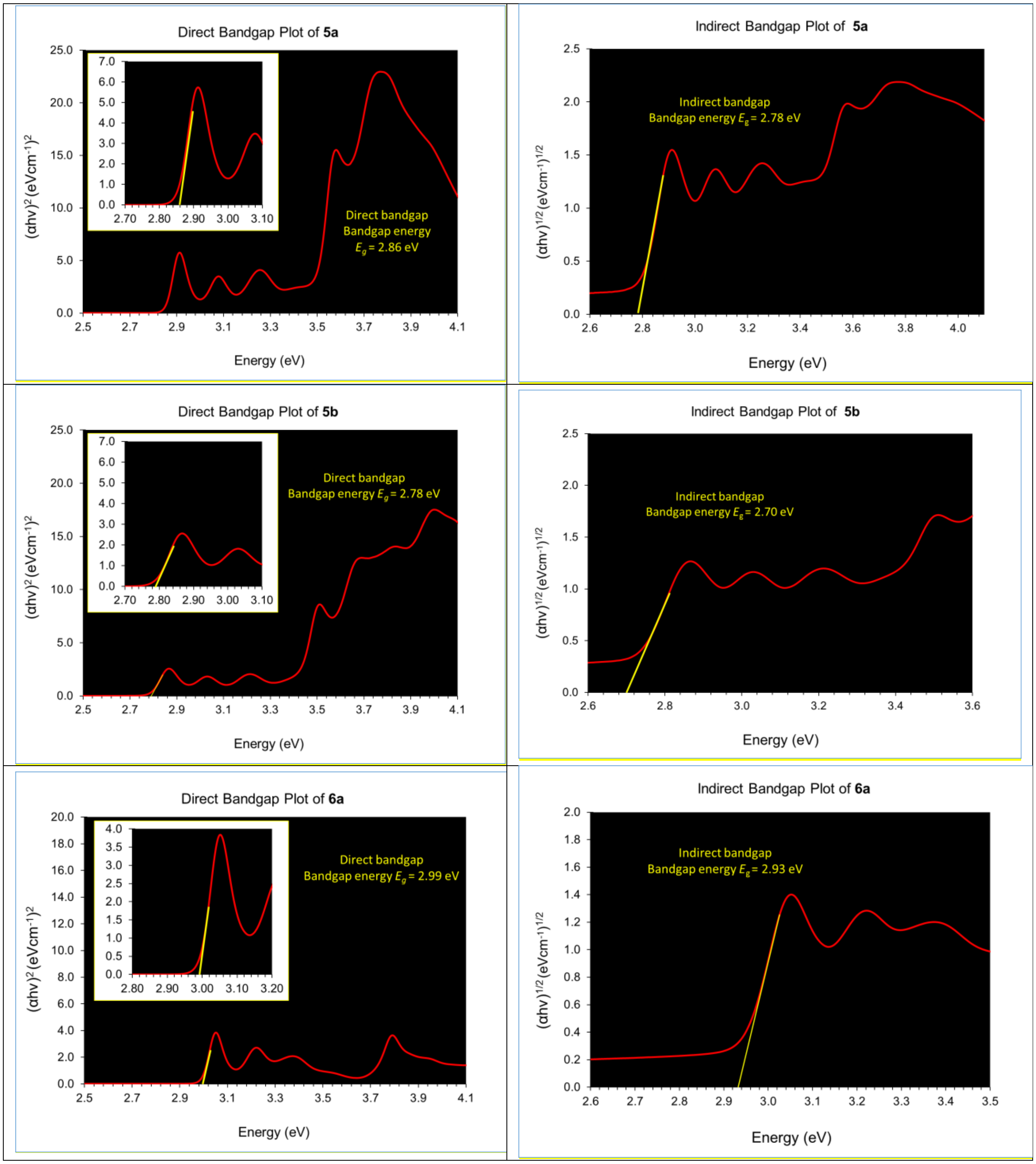

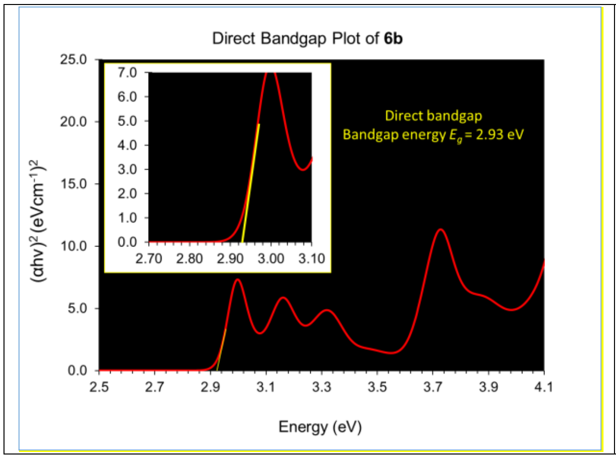
Direct Bandgap Plot of 6b
Direct bandgap
Bandgap energy E_g = 2.93 eV
$(\alpha h\nu)^2$ (eVcm^{-1})^2
Energy (eV)
0.0
5.0
10.0
15.0
20.0
25.0
2.5
2.7
2.9
3.1
3.3
3.5
3.7
3.9
4.1
0.0
1.0
2.0
3.0
4.0
5.0
6.0
7.0
2.70
2.80
2.90
3.00
3.10

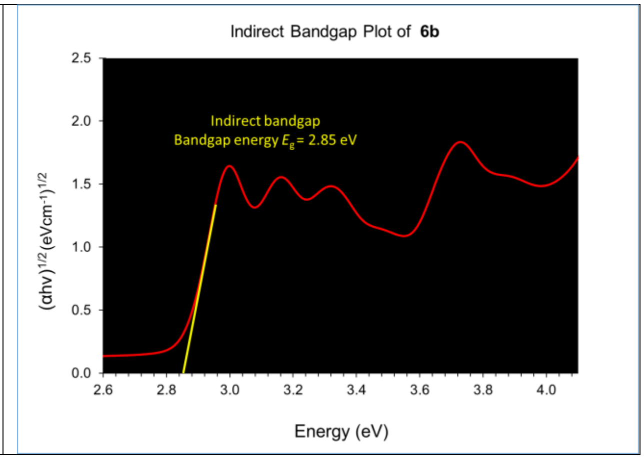
Indirect Bandgap Plot of 6b
Indirect bandgap
Bandgap energy E_g = 2.85 eV
$(\alpha h\nu)^{1/2}$ (eVcm^{-1})$^{1/2}$
Energy (eV)
0.0
0.5
1.0
1.5
2.0
2.5
2.6
2.8
3.0
3.2
3.4
3.6
3.8
4.0
